# Enantioselective synthesis of highly oxygenated acyclic quaternary center-containing building blocks *via* palladium-catalyzed decarboxylative allylic alkylation of cyclic siloxyketones†

**DOI:** 10.1039/d0sc04383d

**Published:** 2020-09-15

**Authors:** Aurapat Ngamnithiporn, Toshihiko Iwayama, Michael D. Bartberger, Brian M. Stoltz

**Affiliations:** Warren and Katharine Schlinger Laboratory for Chemistry and Chemical Engineering, Division of Chemistry and Chemical Engineering, California Institute of Technology Pasadena CA 91125 USA stoltz@caltech.edu; Central Pharmaceutical Research Institute, Japan Tobacco Inc. 1-1, Murasaki-cho, Takatsuki Osaka 569-1125 Japan; 1200 Pharma LLC 844 East Green Street, Suite 204 Pasadena CA 91101 USA

## Abstract

The development of a palladium-catalyzed enantioselective decarboxylative allylic alkylation of cyclic siloxyketones to produce enantioenriched silicon-tethered heterocycles is reported. The reaction proceeds smoothly to provide products bearing a quaternary stereocenter in excellent yields (up to 91% yield) with high levels of enantioselectivity (up to 94% ee). We further utilized the unique reactivity of the siloxy functionality to access chiral, highly oxygenated acyclic quaternary building blocks. In addition, we subsequently demonstrated the utility of these compounds through the synthesis of a lactone bearing vicinal quaternary-trisubstituted stereocenters.

## Abstract 2

The development of a palladium-catalyzed enantioselective decarboxylative allylic alkylation of cyclic siloxyketones to produce enantioenriched silicon-tethered heterocycles is reported.

## Graphical abstract

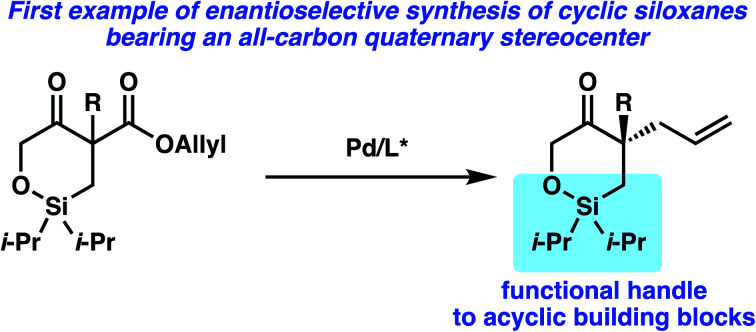

## Introduction

Silicon-containing hetereocycles are an important class of organic molecules often used in the context of temporary functional groups in the synthesis of natural products and drug molecules.^1^ Oxasilacycles or cyclic siloxanes, in particular, are frequently employed in various stages of total syntheses,^2^ characterized not only for their high tolerance to a range of reaction conditions, but also for the selective and facile transformations available for this functionality.

**Scheme 1:**
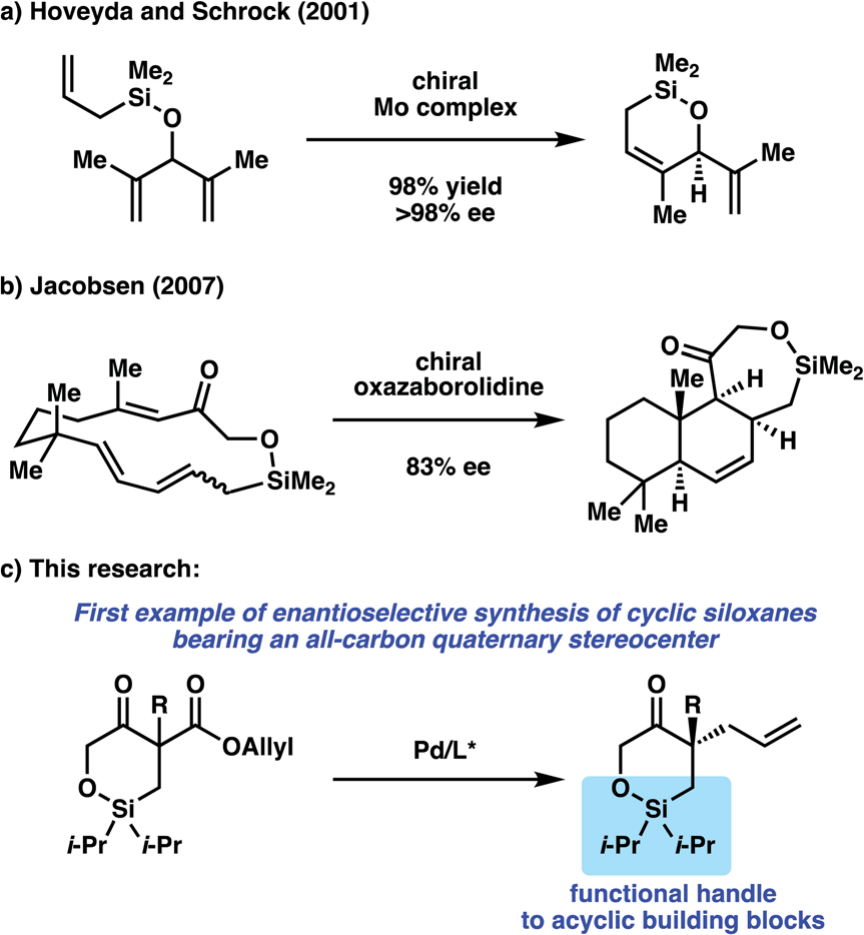
Enantioselective synthesis of cyclic siloxanes.

While a number of non-asymmetric methods have been reported for accessing cyclic siloxanes,^3^ methods to access enantioenriched cyclic siloxanes are limited, with a majority of examples requiring the use of chiral, enantioenriched starting materials.^4^ To the best of our knowledge, there are only two reports detailing the enantioselective synthesis of cyclic siloxanes.^5^ In 2001, Hoveyda, Schrock, and coworkers reported an enantioselective desymmetrization of trienes *via* asymmetric ring-closing metathesis utilizing their developed Mo-catalyst (Scheme 1a).^5*a*^ An enantioenriched cyclic siloxane could be obtained in good yields with excellent enantioselectivity. Jacobsen later disclosed in 2007 a method for asymmetric transannular Diels–Alder Reactions (Scheme 1b).^5*b*^ In the presence of a chiral oxazaborolidine catalyst, the fused cyclic siloxane was obtained with good enantioselectivity. Subsequent oxidative cleavage of this siloxane provided a precursor to a number of sesquiterpine natural products. Although these two reports showcase unique synthetic strategies to access cyclic siloxanes in an enantioselective fashion, the substrate scope in each report only contains one example of a cyclic siloxane. Furthermore, an enantioselective method to access cyclic siloxanes bearing all-carbon quaternary stereocenters has not been reported to date.

To this end, we sought to target the synthesis of this useful motif as part of our on-going research interest in the development of asymmetric allylic alkylation methodologies.^6,7^ Namely, we envision performing an enantioselective decarboxylative allylic alkylation of a cyclic siloxy-β-ketoester to access a siloxyketone bearing a quaternary stereocenter (Scheme 1c).^8^ Furthermore, we believe that utilization of the silicon functionality in the alkylation products would allow for the synthesis of enantioenriched acyclic quaternary stereocenters, a motif that still poses a significant challenge in organic synthesis.^9^ Herein, we report our efforts toward the realization of these goals.

## Results and discussion

**Scheme 2:**
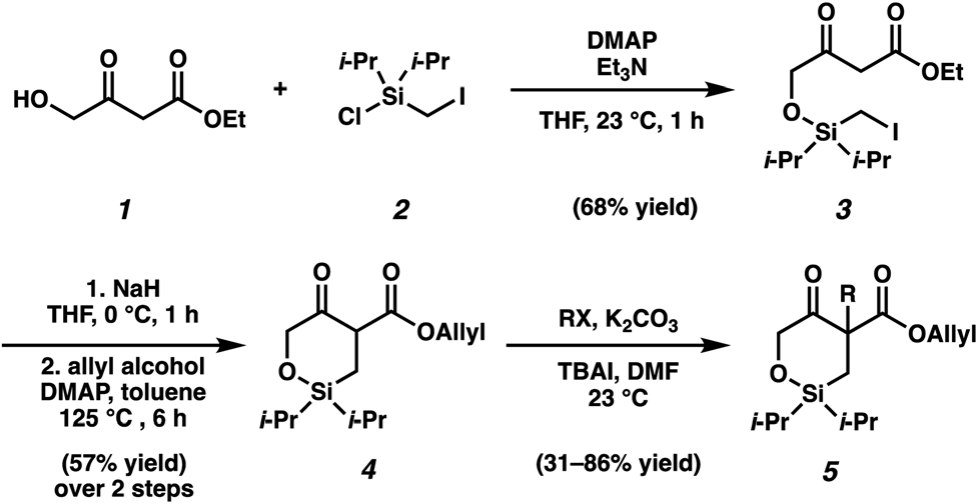
Siloxy-β-ketoester substrates synthesis.

As the synthesis of cyclic siloxy-β-ketoester substrates has not been previously reported, we initiated our study by first establishing a synthetic route to access the cyclic siloxy-β-ketoester substrates (Scheme 2). Our developed process begins with intermolecular *O*-silylation of alcohol **1** with silyl chloride **2**, affording β-ketoester **3**. Cyclization *via* intramolecular nucleophilic substitution and subsequent transesterification with allyl alcohol provided allyl β-ketoester **4** in 57% yield over 2 steps. Lastly, functionalization of **4** with various electrophiles delivered a variety of siloxy-β-ketoester substrates **5**.^10^

With the substrates in hand, our studies continued with an investigation of the palladium-catalyzed decarboxylative allylic alkylation (Table 1). We were pleased to find that treatment of **5a** to commonly employed allylic alkylation conditions^11^ using Pd_2_(dba)_3_ and (*S*)-*t*-BuPHOX ligand (**L1**) in toluene proceeded smoothly with >95% conversion and provided the desired cyclic siloxane **6a** in a moderate 75% ee (Table 1, entry 1). An examination of additional ligands revealed that switching to the more electron-deficient PHOX ligand **L2** delivered the product in an excellent 91% ee (entry 2), while the use of Trost-type bisphosphine ligand **L3** (ref. 12) provided **6a** in a slightly diminished 89% ee (entry 3). Continuing with the optimal ligand **L2**, a survey of different solvents revealed that while consistently high levels of enantioselectivity could be achieved (entries 4–7), toluene remained the optimal solvent. Finally, by performing the reaction at 0 °C, the highest ee of 94% was obtained with no loss in reactivity.

**Table 1:** Optimization of reaction parameters^a^

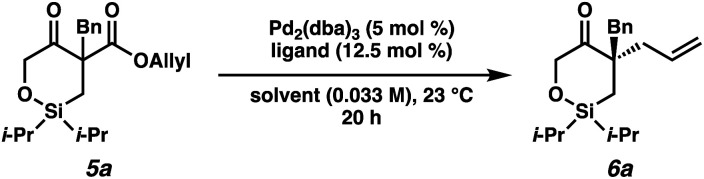				
Entry	Ligand	Solvent	% conv.^b^	% ee^c^
1	**L1**	Toluene	>95	75
2	**L2**	Toluene	>95	91
3	**L3**	Toluene	>95	−89
4	**L2**	THF	>95	87
5	**L2**	MTBE	>95	87
6	**L2**	1,4-Dioxane	>95	89
7	**L2**	2 : 1 hexanes/toluene	>95	89
**8** d	**L2**	**Toluene**	**>95**	**94**
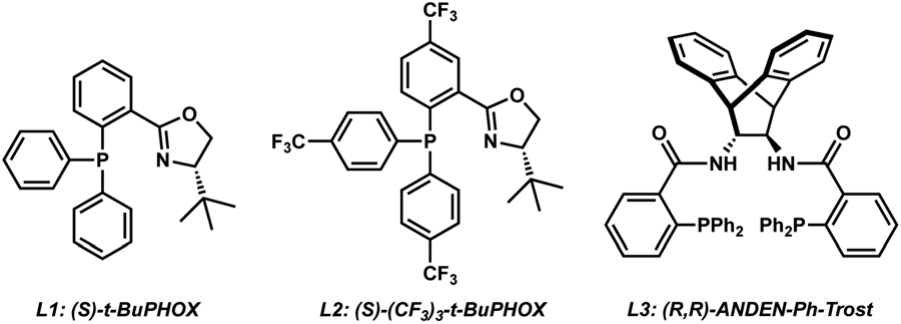				

a Conditions: **5a** (0.1 mmol), Pd_2_(dba)_3_ (5 mol%), ligand (12.5 mol%) in solvent (3 mL).

b Conversion determined by ^1^H NMR of crude reaction mixture using 1,3,5-trimethoxybenzene as a standard.

c %ee determined by chiral SFC analysis of the isolated product.

d Reaction performed at 0 °C.

Having identified the optimal reaction conditions, we next explored the substrate scope of this transformation on 0.2 mmol scale (Table 2). With the benzyl group at the α-quaternary position, the enantioenriched cyclic siloxane product (**6a**) was isolated in 91% yield with 94% ee. An investigation of the electronic nature of this benzyl group demonstrates that both electron-withdrawing and electron-donating groups (**6b–6e**) were well tolerated (90–93% ee). A sterically hindered *ortho*-substituted benzyl substrate fares well under our optimized reaction conditions, providing product **6f** in 83% yield with 92% ee. A significant drop in reactivity, however, was observed when a methyl-substituted substrate was employed (**5g**, *ca.* only 30% conversion). Pleasingly, we were able to improve the reactivity of this substrate by increasing the temperature to 23 °C, delivering product **6g** in 79% yield with 89% ee. Additionally, cyclic siloxane starting materials bearing ester (**5h**) and Boc-protected alkyl amine (**5i**) functionalities delivered products **6h** and **6g** in 94% ee and 90% ee, respectively.

**Table 2:** Substrate scope^a^

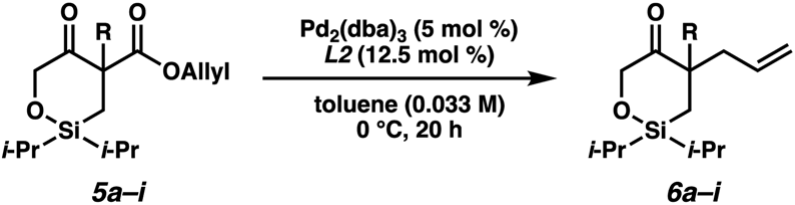
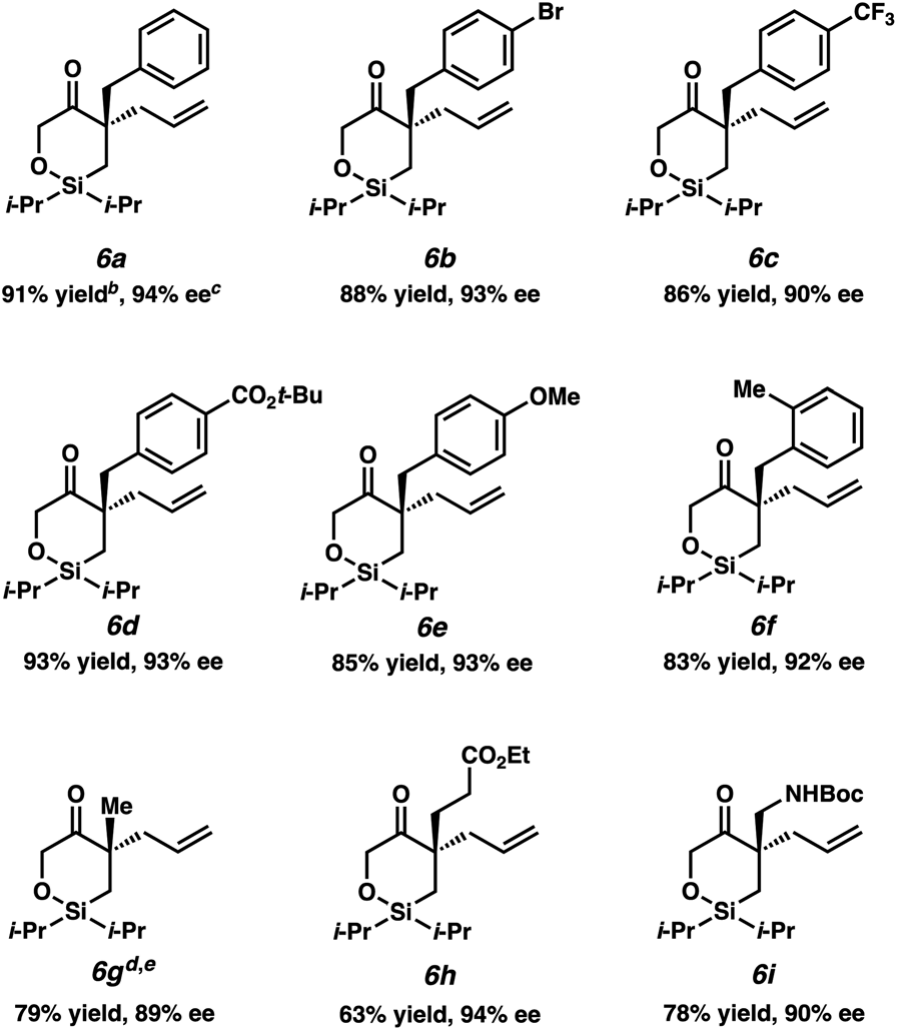

a Reactions performed on 0.2 mmol scale.

b Yield of isolated product.

c % ee determined by chiral SFC analysis.

d Reaction performed at 23 °C.

e Absolute configuration determined by experimental and computed VCD and optical rotation, see text.

Despite several attempts at procuring X-ray quality crystals for determination of the absolute stereochemistry of the products, these efforts proved fruitless, presumably due to the non-polar nature and flexibility of these molecules. Thus, vibrational circular dichroism (VCD) was utilized for the determination of absolute configuration of systems **6a** and **6g** as single enantiomers. Sufficient agreement between the computed and measured VCD spectra were achieved such that the absolute configurations of **6a** and **6g** could be comfortably assigned as (*R*) and (*S*), respectively, as depicted in Table 2.^13^ Additionally, the measured optical rotation of **6g** was found to be consistent with that computed for the Boltzmann-weighted conformational ensemble of the (*S*) enantiomer. With these configurations assigned, the absolute configurations for all other products have been inferred by analogy.

**Scheme 3:**
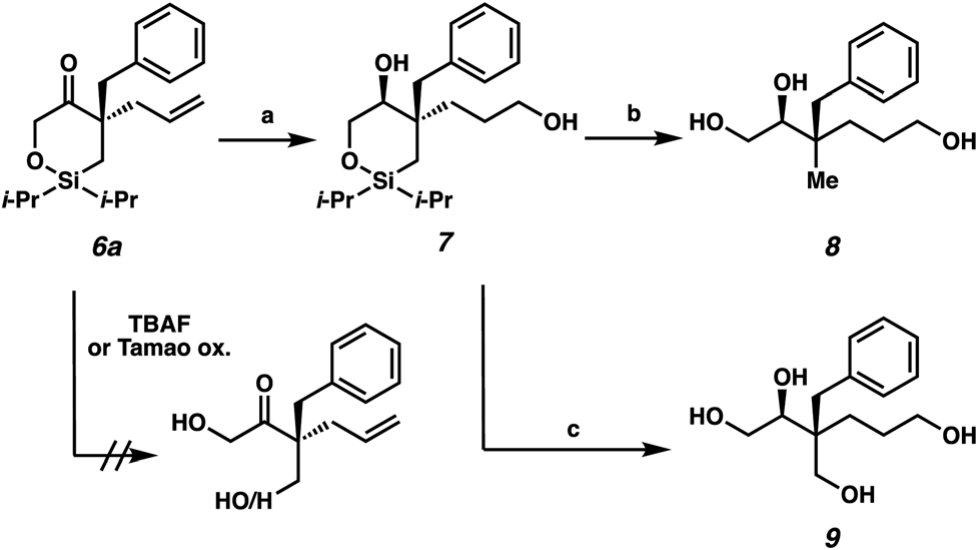
Synthesis of highly oxygenated, chiral, acyclic quaternary stereocenters: (a) BH_3_·SMe_2_, THF, 23 °C, 18 h; H_2_O, 23 °C, 10 min; NaBO_3_·4H_2_O, 23 °C, 4 h, 58% yield, dr = 93 : 7; (b) TBAF, DMF, 80 °C, 18 h, 77% yield; (c) TBAF, NaBO_3_·4H_2_O, THF, 60 °C, 18 h, 51% yield.

The next stage in our study was the utilization of the silyl fragment as a functional handle to access acyclic chiral building blocks (Scheme 3). Unfortunately, the direct ring-opening of cyclic siloxane **6a** proved to be challenging as both neutral (TBAF) and oxidative (Tamao oxidation) conditions failed to deliver the desired acyclic products. However, we were pleased to find that with prior hydoboration/oxidation and concomitant ketone reduction, facile ring opening could be achieved. The diol **7** was isolated in 58% yield as a 93 : 7 mixture of diastereomers.^14^ Siloxane ring opening can then be achieved with TBAF either in the absence or presence of NaBO_3_ as an oxidant to deliver triol **8** or tetraol **9** in 77% yield and 51% yield, respectively.

**Scheme 4:**
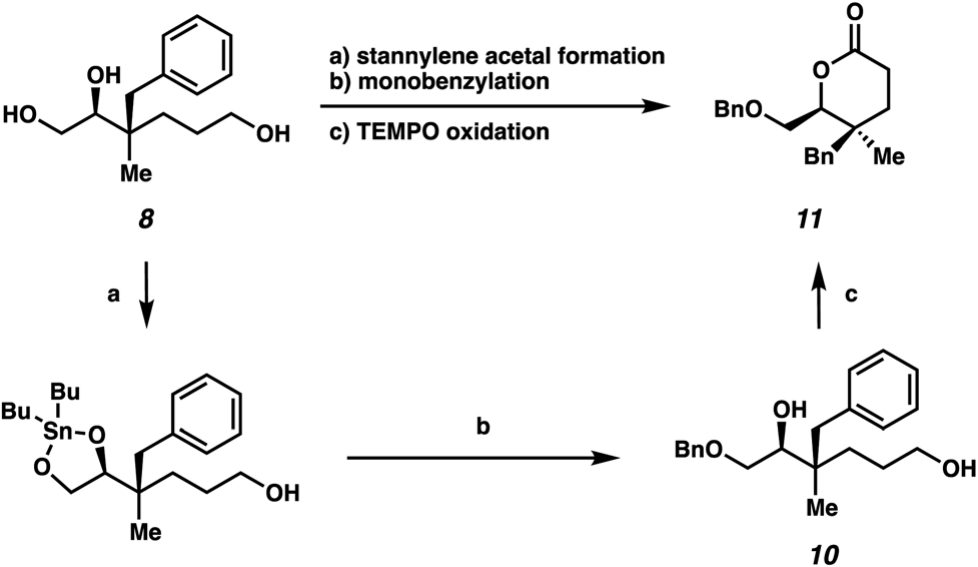
Synthesis of lactone bearing vicinal quaternary-trisubstituted stereocenters: (a) Bu_2_SnO, MeOH, reflux, 18 h; (b) BnBr, TBAB, toluene, reflux, 20 h, 60% yield over 2 steps; (c) TEMPO, PIDA, CH_2_Cl_2_, 23 °C, 18 h, 65% yield.

We also sought to further demonstrate the utility of these highly oxygenated acyclic building blocks *via* the synthesis of a densely functionalized lactone (Scheme 4). First, selective benzylation of triol **8** provided monobenzylated product **10***via* a stannylene acetal intermediate.^15^ Subsequently, δ-lactone **11**, bearing vicinal quaternary-trisubstituted stereocenters, could be generated from TEMPO oxidation in 39% yield over 3 steps.

## Conclusions

In summary, we have developed the first enantioselective synthesis of cyclic siloxanes bearing an all-carbon quaternary stereocenter. Through Pd-catalyzed decarboxylative allylic alkylation, enantioenriched cyclic siloxanes can be constructed in good yield (up to 91% yield) and with high enantiomeric excess (up to 94% ee). The reaction conditions exhibit a high tolerance toward a range of functional groups, and the alkylation products can be further derivatized to obtain stereochemically rich acyclic building blocks. Efforts to further extend the substrate scope of this transformation, as well as applications in natural product synthesis, are currently underway in our laboratory.

## Conflicts of interest

There are no conflicts to declare.

## Supplementary Material

SC-011-D0SC04383D-s001

SC-011-D0SC04383D-s002

SC-011-D0SC04383D-s003
