# Supplementary material for: Enantioselective synthesis of highly oxygenated acyclic quaternary center-containing building blocks via palladium-catalyzed decarboxylative allylic alkylation of cyclic siloxyketones
Source: Chem Sci. 2020 Sep 15;11(40):11068–71. doi: 10.1039/d0sc04383d (PMC8162308; doi:10.1039/d0sc04383d)
Supplement: SC-011-D0SC04383D-s003 [file SC-011-D0SC04383D-s003.pdf]

Electronic Supplementary Material (ESI) for Chemical Science.
This journal is © The Royal Society of Chemistry 2020

Enantioselective Synthesis of Highly Oxygenated Acyclic Quaternary Center-Containing Building Blocks via Palladium-Catalyzed Decarboxylative Allylic Alkylation of Cyclic Siloxyketones

Aurapat Ngamnithiporn,[a,‡] Toshihiko Iwayama,[a,b,‡] Michael D. Bartberger,[c]
and Brian M. Stoltz[a,*]

[a]*Warren and Katharine Schlinger Laboratory of Chemistry and Chemical Engineering*
Division of Chemistry and Chemical Engineering, California Institute of Technology
Pasadena, California 91125, USA
[b]*Central Pharmaceutical Research Institute, Japan Tobacco Inc., 1-1, Murasaki-cho,*
Takatsuki, Osaka, 569-1125, Japan
[c]*1200 Pharma LLC, 844 East Green Street, Suite 204, Pasadena, California 91101, USA*

stoltz@caltech.edu

Table of Contents:

Materials and Methods S2
List of Abbreviations S2
Synthesis of Allyl β-ketoester Starting Materials S3
General Procedure for Catalytic Reactions S9
Spectroscopic Data for Products from Catalytic Reactions S10
Experimental Procedure and Characterization Data for Product Transformations S14
Determination of Enantiomeric Excess S18
Determination of Absolute configuration of **6g** and **6a** via VCD and OR S20
References S24
NMR and IR Spectra of New Compounds S25
SFC Traces of Racemic and Enantioenriched Compounds S79

Materials and Methods

Unless otherwise stated, reactions were performed in flame-dried glassware under an argon or nitrogen atmosphere using dry, deoxygenated solvents. Solvents were dried by passage through an activated alumina column under argon.[1] Reaction progress was monitored by thin-layer chromatography (TLC) or Agilent 1290 UHPLC-MS. TLC was performed using E. Merck silica gel 60 F254 precoated glass plates (0.25 mm) and visualized by UV fluorescence quenching, *p*-anisaldehyde, or $KMnO_4$ staining. Silicycle Silia*Flash*® P60 Academic Silica gel (particle size 40–63 µm) was used for flash chromatography. ^{1}H NMR spectra were recorded on Bruker 400 MHz or Varian Mercury 300 MHz spectrometers and are reported relative to residual $CHCl_3$ (δ 7.26 ppm) or CH_3OH (δ 3.31 ppm). ^{13}C NMR spectra were recorded on Bruker 400 MHz spectrometer (100 MHz) and are reported relative to $CHCl_3$ (δ 77.16 ppm) or CH_3OH (δ 49.00 ppm). ^{19}F NMR spectra were recorded on Varian Mercury 300 MHz spectrometer (282 MHz). Data for ^{1}H NMR are reported as follows: chemical shift (δ ppm) (multiplicity, coupling constant (Hz), integration). Multiplicities are reported as follows: s = singlet, d = doublet, t = triplet, q = quartet, p = pentet, sept = septuplet, m = multiplet, br s = broad singlet, br d = broad doublet, app = apparent. Data for ^{13}C NMR are reported in terms of chemical shifts (δ ppm). IR spectra were obtained using Perkin Elmer Spectrum BXII spectrometer or Nicolet 6700 FTIR spectrometer using thin films deposited on NaCl plates and reported in frequency of absorption (cm^{-1}). Optical rotations were measured with a Jasco P-2000 polarimeter operating on the sodium D-line (589 nm), using a 100 mm path-length cell and are reported as: $[\alpha]_D^T$ (concentration in 10 mg/1 mL, solvent) . Analytical SFC was performed with a Mettler SFC supercritical CO_2 analytical chromatography system utilizing Chiralpak (AD-H, AS-H or IC) or Chiralcel (OD-H, OJ-H, or OB-H) columns (4.6 mm x 25 cm) obtained from Daicel Chemical Industries, Ltd. Analytical chiral GC was performed with an Agilent 6850 GC utilizing a Chiraldex G-TA (30 m x 0.25cm) column (1.0 mL/min carrier gas flow). High resolution mass spectra (HRMS) were obtained from Agilent 6200 Series TOF with an Agilent G1978A Multimode source in electrospray ionization (ESI+), atmospheric pressure chemical ionization (APCI+), or mixed ionization mode (MM: ESI-APCI+), or obtained from Caltech mass spectrometry laboratory.

Reagents were purchased from Sigma-Aldrich, Acros Organics, Strem, or Alfa Aesar and used as received unless otherwise stated.

List of Abbreviations:

ee – enantiomeric excess, SFC – supercritical fluid chromatography, TLC – thin-layer chromatography, IPA – isopropanol, VCD – vibrational circular dichroism, OR – optical rotation, DMAP – 4-dimethylaminopyridine, THF – tetrahydrofuran, DMF – dimthylformamide, TBAI – tetrabutylammonium iodide, dba – dibenzylideneacetone, PHOX – phosphinooxazolines, TBAF – tetrabutylammonium fluoride, TBAB – tetrabutylammonium bromide, TEMPO – (2,2,6,6-tetramethylpiperidin-1-yl)oxyl

Synthesis of Allyl β-ketoester Starting Materials

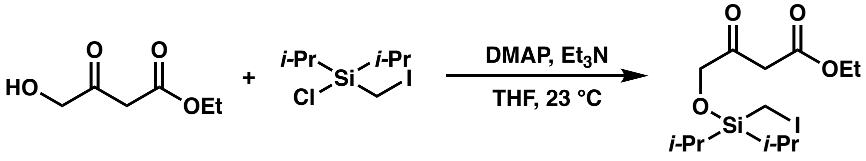

1 *2* *3*

Ethyl 4-(((iodomethyl)diisopropylsilyl)oxy)-3-oxobutanoate (3): To a solution of alcohol **1**[2] (3.14 g, 21.5 mmol, 1.00 equiv) in THF (72 mL) at 0 °C was added chlorosilane **2**[3] (6.25 g, 21.5 mmol, 1.00 equiv), triethylamine (3.0 mL, 21.5 mmol, 1.00 equiv), and DMAP (0.14 g, 1.1 mmol, 0.05 equiv). The reaction mixture was warmed to room temperature and stirred for 1 hour. After complete consumption of the alcohol starting material, as observed by TLC, the reaction was cooled to 0 °C and quenched with saturated aqueous NH_4Cl. The layers were separated and the aqueous layer was extracted with CH_2Cl_2 twice. The combined organic phases were dried over Na_2SO_4, filtered, and concentrated under vacuum. The crude residue was purified by column chromatography (5% to 10% EtOAc in hexanes) to afford **3** as a colorless oil (5.89 g, 68% yield); ^{1}H NMR (400 MHz, $CDCl_3$) δ 4.40 (s, 2H), 4.20 (q, J = 7.2 Hz, 2H), 3.62 (s, 2H), 2.09 (s, 2H), 1.32 – 1.24 (m, 5H), 1.10 (d, J = 4.0 Hz, 6H), 1.08 (d, J = 3.8 Hz, 6H); ^{13}C NMR (100 MHz, $CDCl_3$) δ 203.6, 167.3, 69.6, 61.6, 45.8, 17.6, 17.5, 14.3, 12.3; IR (Neat Film, NaCl) 2944, 2867, 1743, 1726, 1464, 1369, 1320, 1227, 1134, 1109, 1035, 882, 810, 731 cm^{-1}; HRMS (MM) *m/z* calc'd for $C_{13}H_{26}IO_4Si$ $[M+H]^+$: 401.0640, found 401.0625.

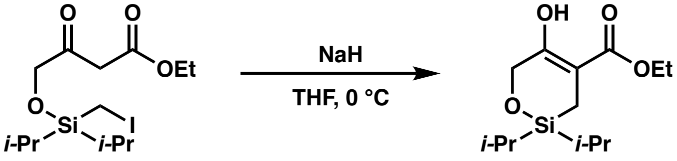

3 *S1*

Ethyl 2,2-diisopropyl-5-oxo-1,2-oxasilinane-4-carboxylate (S1): To a solution of **3** (2.95 g, 7.37 mmol, 1.00 equiv) in THF (74 mL) at 0 °C, NaH (60% in mineral oil, 0.34 g, 8.11 mmol, 1.10 equiv) was added. The reaction mixture was stirred for 1 hour at 0 °C, at which point complete consumption of the starting material was observed by TLC. The reaction was quenched with saturated aqueous NH_4Cl. The layers were separated and the aqueous layer was extracted with EtOAc twice. The combined organics were washed with brine, dried over Na_2SO_4, filtered, and concentrated under vacuum. The crude residue was purified by column chromatography (10% EtOAc in hexanes) to afford **S1** as a colorless oil (1.70 g, 85% yield); ^{1}H NMR (400 MHz, $CDCl_3$) δ 12.50 (s, 1H, OH), 4.44 (d, J = 1.3 Hz, 2H), 4.25 (q, J = 7.1 Hz, 2H), 1.43 (d, J = 1.4 Hz, 2H), 1.34 (t, J = 7.1 Hz, 3H), 1.01 (d, J = 6.1 Hz, 14H); ^{13}C NMR (100 MHz, $CDCl_3$) δ 173.7, 170.6, 95.3, 64.6, 61.0, 17.1, 17.1, 14.4, 12.8, 1.9; IR (Neat Film, NaCl) 2943, 2866, 1655, 1464, 1314, 1221, 1154, 1104, 1048, 882, 785 cm^{-1}; HRMS (MM) *m/z* calc'd for $C_{13}H_{25}O_4Si$ $[M+H]^+$: 273.1517, found 273.1514.

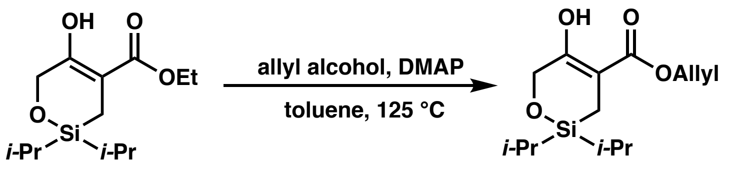

Allyl 2,2-diisopropyl-5-oxo-1,2-oxasilinane-4-carboxylate (4): To a solution of **S1** (0.50 g, 1.84 mmol, 1.00 equiv) in toluene (6 mL) in a sealed tube, allyl alcohol (3.12 mL, 45.9 mmol, 25.0 equiv) and DMAP (22.4 mg, 0.18 mmol, 0.10 equiv) were added. The reaction mixture was heated to 125 °C and stirred for 6 hours. The reaction was allowed to cool to room temperature and concentrated under vacuum. The crude residue was purified by column chromatography (5% EtOAc in hexanes) to afford **4** as a colorless oil (0.38 g, 67% yield); ^{1}H NMR (400 MHz, $CDCl_3$) δ 12.40 (s, 1H, OH), 5.99 (ddt, *J* = 17.2, 10.5, 5.6 Hz, 1H), 5.36 (dq, *J* = 17.2, 1.6 Hz, 1H), 5.28 (dq, *J* = 10.4, 1.3 Hz, 1H), 4.71 (dt, *J* = 5.5, 1.4 Hz, 2H), 4.45 (t, *J* = 1.4 Hz, 2H), 1.47 (t, *J* = 1.4 Hz, 2H), 1.04 – 0.98 (m, 14H); ^{13}C NMR (100 MHz, $CDCl_3$) δ 173.2, 171.1, 132.2, 118.4, 95.2, 65.5, 64.6, 17.1, 17.1, 12.8, 1.9; IR (Neat Film, NaCl) 2943, 2892, 2866, 1656, 1463, 1313, 1216, 1153, 1108, 993, 882, 786, 736 cm^{-1}; HRMS (MM) *m/z* calc'd for $C_{14}H_{25}O_4Si$ $[M+H]^+$: 285.1517, found 285.1519.

Alkylation of β−ketoester

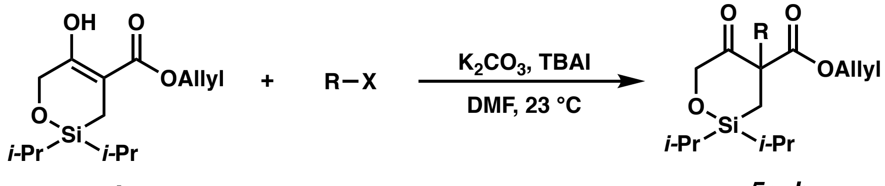

General Procedure 1:

To a solution of β-keto ester **4** (1.00 equiv) in DMF (0.10 M), alkyl halide (1.10 equiv), K_2CO_3 (1.10 equiv), and TBAI (1.10 equiv) were added sequentially. The reaction mixture was stirred at room temperature overnight, then quenched with saturated NH_4Cl. The layers were separated and the aqueous phase was extracted with Et_2O twice. The combined organic layers were dried over Na_2SO_4, filtered, and concentrated. The crude residue was purified by column chromatography to afford alkylated product **5**.

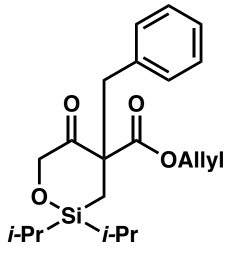

5a

Allyl 4-benzyl-2,2-diisopropyl-5-oxo-1,2-oxasilinane-4-carboxylate (5a)

Prepared according to General Procedure 1 using benzyl bromide and purified by column chromatography (10% Et_2O in hexanes) to afford **5a** as a colorless oil (378 mg, 83% yield). ^1H NMR (400 MHz, $CDCl_3$) δ 7.19 – 7.12 (m, 3H), 7.10 – 7.03 (m, 2H), 5.66 (ddt, *J* = 17.2, 10.4, 5.9 Hz, 1H), 5.21 – 5.08 (m, 2H), 4.50 (ddt, *J* = 13.0, 5.8, 1.3 Hz, 1H), 4.44 (d, *J* = 18.4 Hz, 1H), 4.31 (ddt, *J* = 13.0, 6.0, 1.3 Hz, 1H), 4.20 (dd, *J* = 18.4, 0.6 Hz, 1H), 3.26 (d, *J* = 13.6 Hz, 1H), 2.88 (d, *J* = 13.6 Hz, 1H), 1.67 (d, *J* = 15.2 Hz, 1H), 1.10 – 0.87 (m, 14H), 0.65 (d, *J* = 15.2 Hz, 1H); ^{13}C NMR (100 MHz, $CDCl_3$) δ 208.8, 170.6, 136.5, 131.3, 130.7, 128.1, 126.9, 119.2, 70.3, 66.2, 59.9, 42.5, 17.7, 17.3, 17.1, 17.1, 15.3, 13.4, 12.7; IR (Neat Film, NaCl) 2944, 2867, 1718, 1462, 1269, 1245, 1192, 1093, 995, 883, 781, 743, 701 cm^{-1}; HRMS (MM) *m/z* calc'd for $C_{21}H_{31}O_4Si$ $[M+H]^+$: 375.1986, found 375.1991.

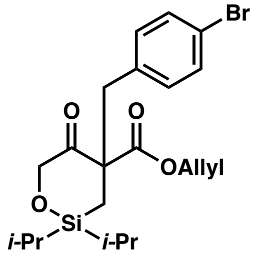

5b

Allyl 4-(4-bromobenzyl)-2,2-diisopropyl-5-oxo-1,2-oxasilinane-4-carboxylate (5b): Prepared according to General Procedure 1 using 4-bromobenzyl bromide and purified by column chromatography (0 to 5% Et_2O in hexanes) to afford **5b** as a colorless oil (154 mg, 38% yield). ^1H NMR (400 MHz, $CDCl_3$) δ 7.36 (d, *J* = 8.4 Hz, 2H), 7.03 (d, *J* = 8.4 Hz, 2H), 5.73 (ddt, *J* = 17.3, 10.4, 5.9 Hz, 1H), 5.27 – 5.17 (m, 2H), 4.56 (ddt, *J* = 13.0, 5.8, 1.4 Hz, 1H), 4.50 (d, *J* = 18.5 Hz, 1H), 4.38 (ddt, *J* = 13.0, 6.1, 1.3 Hz, 1H), 4.26 (d, *J* = 18.4 Hz, 1H), 3.27 (d, *J* = 13.6 Hz, 1H), 2.89 (d, *J* = 13.7 Hz, 1H), 1.71 (d, *J* = 15.2 Hz, 1H), 1.07 – 0.97 (m, 14H), 0.71 (d, *J* = 15.2 Hz, 1H); ^{13}C NMR (100 MHz, $CDCl_3$) δ 208.7, 170.5, 135.5, 132.4, 131.2, 131.1, 121.0, 119.4, 70.2, 66.3, 59.7, 41.9, 17.6, 17.3, 17.1, 17.0, 15.5, 13.4, 12.7; IR (Neat Film, NaCl) 2944, 2895, 2867, 1719, 1488, 1463, 1192, 1094, 995, 883, 777 cm^{-1}; HRMS (MM) *m/z* calc'd for $C_{21}H_{30}BrO_4Si$ $[M+H]^+$: 453.1091, found 453.1066.

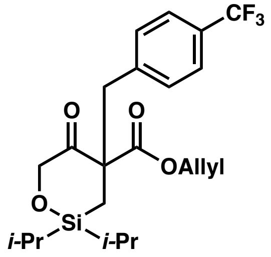

5c

Allyl 2,2-diisopropyl-5-oxo-4-(4-(trifluoromethyl)benzyl)-1,2-oxasilinane-4-carboxylate (5c): Prepared according to General Procedure 1 using 4-(trifluoromethyl)benzyl bromide and purified by column chromatography (0 to 3% Et_2O in hexanes) to afford **5c** as a colorless oil (215 mg, 42% yield). ^{1}H NMR (400 MHz, $CDCl_3$) δ 7.48 (d, *J* = 8.1 Hz, 2H), 7.26 (d, *J* = 8.0 Hz, 2H), 5.65 (ddt, *J* = 17.2, 10.4, 5.9 Hz, 1H), 5.21 – 5.11 (m, 2H), 4.56 – 4.44 (m, 2H), 4.39 – 4.21 (m, 2H), 3.37 (d, *J* = 13.5 Hz, 1H), 2.95 (d, *J* = 13.5 Hz, 1H), 1.72 (d, *J* = 15.1 Hz, 1H), 1.06 – 0.95 (m, 14H), 0.72 (d, *J* = 15.1 Hz, 1H); ^{13}C NMR (100 MHz, $CDCl_3$) δ 208.6, 170.4, 140.8, 131.0, 131.0, 129.2 (q, *J* = 32.0 Hz), 125.0 (q, *J* = 3.8 Hz), 124.4 (q, *J* = 272.4 Hz), 119.5, 70.2, 66.4, 59.8, 42.3, 17.6, 17.3, 17.1, 17.0, 15.8, 13.4, 12.7; ^{19}F NMR (282 MHz, $CDCl_3$) δ -62.50; IR (Neat Film, NaCl) 2945, 2868, 1719, 1463, 1325, 1192, 1165, 1126, 1113, 1068, 1019, 777, 714 cm^{-1}; HRMS (MM) *m/z* calc'd for $C_{22}H_{30}F_3O_4Si$ $[M+H]^+$: 443.1860, found 443.1863.

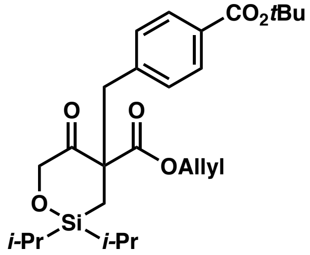

5d

Allyl 4-(4-(*tert*-butoxycarbonyl)benzyl)-2,2-diisopropyl-5-oxo-1,2-oxasilinane-4-carboxylate (5d): Prepared according to General Procedure 1 using *tert*-butyl 4-(bromomethyl)benzoate and purified by column chromatography (5% Et_2O in hexanes) to afford **5d** as a white solid (282 mg, 54% yield). ^{1}H NMR (400 MHz, $CDCl_3$) δ 7.87 (d, *J* = 8.3 Hz, 2H), 7.18 (d, *J* = 8.3 Hz, 2H), 5.76 (ddt, *J* = 17.2, 10.4, 5.9 Hz, 1H), 5.31 – 5.16 (m, 2H), 4.59 (ddt, *J* = 13.0, 5.8, 1.3 Hz, 1H), 4.50 (d, *J* = 18.5 Hz, 1H), 4.40 (ddt, *J* = 13.0, 6.2, 1.3 Hz, 1H), 4.28 (d, *J* = 18.4 Hz, 1H), 3.35 (d, *J* = 13.5 Hz, 1H), 2.99 (d, *J* = 13.6 Hz, 1H), 1.71 (d, *J* = 15.2 Hz, 1H), 1.58 (s, 9H), 1.07 – 0.95 (m, 14H), 0.71 (d, *J* = 15.2 Hz, 1H); ^{13}C NMR (100 MHz, $CDCl_3$) δ 208.5, 170.4, 165.9, 141.3, 131.2, 130.7, 130.5, 129.3, 119.5, 81.0, 70.2, 66.4, 59.8, 42.4, 28.4, 17.7, 17.3, 17.1, 17.1, 15.3, 13.4, 12.7; IR (Neat Film, NaCl) 2941, 2867, 1716, 1611.5, 1462, 1368, 1292, 1254, 1170, 1106, 1020, 778, 758, 706 cm^{-1}; HRMS (MM) *m/z* calc'd for $C_{26}H_{39}O_6Si$ $[M+H]^+$: 475.2510, found 475.2508.

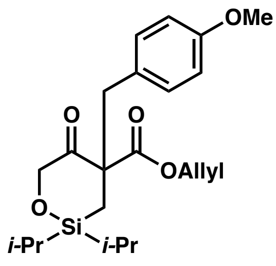

5e

Allyl 2,2-diisopropyl-4-(4-methoxybenzyl)-5-oxo-1,2-oxasilinane-4-carboxylate (5e): Prepared according to General Procedure 1 using 4-(methoxy)benzyl bromide and purified by column chromatography (5 to 15% Et_2O in hexanes) to afford **5e** as a colorless oil (153 mg, 31% yield). ^{1}H NMR (400 MHz, $CDCl_3$) δ 7.08 – 7.03 (m, 2H), 6.81 – 6.76 (m, 2H), 5.75 (ddt, *J* = 17.2, 10.4, 5.9 Hz, 1H), 5.27 – 5.16 (m, 2H), 4.58 (ddt, *J* = 13.0, 5.7, 1.4 Hz, 1H), 4.49 (d, *J* = 18.4 Hz, 1H), 4.39 (ddt, *J* = 13.0, 6.0, 1.3 Hz, 1H), 4.26 (d, *J* = 18.6 Hz, 1H), 3.77 (s, 3H), 3.26 (d, *J* = 13.7 Hz, 1H), 2.90 (d, *J* = 13.8 Hz, 1H), 1.72 (d, *J* = 15.2 Hz, 1H), 1.17 – 0.94 (m, 14H), 0.71 (d, *J* = 15.3 Hz, 1H); ^{13}C NMR (100 MHz, $CDCl_3$) δ 208.9, 170.7, 158.6, 131.6, 131.4, 128.4, 119.1, 113.5, 70.3, 66.2, 59.8, 55.3, 41.7, 17.7, 17.3, 17.1, 17.1, 15.2, 13.4, 12.7; IR (Neat Film, NaCl) 2944, 2867, 1719, 1613, 1514, 1464, 1442, 1271, 1249, 1193, 1178, 1094, 1036, 884, 776, 727, 709 cm^{-1}; HRMS (MM) *m/z* calc'd for $C_{22}H_{33}O_5Si$ $[M+H]^+$: 405.2092, found 405.2093.

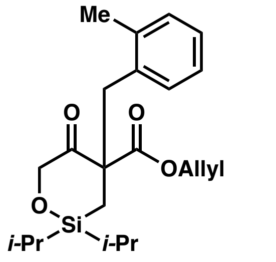

5f

Allyl 2,2-diisopropyl-4-(2-methylbenzyl)-5-oxo-1,2-oxasilinane-4-carboxylate (5f): Prepared according to General Procedure 1 using 2-(methyl)benzyl bromide and purified by column chromatography (5 to 15% Et_2O in hexanes) to afford **5f** as a colorless oil (157 mg, 35% yield). ^{1}H NMR (400 MHz, $CDCl_3$) δ 7.13 – 7.08 (m, 4H), 5.74 (ddt, *J* = 17.2, 10.4, 5.9 Hz, 1H), 5.24 (dq, *J* = 17.2, 1.5 Hz, 1H), 5.21 – 5.17 (m, 1H), 4.60 (ddt, *J* = 13.0, 5.7, 1.4 Hz, 1H), 4.54 (d, *J* = 18.4 Hz, 1H), 4.37 (ddt, *J* = 13.0, 6.2, 1.3 Hz, 1H), 4.31 (d, *J* = 18.5 Hz, 1H), 3.40 (d, *J* = 14.2 Hz, 1H), 3.07 (d, *J* = 14.3 Hz, 1H), 2.27 (s, 3H), 1.74 (d, *J* = 15.1 Hz, 1H), 1.21 – 0.91 (m, 14H), 0.74 (d, *J* = 15.2 Hz, 1H); ^{13}C NMR (100 MHz, $CDCl_3$) δ 208.9, 170.8, 137.3, 134.8, 131.3, 131.3, 130.6, 126.9, 125.6, 119.2, 70.2, 66.3, 59.5, 37.8, 20.1, 17.7, 17.3, 17.1, 17.1, 15.1, 13.4, 12.7; IR (Neat Film, NaCl) 3021, 2944, 2894.4, 2867, 1719, 1463, 1270, 1220, 1191, 1093, 884, 779, 747, 710 cm^{-1}; HRMS (MM) *m/z* calc'd for $C_{22}H_{33}O_4Si$ $[M+H]^+$: 389.2143, found 389.2136.

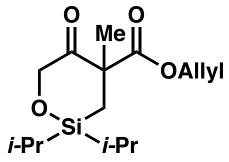

5g

Allyl 2,2-diisopropyl-4-methyl-5-oxo-1,2-oxasilinane-4-carboxylate (5g): Prepared according to General Procedure 1 using methyl iodide, without TBAI, and purified by column chromatography (3 to 15% Et_2O in hexanes) to afford **5g** as a colorless oil (271 mg, 60% yield). ^{1}H NMR (400 MHz, $CDCl_3$) δ 5.88 (ddt, *J* = 17.2, 10.5, 5.7 Hz, 1H), 5.31 (dq, *J* = 17.2, 1.5 Hz, 1H), 5.24 (dq, *J* = 10.5, 1.3 Hz, 1H), 4.68 (ddt, *J* = 13.2, 5.6, 1.4 Hz, 1H), 4.56 (ddt, *J* = 13.2, 5.9, 1.4 Hz, 1H), 4.50 (d, *J* = 18.6 Hz, 1H), 4.30 (d, *J* = 18.6 Hz, 1H), 1.85 (d, *J* = 15.5 Hz, 1H), 1.43 (s, 3H), 1.18 – 0.99 (m, 14H), 0.77 (d, *J* = 15.5 Hz, 1H); ^{13}C NMR (100 MHz, $CDCl_3$) δ 209.7, 172.6, 131.5, 118.9, 70.1, 66.3, 54.7, 24.0, 17.6, 17.3, 17.1, 17.1, 13.3, 12.8; IR (Neat Film, NaCl) 2942, 2896, 1720, 1464, 1262, 1195, 1162, 1124, 1095, 1067, 993, 883, 780, 733, 712 cm^{-1}; HRMS (MM) *m/z* calc'd for $C_{15}H_{27}O_4Si$ $[M+H]^+$: 299.1673, found 299.1669.

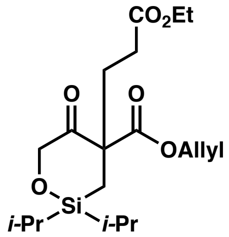

5h

Allyl 4-(3-ethoxy-3-oxopropyl)-2,2-diisopropyl-5-oxo-1,2-oxasilinane-4-carboxylate (5h): Prepared according to General Procedure 1 using ethyl acrylate, without TBAI, and purified by column chromatography (10 to 15% Et_2O in hexanes) to afford **5h** as a colorless oil (150 mg, 32% yield). ^{1}H NMR (400 MHz, $CDCl_3$) δ 5.89 (ddt, *J* = 17.2, 10.5, 5.9 Hz, 1H), 5.33 (dq, *J* = 17.2, 1.5 Hz, 1H), 5.26 (dq, *J* = 10.4, 1.2 Hz, 1H), 4.71 (ddt, *J* = 13.0, 5.7, 1.4 Hz, 1H), 4.59 – 4.53 (m, 1H), 4.50 (d, *J* = 18.5 Hz, 1H), 4.29 – 4.22 (m, 1H), 4.12 (q, *J* = 7.2 Hz, 2H), 2.44–2.19 (m, 3H), 2.08–2.01 (m, 1H), 1.80 (d, *J* = 15.2 Hz, 1H), 1.25 (t, *J* = 7.1 Hz, 3H), 1.11 – 0.95 (m, 14H), 0.70 (d, *J* = 15.2 Hz, 1H); ^{13}C NMR (100 MHz, $CDCl_3$) δ 208.8, 173.1, 171.1, 131.3, 119.4, 70.1, 66.5, 60.7, 57.2, 31.8, 29.7, 17.6, 17.3, 17.1, 17.1, 14.9, 14.4, 13.4, 12.7; IR (Neat Film, NaCl) 2944, 2868, 1737, 1719, 1464, 1377, 1299, 1251, 1192, 1140, 1096, 883, 781, 733, 713 cm^{-1}; HRMS (MM) *m/z* calc'd for $C_{19}H_{33}O_6Si$ $[M+H]^+$: 385.2041, found 385.2045.

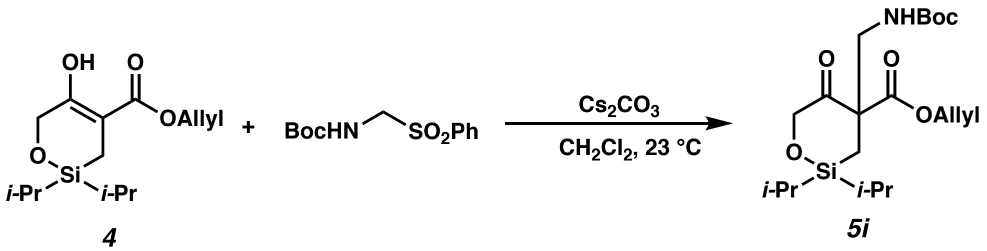

Allyl 4-(((*tert*-butoxycarbonyl)amino)methyl)-2,2-diisopropyl-5-oxo-1,2-oxasilinane-4-carboxylate (5i): To a solution of β-keto ester **4** (275 mg, 0.97 mmol, 1.00 equiv) in CH_2Cl_2 (3.2 mL), sulfonylmethyl carbamate[4] (315 mg, 1.16 mmol, 1.20 equiv) and Cs_2CO_3 (792 mg, 2.43 mmol, 2.5 equiv) were added. The reaction mixture was stirred at room temperature overnight. Upon complete consumption of starting material, as observed by TLC, the reaction was quenched with saturated NH_4Cl. The layers were separated and the aqueous phase was extracted with DCM twice. The combined organic layers were dried over Na_2SO_4, filtered, and concentrated. The crude residue was purified by column chromatography (5 to 10% EtOAc in hexanes) to afford alkylated product **5i** as a colorless viscous oil (344 mg, 86% yield). ^{1}H NMR (400 MHz, $CDCl_3$) δ 5.89 (ddt, *J* = 16.5, 10.4, 5.9 Hz, 1H), 5.32 (dq, *J* = 17.2, 1.5 Hz, 1H), 5.24 (dq, *J* = 10.5, 1.2 Hz, 1H), 5.16 – 5.07 (m, 1H), 4.68 (ddt, *J* = 12.9, 5.8, 1.4 Hz, 1H), 4.58 – 4.45 (m, 2H), 4.26 (d, *J* = 18.8 Hz, 1H), 3.62 (dd, *J* = 13.9, 7.8 Hz, 1H), 3.39 (dd, *J* = 13.8, 5.7 Hz, 1H), 1.73 (d, *J* = 15.5 Hz, 1H), 1.40 (s, 9H), 1.16 – 0.95 (m, 14H), 0.80 (d, *J* = 15.4 Hz, 1H); ^{13}C NMR (100 MHz, $CDCl_3$) δ 210.4, 170.1, 155.9, 131.4, 119.3, 79.5, 70.2, 66.8, 59.3, 46.4, 28.4, 17.5, 17.2, 17.1, 17.0, 13.3, 12.7, 12.3; IR (Neat Film, NaCl) 3465, 2943, 2868, 1749, 1720, 1501, 1464, 366, 1248, 1167, 1106, 1068, 883, 779, 739 cm^{-1}; HRMS (MM) *m/z* calc'd for $C_{20}H_{36}NO_6Si$ $[M+H]^+$: 414.2306, found 414.2312.

Palladium-Catalyzed Asymmetric Allylic Alkylation Reactions

Please note that the absolute configuration was determined only for compounds **6a** and **6g** via vibrational circular dichroism (VCD) and optical rotation. The absolute configuration for all other products has been inferred by analogy. For respective HPLC and SFC conditions, please refer to Table S1.

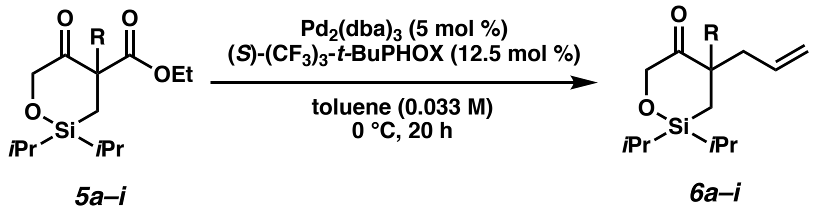

General Procedure 2:

In a nitrogen-filled glovebox, to an oven-dried 4-mL vial equipped with a stir bar was added $Pd_2(dba)_3$ (5 mol %), (*S*)-$(CF_3)_3$-*t*-BuPHOX (12.5 mol %) and toluene (0.003 M based on Pd source). After stirring at room temperature for 30 min, the catalyst mixture was cooled to 0 °C and a solution of starting material **1** in toluene (0.066 M) was then added. The vial was sealed with a PTFE-lined septum cap and stirred at 0 °C. After 20 hours, the vial was removed from the

glovebox. The crude reaction mixture was filtered through a silica plug with Et_2O, concentrated under vacuum, and purified by silica gel flash chromatography to furnish the product.

Spectroscopic Data for Products from Catalytic Reactions

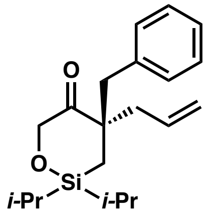

6a

(*R*)-4-allyl-4-benzyl-2,2-diisopropyl-1,2-oxasilinan-5-one (6a): Product **6a** was prepared using General Procedure 2 and purified by column chromatography (5% Et_2O in hexanes) to provide a colorless oil (60.0 mg, 91% yield); 94% ee, $[\alpha]_D^{25}$ +3.47 (*c* 0.92, $CHCl_3$); ^{1}H NMR (400 MHz, $CDCl_3$) δ 7.33 – 7.21 (m, 3H), 7.17 – 7.12 (m, 2H), 5.81 (dddd, *J* = 16.9, 10.3, 7.8, 6.5 Hz, 1H), 5.29 – 5.07 (m, 2H), 4.46 (d, *J* = 18.8 Hz, 1H), 4.42 (d, *J* = 18.8 Hz, 1H), 3.14 (d, *J* = 13.7 Hz, 1H), 2.93 (d, *J* = 13.8 Hz, 1H), 2.52 (ddt, *J* = 14.3, 7.8, 1.2 Hz, 1H), 2.34 (ddt, *J* = 14.4, 6.5, 1.4 Hz, 1H), 1.11 – 1.02 (m, 14H), 0.98 (d, *J* = 1.9 Hz, 2H); ^{13}C NMR (100 MHz, $CDCl_3$) δ 213.8, 137.5, 134.0, 130.7, 128.2, 126.6, 118.9, 71.6, 52.5, 42.5, 41.0, 17.6, 17.5, 17.3, 15.0, 13.6, 13.4; IR (Neat Film, NaCl) 3064, 3029, 2943, 2893, 2866, 1710, 1496, 1464, 1438, 1146, 1100, 918, 883, 789, 736, 702; HRMS (MM) *m/z* calc'd for $C_{20}H_{34}O_2NSi$ $[M+NH_4]^+$: 348.2353, found 348.2353; SFC Conditions: 5% IPA, 2.5 mL/min, Chiralpak AD-H column, λ = 210 nm, t_R (min): major = 2.86, minor = 3.17.

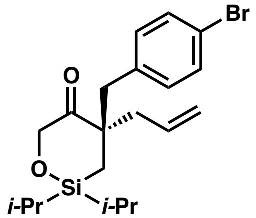

6b

(*R*)-4-allyl-4-(4-bromobenzyl)-2,2-diisopropyl-1,2-oxasilinan-5-one (6b): Product **6b** was prepared using General Procedure 2 and purified by column chromatography (3% Et_2O in hexanes) to provide a colorless oil (72.2 mg, 88% yield); 93% ee, $[\alpha]_D^{25}$ +3.48 (*c* 1.00, $CHCl_3$); ^{1}H NMR (400 MHz, $CDCl_3$) δ 7.44 – 7.33 (m, 2H), 7.05 – 6.97 (m, 2H), 5.73 (dddd, *J* = 16.9, 10.2, 7.7, 6.7 Hz, 1H), 5.19 – 5.07 (m, 2H), 4.39 (s, 2H), 2.98 (d, *J* = 13.8 Hz, 1H), 2.88 (d, *J* = 13.8 Hz, 1H), 2.54 (ddt, *J* = 14.5, 7.7, 1.2 Hz, 1H), 2.27 (ddt, *J* = 14.4, 6.6, 1.4 Hz, 1H), 1.08 – 0.97 (m, 14H), 0.90 (s, 2H); ^{13}C NMR (100 MHz, $CDCl_3$) δ 213.6, 136.6, 133.6, 132.5, 131.3, 120.6, 119.2, 71.5, 52.4, 41.7, 41.0, 17.5, 17.5, 17.3, 17.3, 14.8, 13.6, 13.4; IR (Neat Film, NaCl) 2943, 2866, 1711, 1488, 1463, 1101, 1074, 1012, 882, 785, 756; HRMS (FAB+) *m/z* calc'd for $C_{20}H_{30}BrO_2Si$ $[M+H]^+$: 409.1198, found 409.1187; SFC Conditions: 10% IPA, 2.5 mL/min, Chiralpak AD-H column, λ = 210 nm, t_R (min): major = 3.01, minor = 3.33.

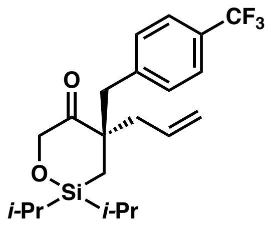

6c

(*R*)-4-allyl-2,2-diisopropyl-4-(4-(trifluoromethyl)benzyl)-1,2-oxasilinan-5-one (6c): Product **6c** was prepared using General Procedure 2 and purified by column chromatography (0 to 5% Et_2O in hexanes) to provide a colorless oil (68.5 mg, 86% yield); 90% ee, $[\alpha]_D^{25}$ –6.05 (*c* 0.99, $CHCl_3$); ^1H NMR (400 MHz, $CDCl_3$) δ 7.52 (d, *J* = 8.0 Hz, 2H), 7.31 – 7.13 (m, 2H), 5.75 (dddd, *J* = 17.0, 10.3, 7.6, 6.7 Hz, 1H), 5.25 – 5.08 (m, 2H), 4.41 (s, 2H), 3.06 (d, *J* = 13.7 Hz, 1H), 3.01 (d, *J* = 13.7 Hz, 1H), 2.58 (ddt, *J* = 14.5, 7.6, 1.3 Hz, 1H), 2.28 (ddt, *J* = 14.5, 6.7, 1.4 Hz, 1H), 1.11 – 0.95 (m, 14H), 0.91 (s, 2H); ^{13}C NMR (100 MHz, $CDCl_3$) δ 213.5, 142.0, 133.5, 131.1, 128.9 (q, *J* = 32.4 Hz), 125.1 (q, *J* = 3.8 Hz), 124.4 (q, *J* = 271.8 Hz), 119.3, 71.5, 52.5, 42.1, 41.2, 17.5, 17.5, 17.3, 17.3, 14.9, 13.6, 13.4; ^{19}F NMR (282 MHz, $CDCl_3$) δ –62.44; IR (Neat Film, NaCl) 2946, 2896, 2868, 1712, 1619, 1464, 1441, 1417, 1326, 1164, 1125, 1069, 1020, 788, 757; HRMS (FAB+) *m/z* calc'd for $C_{21}H_{30}F_3O_2Si$ $[M+H]^+$: 399.1967, found 399.1943; SFC Conditions: 3% IPA, 2.5 mL/min, Chiralpak AD-H column, λ = 210 nm, t_R (min): major = 2.38, minor = 2.96.

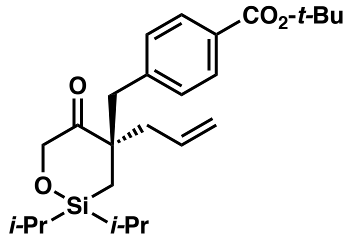

6d

***tert*-butyl (*R*)-4-((4-allyl-2,2-diisopropyl-5-oxo-1,2-oxasilinan-4-yl)methyl)benzoate (6d):** Product **6d** was prepared using General Procedure 2 and purified by column chromatography (5% Et_2O in hexanes) to provide a colorless oil (80.4 mg, 93% yield); 93% ee, $[\alpha]_D^{25}$ +1.44 (*c* 0.99, $CHCl_3$); ^1H NMR (400 MHz, $CDCl_3$) δ 7.88 (d, *J* = 8.3 Hz, 2H), 7.22 – 7.11 (m, 2H), 5.74 (dddd, *J* = 17.0, 10.3, 7.6, 6.7 Hz, 1H), 5.19 – 5.10 (m, 2H), 4.40 (s, 2H), 3.09 (d, *J* = 13.6 Hz, 1H), 2.95 (d, *J* = 13.6 Hz, 1H), 2.55 (ddt, *J* = 14.4, 7.6, 1.2 Hz, 1H), 2.26 (ddt, *J* = 14.4, 6.6, 1.3 Hz, 1H), 1.58 (s, 9H), 1.09 – 0.94 (m, 14H), 0.91 (s, 2H); ^{13}C NMR (100 MHz, $CDCl_3$) δ 213.5, 165.8, 142.6, 133.7, 130.6, 130.3, 130.3, 129.3, 119.2, 81.0, 71.5, 52.5, 42.2, 41.0, 28.3, 17.5, 17.5, 17.3, 15.0, 13.6, 13.4; IR (Neat Film, NaCl) 3402, 3077, 1944, 2895, 2867, 1713, 1611, 1463, 1367, 1293, 1255, 1169, 1117, 1108, 1020, 883, 790, 773, 744; HRMS (MM) *m/z* calc'd for $C_{25}H_{42}O_4NSi$ $[M+NH_4]^+$: 448.2878, found 448.2883; SFC Conditions: 10% IPA, 2.5 mL/min, Chiralpak AD-H column, λ = 210 nm, t_R (min): major = 2.72, minor = 3.12.

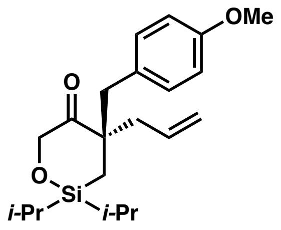

6e

(*R*)-4-allyl-2,2-diisopropyl-4-(4-methoxybenzyl)-1,2-oxasilinan-5-one (6e): Product **6e** was prepared using General Procedure 2 and purified by column chromatography (10% Et_2O in hexanes) to provide a colorless oil (61.8 mg, 85% yield); 93% ee, $[\alpha]_D^{25}$ +3.12 (*c* 0.96, $CHCl_3$); ^1H NMR (400 MHz, $CDCl_3$) δ 7.13 – 6.96 (m, 2H), 6.85 – 6.75 (m, 2H), 5.77 (dddd, *J* = 16.8, 10.3, 7.8, 6.5 Hz, 1H), 5.17 – 5.07 (m, 2H), 4.43 (d, *J* = 19.1 Hz, 1H), 4.38 (d, *J* = 19.1 Hz, 1H), 3.78 (s, 3H), 3.06 (d, *J* = 13.9 Hz, 1H), 2.83 (d, *J* = 13.9 Hz, 1H), 2.46 (ddt, *J* = 14.3, 7.8, 1.2 Hz, 1H), 2.31 (ddt, *J* = 14.3, 6.5, 1.4 Hz, 1H), 1.15 – 0.97 (m, 14H), 0.94 (s, 2H); ^{13}C NMR (100 MHz, $CDCl_3$) δ 214.0, 158.3, 134.1, 131.6, 129.4, 118.8, 113.6, 71.6, 55.3, 52.6, 41.7, 41.0, 17.6, 17.5, 17.3, 17.3, 14.8, 13.5, 13.5; IR (Neat Film, NaCl) 3075, 2944, 2866, 1710, 1612, 1514, 1464, 1441, 1301, 1250, 1179, 1146, 1111, 1099, 1038, 994, 918, 883, 788, 772, 751; HRMS (MM) *m/z* calc'd for $C_{21}H_{36}O_3NSi$ $[M+NH_4]^+$: 378.2459, found 378.2457; SFC Conditions: 5% IPA, 2.5 mL/min, Chiralpak AD-H column, λ = 210 nm, t_R (min): major = 5.04, minor = 5.96.

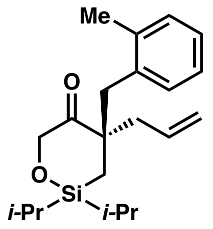

6f

(*R*)-4-allyl-2,2-diisopropyl-4-(2-methylbenzyl)-1,2-oxasilinan-5-one (6f): Product **6f** was prepared using General Procedure 2 and purified by column chromatography (0 to 3% Et_2O in hexanes) to provide a colorless oil (57.0 mg, 83% yield); 92% ee, $[\alpha]_D^{25}$ –0.73 (*c* 0.95, $CHCl_3$); ^1H NMR (400 MHz, $CDCl_3$) δ 7.20 – 7.00 (m, 4H), 5.74 (dddd, *J* = 16.9, 10.3, 7.6, 6.7 Hz, 1H), 5.19 – 5.03 (m, 2H), 4.44 (d, *J* = 18.6 Hz, 1H), 4.38 (d, *J* = 16.9 Hz, 1H), 3.10 (d, *J* = 14.5 Hz, 1H), 3.06 (d, *J* = 14.6 Hz, 1H), 2.51 – 2.43 (m, 2H), 2.31 (s, 3H), 1.13 – 0.93 (m, 16H); ^{13}C NMR (100 MHz, $CDCl_3$) δ 214.0, 137.3, 136.4, 134.1, 130.7, 130.6, 126.5, 125.8, 118.9, 71.7, 53.1, 42.0, 38.5, 20.7, 17.6, 17.5, 17.4, 17.3, 14.2, 13.6, 13.5; IR (Neat Film, NaCl) 3076, 3020, 2943, 2894, 2866, 1710, 1463, 1146, 1098, 994, 918, 883, 791, 742; HRMS (MM) *m/z* calc'd for $C_{21}H_{36}O_2NSi$ $[M+NH_4]^+$: 362.2510, found 362.2514; SFC Conditions: 1% IPA, 2.5 mL/min, Chiralcel OJ-H column, λ = 210 nm, t_R (min): major = 3.96, minor = 4.51.

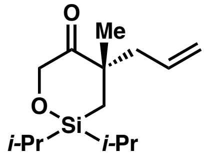

6g

(*S*)-4-allyl-2,2-diisopropyl-4-methyl-1,2-oxasilinan-5-one (6g): Product **6g** was prepared using General Procedure 2 at 23 °C instead of 0 °C and purified by column chromatography (5% Et_2O in hexanes) to provide a pale yellow oil (40.0 mg, 79% yield); 89% ee, $[\alpha]_D^{25}$ –34.54 (*c* 0.95, $CHCl_3$); ^1H NMR (400 MHz, $CDCl_3$) δ 5.72 (dddd, *J* = 16.9, 10.4, 7.8, 6.9 Hz, 1H), 5.14 – 5.02 (m, 2H), 4.40 (d, *J* = 18.6 Hz, 1H), 4.32 (d, *J* = 18.6 Hz, 1H), 2.45 (ddt, *J* = 13.8, 7.7, 1.2 Hz, 1H), 2.33 (ddt, *J* = 13.8, 7.0, 1.3 Hz, 1H), 1.23 (s, 3H), 1.07 – 1.01 (m, 15H), 0.89 (d, *J* = 15.5 Hz, 1H); ^{13}C NMR (100 MHz, $CDCl_3$) δ 215.2, 134.2, 118.5, 70.9, 47.9, 44.2, 25.0, 17.5, 17.5, 17.3, 17.2, 13.5, 13.4; IR (Neat Film, NaCl) 3076, 2943, 2894, 2867, 1713, 1463, 1249, 1164, 1114, 994, 916, 883, 787, 753, 738; HRMS (MM) *m/z* calc'd for $C_{14}H_{27}O_2Si$ $[M+H]^+$: 255.1775, found 255.1768; GC Conditions: G-TA column, 100 °C, isotherm, t_R (min): minor = 98.83, major = 100.76.

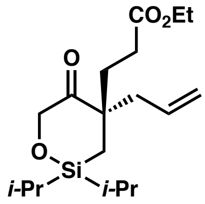

6h

Ethyl (*S*)-3-(4-allyl-2,2-diisopropyl-5-oxo-1,2-oxasilinan-4-yl)propanoate (6h): Product **6h** was prepared using General Procedure 2 and purified by column chromatography (10 to 15% Et_2O in hexanes) to provide a pale yellow oil (43.0 mg, 63% yield); 94% ee, $[\alpha]_D^{25}$ –3.67 (*c* 0.93, $CHCl_3$); ^1H NMR (400 MHz, $CDCl_3$) δ 5.67 (dddd, *J* = 16.2, 10.8, 7.7, 6.8 Hz, 1H), 5.15 – 5.03 (m, 2H), 4.36 (d, *J* = 0.7 Hz, 2H), 4.12 (q, *J* = 7.1 Hz, 2H), 2.53 (ddt, *J* = 14.3, 7.8, 1.2 Hz, 1H), 2.38 – 2.26 (m, 2H), 2.20 – 2.07 (m, 2H), 1.91 (ddd, *J* = 13.9, 11.4, 5.4 Hz, 1H), 1.25 (t, *J* = 7.1 Hz, 3H), 1.11 – 1.01 (m, 14H), 0.97 (d, *J* = 4.4 Hz, 2H). ^{13}C NMR (100 MHz, $CDCl_3$) δ 214.1, 173.5, 133.2, 119.0, 71.2, 60.7, 50.7, 40.7, 31.4, 29.4, 17.5, 17.5, 17.3, 14.9, 14.3, 13.5, 13.5; IR (Neat Film, NaCl) 2943, 2867, 1738, 1710, 1464, 1376, 1303, 1250, 1177, 1104, 918, 883, 788, 749; HRMS (MM) *m/z* calc'd for $C_{18}H_{33}O_4Si$ $[M+H]^+$: 341.2143, found 341.2144; SFC Conditions: 3% IPA, 2.5 mL/min, Chiralpak IC column, λ = 210 nm, t_R (min): minor = 9.30, major = 9.86.

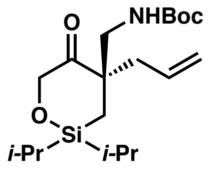

6i

***tert*-butyl (*R*)-((4-allyl-2,2-diisopropyl-5-oxo-1,2-oxasilinan-4-yl)methyl)carbamate (6i):** Product **6i** was prepared using General Procedure 2 and purified by column chromatography (5% EtOAc in hexanes then 5% acetone in hexanes) to provide a colorless oil (57.9 mg, 78% yield); 90% ee, $[\alpha]_D^{25}$ +29.83 (*c* 0.94, $CHCl_3$); ^1H NMR (400 MHz, $CDCl_3$) δ 5.67 (ddt, *J* = 19.2, 9.4, 7.4 Hz, 1H), 5.12 (s, 1H), 5.10 – 5.05 (m, 1H), 4.92 (t, *J* = 6.7 Hz, 1H), 4.34 (d, *J* = 1.2 Hz, 2H), 3.29 (d, *J* = 6.7 Hz, 2H), 2.62 (ddt, *J* = 14.3, 7.5, 1.3 Hz, 1H), 2.45 – 2.33 (m, 1H), 1.40 (s, 9H), 1.13 – 0.91 (m, 16H). ^{13}C NMR (100 MHz, $CDCl_3$) δ 215.6, 156.5, 132.4, 119.5, 79.3, 71.0, 53.1, 46.3, 39.9, 28.5, 17.5, 17.4, 17.2, 13.6, 13.3, 12.1; IR (Neat Film, NaCl) 3465, 3369, 3078, 2943, 2896, 2867, 1708, 1504, 1464, 1366, 1247, 1169, 1109, 883, 787, 751; HRMS (MM) *m/z* calc'd for $C_{19}H_{36}NO_4Si$ $[M+H]^+$: 370.2408, found 370.2409; SFC Conditions: 5% IPA, 2.5 mL/min, Chiralpak AD-H column, λ = 210 nm, t_R (min): minor = 2.80, major = 3.06.

Experimental Procedures and Characterization Data for Product Transformations

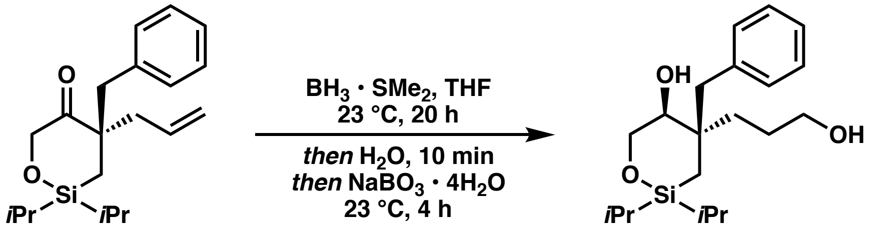

6a ***7***

(4*R*,5*S*)-4-benzyl-4-(3-hydroxypropyl)-2,2-diisopropyl-1,2-oxasilinan-5-ol (7): To a solution of **6a** (257 mg, 0.78 mmol) in THF (3.8 mL) in a 10 mL round-bottom flask was added 2.0 M solution of $BH_3•SMe_2$ in THF (0.78 mL, 1.56 M) at room temperature and stirred for 20 hours. Upon complete consumption of starting material **6a** as observed by TLC, H_2O (1 mL) was added dropwise. After stirring for 10 minutes, $NaBO_3•4H_2O$ (840 mg, 5.45 mmol) was added and the mixture was stirred for an additional 4 hours. The reaction mixture was quenched with 2 M HCl, the organic layer was collected, and the aqueous phase was extracted with EtOAc twice. The organic phases were combined, dried over Na_2SO_4, and concentrated. The crude material was purified by column chromatography (40 to 50% EtOAc in hexanes) to furnish product **7** as a viscous, colorless oil (158 mg, 58% yield) as a 93:7 mixture of diastereomers; $[\alpha]_D^{25}$ –16.53 (*c* 0.38, $CHCl_3$); Major diastereomer: ^1H NMR (400 MHz, $CDCl_3$) δ 7.33 – 7.17 (m, 5H), 4.09 (dd, *J* = 12.3, 1.7 Hz, 1H), 3.89 (dd, *J* = 12.3, 3.9 Hz, 1H), 3.63 (t, *J* = 6.6 Hz, 2H), 3.39 (dd, *J* = 3.5, 1.8 Hz, 1H), 2.92 (d, *J* = 13.3 Hz, 1H), 2.75 (d, *J* = 13.3 Hz, 1H), 1.72 (ddt, *J* = 12.8, 8.3, 4.3 Hz, 2H), 1.42 – 1.15 (m, 2H), 1.15 – 0.97 (m, 14H), 0.83 (d, *J* = 15.2 Hz, 1H), 0.56 (d, *J* = 15.1 Hz, 1H); ^{13}C NMR (100 MHz, $CDCl_3$) δ 138.4, 130.7, 130.7, 128.1, 128.1, 126.2, 72.4, 65.2, 63.3, 43.3, 41.3, 30.9, 26.9, 17.5, 17.5, 17.4, 17.4, 14.2, 13.2, 12.7; IR (Neat Film, NaCl) 3387, 2941, 2865, 1462, 1130, 1054, 882, 828, 733, 703 cm^{-1}; HRMS (MM) *m/z* calc'd for $C_{20}H_{35}O_3Si$ $[M+H]^+$: 351.2350, found 351.2353.

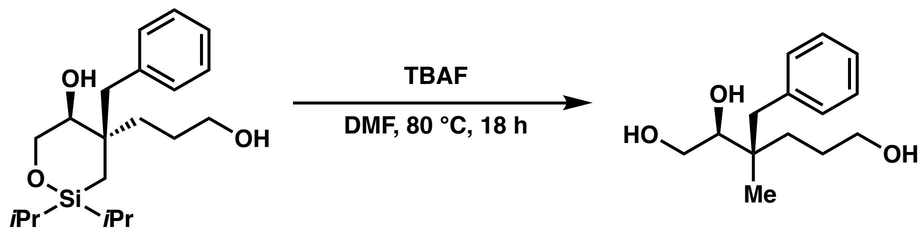

7 8

(2*S*,3*S*)-3-benzyl-3-methylhexane-1,2,6-triol (8): To a solution of **7** (45.5 mg, 0.13 mmol) in DMF (1.3 mL) in a 1-dram vial was added 1.0 M solution of TBAF in THF (0.52 mL, 0.52 mmol) at room temperature and sealed with a Teflon-lined cap. The solution was stirred at 80 °C for 18 hours then 5% aqueous LiCl was added and the organic phase was collected. The aqueous was extracted with EtOAc three times. The organic phases were combined, washed with water then brine, dried over Na_2SO_4, and concentrated. The crude residue was purified by column chromatography (100% EtOAc) to furnish product **8** as a white solid (23.9 mg, 77% yield); $[\alpha]_D^{25}$ –5.10 (*c* 0.18, CH_3OH); ^1H NMR (400 MHz, CD_3OD) δ 7.27 – 7.13 (m, 5H), 3.78 (dd, J = 10.0, 1.3 Hz, 1H), 3.55 – 3.44 (m, 4H), 2.76 (d, J = 13.2 Hz, 1H), 2.62 (d, J = 13.2 Hz, 1H), 1.72 – 1.59 (m, 1H), 1.59–1.47 (m, 1H), 1.39 (ddd, J = 13.6, 12.5, 4.3 Hz, 1H), 1.11 – 1.00 (m, 1H), 0.86 (s, 3H); ^{13}C NMR (100 MHz, $CDCl_3$) δ 140.0, 131.9, 128.7, 126.9, 77.6, 64.0, 63.7, 42.6, 40.9, 32.8, 27.7, 21.1; IR (Neat Film, NaCl) 3363, 2924, 2854, 1660, 1450, 1056, 1016, 830, 702, 683 cm^{-1}; HRMS (ES+) *m/z* calc'd for $C_{14}H_{23}O_3$ $[M+H]^+$: 239.1647, found 239.1633.

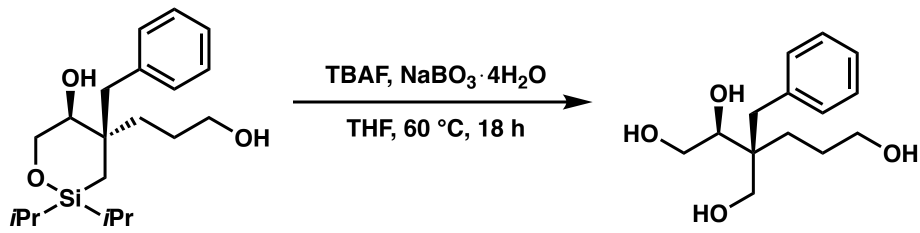

7 9

(2*S*,3*S*)-3-benzyl-3-(hydroxymethyl)hexane-1,2,6-triol (9): To a solution of **7** (13.8 mg, 0.039 mmol) in THF (0.4 mL) in a 1-dram vial was added a 1.0 M solution of TBAF in THF (0.16 mL, 0.16 mmol) and $NaBO_3 \cdot 4H_2O$ (24.2 mg, 0.16 mmol) at room temperature. The vial was sealed with a Teflon-lined cap and the reaction mixture was heated to 60 °C and stirred for 18 hours. Upon complete consumption of starting material by TLC, the reaction mixture was quenched with 2 M HCl. The organic layer was collected and the aqueous phase was extracted with EtOAc twice. The organic phases were combined, washed with brine, dried over Na_2SO_4, and concentrated under vacuum. The crude residue was purified by preparative-TLC (10% MeOH in CH_2Cl_2) to furnish product **9** as a colorless solid (5.0 mg, 51% yield); $[\alpha]_D^{25}$ –0.94 (*c* 0.26, CH_3OH); ^1H NMR (400 MHz, CD_3OD) δ 7.31 – 7.21 (m, 4H), 7.21 – 7.15 (m, 1H), 3.75 (dd, J = 11.5, 3.2 Hz, 1H), 3.65 (dd, J = 11.5, 7.0 Hz, 1H), 3.55 – 3.43 (m, 5H), 2.86 (d, J = 13.4 Hz, 1H), 2.72 (d, J = 13.4 Hz, 1H), 1.77 – 1.65 (m, 1H), 1.65 – 1.53 (m, 1H), 1.30 (ddd, J = 13.8, 12.3, 4.4 Hz, 1H, overlapped with an OH proton), 1.14 – 1.05 (m, 1H).; ^{13}C NMR (100 MHz, CD_3OD) δ 139.3, 131.9, 128.9, 127.1, 77.2, 65.5, 63.9, 63.7, 45.0, 37.9, 28.0, 27.4 ; IR (Neat Film, NaCl) 3339, 2926, 2874, 2860, 1455, 1052, 753, 732, 705, 682 cm^{-1}; HRMS (ES+) *m/z* calc'd for $C_{14}H_{23}O_4$ $[M+H]^+$: 255.1596, found 255.1577.

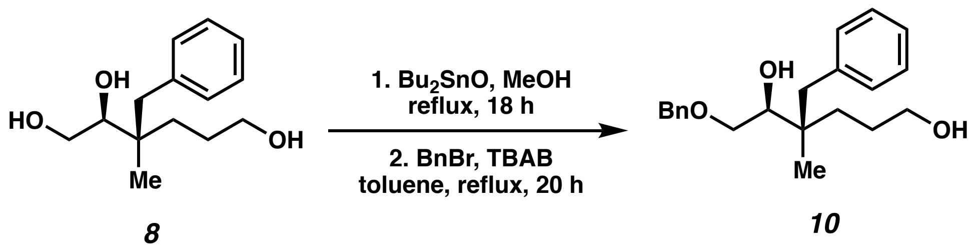

(4*S*,5*S*)-4-benzyl-6-(benzyloxy)-4-methylhexane-1,5-diol (10): To a solution of **8** (10 mg, 0.042 mmol) in MeOH (0.4 mL) in a 1-dram vial was added Bu_2SnO (11 mg, 0.044 mmol) at room temperature. The vial was sealed with a Teflon-lined cap and stirred at 70 °C. After 18 hours, the reaction mixture was concentrated, then redissolved in toluene (0.4 mL). Benzyl bromide (6 uL, 0.05 mmol) and TBAB (16 mg, 0.05 mmol) were added and the reaction was heated to reflux in a sealed vial. After refluxing overnight, the reaction was cooled to room temperature. EtOAc and H_2O were added. The organic layer was collected and the aqueous phase was extracted with EtOAc three times. The organic phases were combined, washed with brine, dried over Na_2SO_4, and concentrated under vacuum. The crude residue was purified by preparative-TLC (50% EtOAc in hexanes) to furnish product **10** as a colorless solid (8.3 mg, 60% yield); $[\alpha]_D^{25}$ 3.29 (*c* 0.23, $CHCl_3$); ^1H NMR (400 MHz, $CDCl_3$) δ 7.40 – 7.29 (m, 5H), 7.28 – 7.22 (m, 2H), 7.22 – 7.15 (m, 3H), 4.56 (d, *J* = 3.6 Hz, 2H), 3.72 – 3.63 (m, 2H), 3.58 (t, *J* = 6.5 Hz, 2H), 3.49 (td, *J* = 9.7, 9.1, 1.9 Hz, 1H), 2.83 (d, *J* = 13.3 Hz, 1H), 2.57 (d, *J* = 13.2 Hz, 1H), 1.73 – 1.46 (m, 2H), 1.39 (ddd, *J* = 13.6, 12.0, 4.6 Hz, 1H), 1.06 (ddd, *J* = 13.6, 12.1, 4.6 Hz, 1H), 0.89 (s, 3H); ^{13}C NMR (100 MHz, $CDCl_3$) δ 138.6, 138.0, 131.0, 128.7, 128.0, 128.0, 127.9, 126.1, 74.0, 73.6, 71.24, 63.7, 41.6, 39.5, 31.5, 26.9, 20.7; IR (Neat Film, NaCl) 3427, 2926, 2870, 1455, 1372, 1256, 1064, 908, 732, 702, 666, 634 cm^{-1}; HRMS (ES+) *m/z* calc'd for $C_{21}H_{29}O_3$ $[M+H]^+$: 329.2117, found 329.2117.

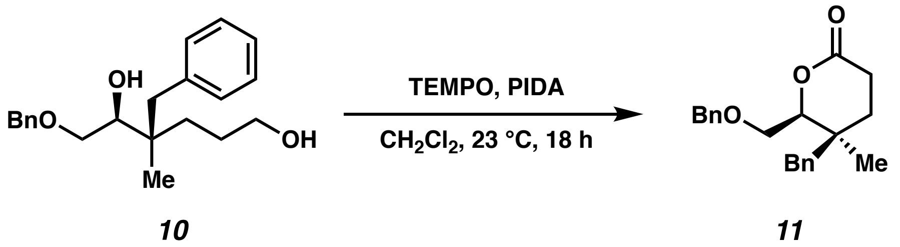

(5*S*,6*S*)-5-benzyl-6-((benzyloxy)methyl)-5-methyltetrahydro-2*H*-pyran-2-one (11): To a solution of **10** (8.3 mg, 0.025 mmol) in CH_2Cl_2 (0.3 mL) in a 1-dram vial was added (diacetoxyiodo)benzene (PIDA) (25.7 mg, 0.08 mmol) and TEMPO (1.0 mg, 0.005 mmol) at room temperature. The vial was sealed with a Teflon-lined cap and the solution was stirred for 18 hours. At this point, complete consumption of starting material by TLC was observed, and the reaction was quenched with saturated aqueous $Na_2S_2O_3$. Additional Et_2O was added, the organic phase was collected, and the aqueous phase was extracted with Et_2O three times. The organic phases were combined, washed with brine, dried over Na_2SO_4, and concentrated under vacuum. The crude residue was purified by preparative-TLC (50% EtOAc in hexanes) to furnish product **11** as a colorless solid (5.3 mg, 65% yield); $[\alpha]_D^{25}$ 14.81 (*c* 0.51, $CHCl_3$); ^1H NMR (300 MHz, $CDCl_3$) δ 7.42 – 7.19 (m, 8H), 7.16 – 7.06 (m, 2H), 4.61 (d, *J* = 1.6 Hz, 2H), 4.29 (dd, *J* = 5.0, 3.6 Hz, 1H), 3.82 (dd, *J* = 4.3, 3.3 Hz, 2H), 2.78 – 2.47 (m, 4H), 1.88 (ddd, *J* = 14.1, 7.9, 6.4 Hz, 1H), 1.42 (dt, *J* = 14.4, 7.6 Hz, 1H), 1.03 (s, 3H); ^{13}C NMR (100 MHz, $CDCl_3$) δ 171.5, 137.8, 136.7, 130.6, 128.6, 128.3, 128.0, 127.9, 126.8, 68.9, 73.9, 69.4, 39.7, 35.3, 29.9, 27.2, 23.7; IR (Neat Film,

NaCl) 2927, 1732, 1454, 1354, 1256, 1202, 1166, 1074, 730, 700, 636 cm^{-1}; HRMS (ES+) *m/z* calc'd for $C_{21}H_{25}O_3$ $[M+H]^+$: 325.1804, found 325.1801.

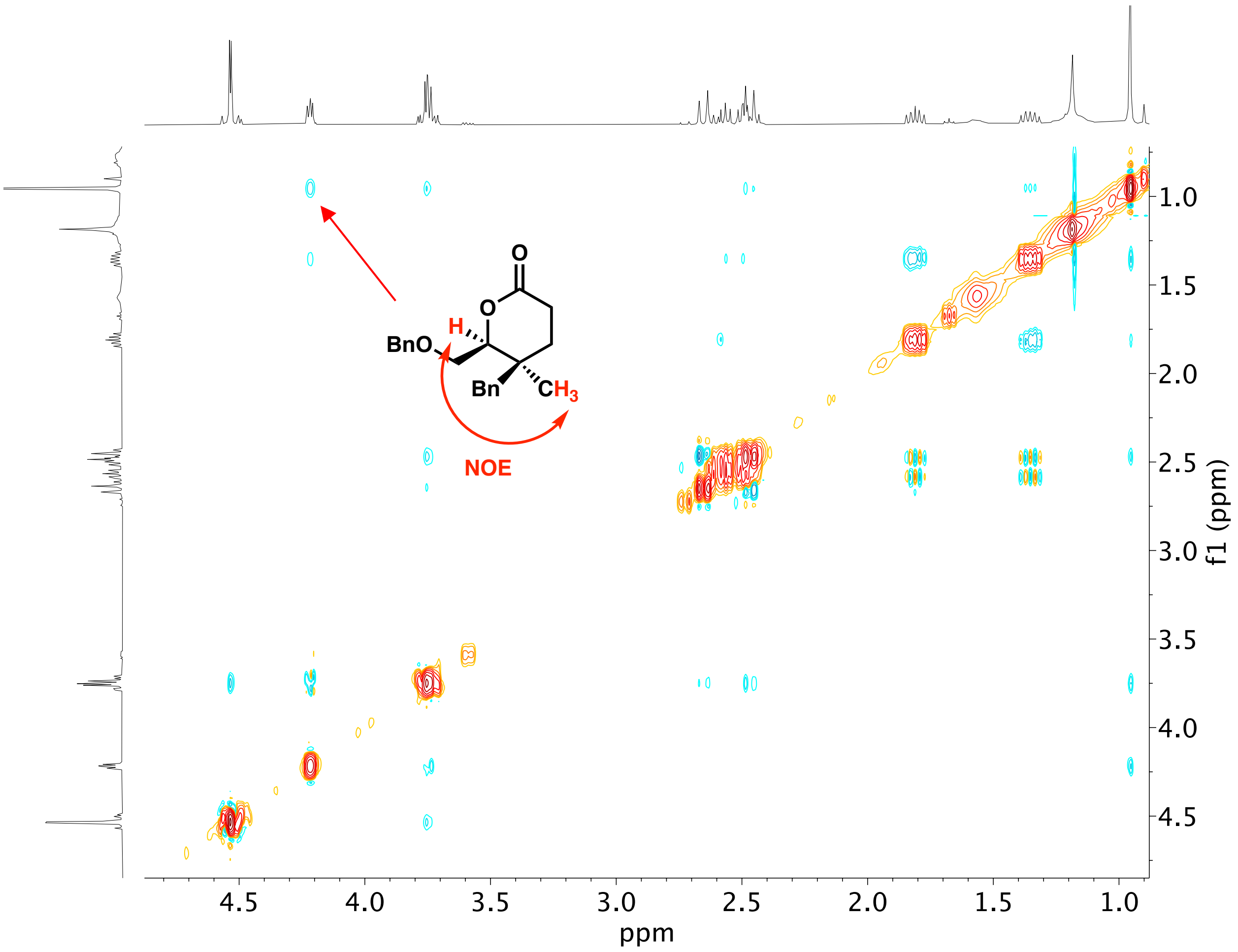

Table S1. Determination of Enantiomeric Excess

entry	compound	SFC analytic conditions	ee (%)
1	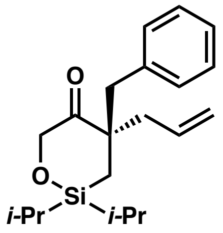 ***6a***	Chiralpak AD-H, λ = 210 nm<br>5% IPA/CO_2, 2.5 mL/min<br>t_R (min) major 2.86, minor 3.17	94
2	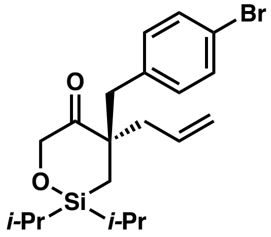 ***6b***	Chiralpak AD-H, λ = 210 nm<br>10% IPA/CO_2, 2.5 mL/min<br>t_R (min) major 3.01, minor 3.33	93
3	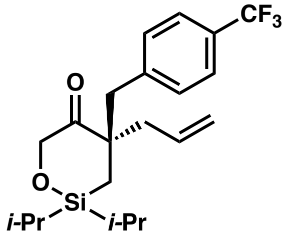 ***6c***	Chiralpak AD-H, λ = 210 nm<br>3% IPA/CO_2, 2.5 mL/min<br>t_R (min) major 2.38, minor 2.96	90
4	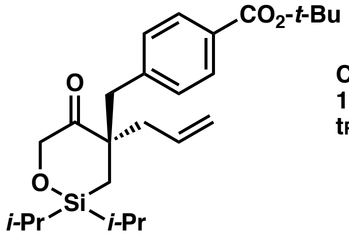 ***6d***	Chiralpak AD-H, λ = 210 nm<br>10% IPA/CO_2, 2.5 mL/min<br>t_R (min) major 2.72, minor 3.12	93

entry	compound	analytic conditions	ee (%)
5	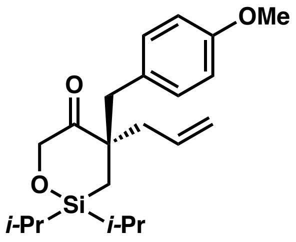 **6e**	SFC, Chiralpak AD-H, λ = 210 nm<br>5% IPA/CO_2, 2.5 mL/min<br>t_R (min) major 5.04, minor 5.96	93
6	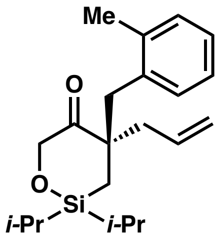 **6f**	SFC, Chiralcel OJ-H, λ = 210 nm<br>10% IPA/CO_2, 2.5 mL/min<br>t_R (min) major 3.96, minor 4.51	92
7	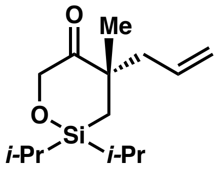 **6g**	GC, G-TA column<br>100 °C, isotherm<br>t_R (min) minor 98.83, major 100.76	89
8	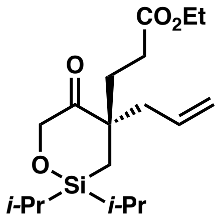 **6h**	SFC, Chiralpak IC, λ = 210 nm<br>3% IPA/CO_2, 2.5 mL/min<br>t_R (min) minor 9.30, major 9.86	94
9	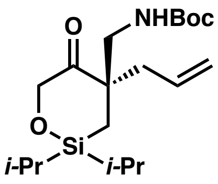 **6h**	SFC, Chiralpak AD-H, λ = 210 nm<br>5% IPA/CO_2, 2.5 mL/min<br>t_R (min) minor 2.80, major 3.06	90

Determination of Absolute Configuration of 6g via VCD and OR

Method 1 – Vibrational Circular Dichroism (VCD)

Experimental Protocol. A solution of **6g** (30 mg/mL) was prepared in $CDCl_3$ and loaded into a front-loading SL-4 cell (International Crystal Laboratories) possessing BaF_2 windows and 100 μm path length. Infrared (IR) and VCD spectra were acquired on a BioTools ChiralIR-2X VCD spectrometer as a set of set of 30 one-hour blocks (30 blocks, 3120 scans per block) in dual PEM mode. A 15-minute acquisition of neat (+)-α-pinene control (separate 75 μm BaF_2 cell) yielded a VCD spectrum in agreement with literature spectra. IR and VCD spectra were background-corrected using a 30-minute block IR acquisition of the empty instrument chamber under gentle N_2 purge, and were solvent corrected using a 4-hour (4 blocks, 3120 scans per block) IR/VCD acquisition of $CDCl_3$ in the same 100 μm BaF2 cell. The reported spectra represent the result of block averaging.

Computational Protocol. The arbitrarily chosen (*S*) enantiomer of compound **6g** was subjected to an exhaustive initial molecular mechanics-based conformational search (MMFF94 force field, 0.08 Å geometric RMSD cutoff, and 30 kcal/mol energy window) as implemented in MOE 2019.0102 (Chemical Computing Group, Montreal, CA). All conformers retained the (*S*) configuration. All MMFF94 conformers under a 10 kcal/mol energy window were then subjected to geometry optimization, harmonic frequency calculation, and VCD rotational strength evaluation using density functional theory. All quantum mechanical calculations first utilized the B3LYP functional, small 6-31G* basis, and IEFPCM model (chloroform solvent) as an initial filter, followed by subsequent optimization using B3PW91 functional, cc-pVTZ basis, and implicit IEFPCM chloroform solvation model on all IEFPCM-B3LYP/6-31G* conformers below 5 kcal/mol. All calculations were performed with the *Gaussian 16* program system (Rev. C.01; Frisch *et al*., Gaussian, Inc., Wallingford, CT). Resultant IEFPCM-B3PW91/cc-pVTZ harmonic frequencies were scaled by 0.98. All structurally unique conformers possessing all positive Hessian eigenvalues were Boltzmann weighted by relative free energy at 298.15 K. The predicted IR and VCD frequencies and intensities of the retained conformers were convolved using Lorentzian line shapes ($\gamma = 4\ cm^{-1}$) and summed using the respective Boltzmann weights to yield the final predicted IR and VCD spectra of the (*S*) enantiomer of **6g**. The predicted VCD of the corresponding (*R*) enantiomer was generated by inversion of sign. From the reasonable agreement between the predicted and measured IR and VCD spectra in the useful range (1000-1400 cm^{-1}; see below) supported by a separate, optical rotation-based assignment (see Method 2) the absolute configuration of **6g** was established as (*S*).

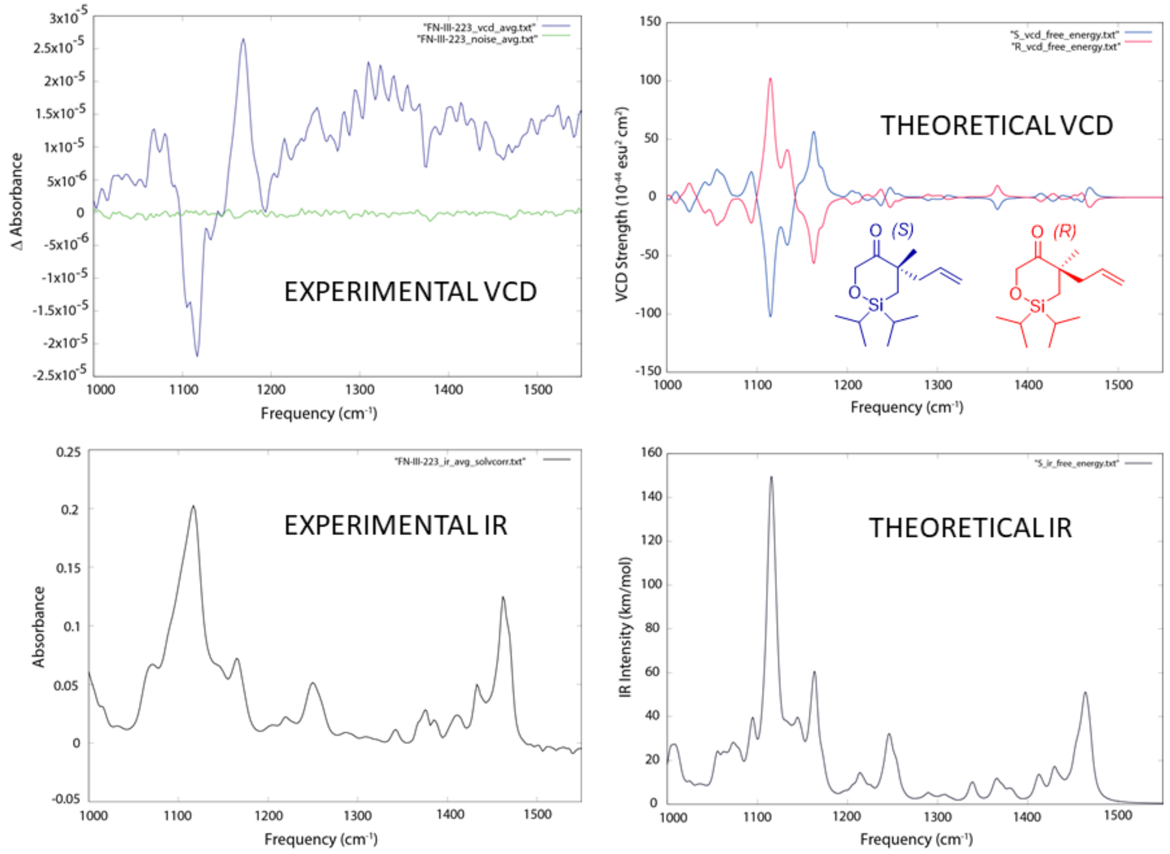

Experimental (left) and computed (right) IR and VCD spectra for **6g.**

Method 2 – Optical Rotation (OR)

Computational Protocol. The ensemble of unique IEFPCM-B3PW91/cc-pVTZ conformers of the *(S)*-enantiomer of **6g** generated in Method 1 above were subjected to optical rotation calculation at 589.0 nm using the B3LYP hybrid density functional, the large and diffuse 6-311++G(2df,2pd) basis set, and the IEFPCM implicit chloroform solvent model. From the computed IEFPCM-B3PW91/cc-pVTZ free energies at 298.15 K and IEFPCM-B3LYP/6-31++G(2df,2pd) optical rotations, a Boltzmann-weighted OR value of –34.7° was determined for the *(S)*-configuration **6g**. (Weighted OR values instead using IEFPCM-B3PW91/cc-pVTZ total energies or IEFPCM-B3LYP/6-31++G(2df,2pd)//IEFPCM-B3PW91/cc-pVTZ total energies were found to be –42.5° and –39.2°, respectively).

As the measured optical rotation of **6g** was found to be **–34.54**° ($CHCl_3$ solvent, 25 °C, c = 0.95), the absolute configuration of **6g** is therefore assigned as *(S)*, consistent with the configuration assigned using VCD method. The individual relative energies, free energies, and optical rotational signatures of each conformer are provided in the accompanying Microsoft Excel file.

Determination of Absolute Configuration of 6a via VCD and OR

Method 1 – Vibrational Circular Dichroism (VCD)

Experimental Protocol. A solution of **6a** (55 mg/mL) was prepared in $CDCl_3$ and loaded into a front-loading SL-4 cell (International Crystal Laboratories) possessing BaF_2 windows and 100 µm path length. Infrared (IR) and VCD spectra were acquired on a BioTools ChiralIR-2X VCD spectrometer as a set of set of 30 one-hour blocks (30 blocks, 3120 scans per block) in dual PEM mode. A 15-minute acquisition of neat (-)-α-pinene control (separate 75 µm BaF_2 cell) yielded a VCD spectrum in agreement with literature spectra. IR and VCD spectra were background-corrected using a 30-minute block IR acquisition of the empty instrument chamber under gentle N_2 purge, and were solvent corrected using a 4-hour (4 blocks, 3120 scans per block) IR/VCD acquisition of $CDCl_3$ in the same 100 µm BaF_2 cell as that used for sample acquisition. The reported spectra represent the result of block averaging.

Computational Protocol. The arbitrarily chosen (*R*) enantiomer of compound **6a** was subjected to an exhaustive initial molecular mechanics-based conformational search (MMFF94 force field, 0.08 Å geometric RMSD cutoff, and 30 kcal/mol energy window) as implemented in MOE 2019.0102 (Chemical Computing Group, Montreal, CA). All conformers retained the (*R*) configuration. All MMFF94 conformers under a 10 kcal/mol energy window were then subjected to geometry optimization, harmonic frequency calculation, and VCD rotational strength evaluation using density functional theory. All quantum mechanical calculations first utilized the B3LYP functional, small 6-31G* basis, and IEFPCM model (chloroform solvent) as an initial filter, followed by subsequent optimization using B3PW91 functional, cc-pVTZ basis, and implicit IEFPCM chloroform solvation model on all IEFPCM-B3LYP/6-31G* conformers below 3 kcal/mol. All calculations were performed with the *Gaussian 16* program system (Rev. C.01; Frisch *et al.*, Gaussian, Inc., Wallingford, CT). Resultant IEFPCM-B3PW91/cc-pVTZ harmonic frequencies were scaled by 0.98. All structurally unique conformers possessing all positive Hessian eigenvalues were Boltzmann weighted by relative free energy at 298.15 K. The predicted IR and VCD frequencies and intensities of the retained conformers were convolved using Lorentzian line shapes ($\gamma = 4\ cm^{-1}$) and summed using the respective Boltzmann weights to yield the final predicted IR and VCD spectra of the (*R*) enantiomer of **6a**. The predicted VCD of the corresponding (*S*) enantiomer was generated by inversion of sign. From the reasonable agreement between the

predicted and measured IR and VCD spectra in the useful range (1050-1375 cm^{-1}; see below) the absolute configuration of **6a** is proposed, with moderate confidence, to likely be (*R*).

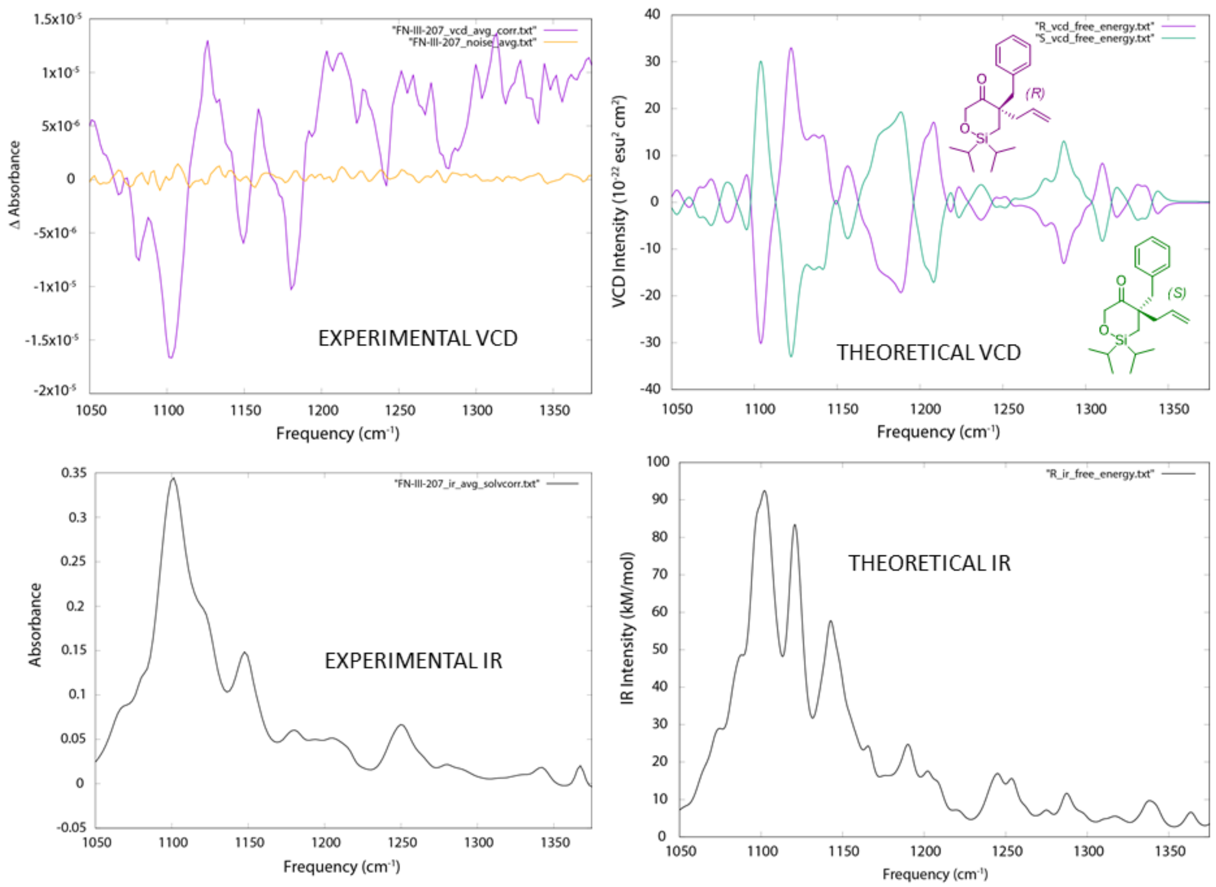

Experimental (left) and computed (right) IR and VCD spectra for **6a.**

(Attempted) Method 2 – Optical Rotation (OR)

Computational Protocol. The ensemble of unique IEFPCM-B3PW91/cc-pVTZ conformers of the *(R)* enantiomer of **6a** generated in Method 1 above were subjected to optical rotation calculation at 589.0 nm using the B3LYP hybrid density functional, the large and diffuse 6-311++G(2df,2pd) basis set, and the IEFPCM implicit chloroform solvent model. Unlike the case of **6g**, the geometric and energetic characteristics of **6a** (including a preponderance of boat-like structures among the lowest energy conformers) give rise to individual, per-conformer optical rotations which fluctuate too greatly in sign to be confidently weighted and summed in order to compare with experiment (for which the measured value of +3.47° is also too close to zero to be compared to theory with any degree of confidence). The individual relative energies, free energies, and optical rotational signatures of each conformer of **6a** (again, *not* used for assignment of **6a**, but provided for transparency) are provided in the accompanying Microsoft Excel file.

References:
[1] Pangborn, A. M.; Giardello, M. A.; Grubbs, R. H.; Rosen, R. K.; Timmers, F. J. *Organometallics* **1996**, 15, 1518–1520.
[2] Miller, D. J.; Yu, F.; Knight, D. W.; Allemann, R. K. *Org. Biomol. Chem.* **2009**, 7, 962–975.
[3] Parasram, M.; Iaroshenko, V. O.; Gevorgyan, V. *J. Am. Chem. Soc.* **2014**, 52, 17926–17929.
[4] (a) Klepacz, A.; Zwierzak, A. *Tetrahedron Lett*. **2002**, 43, 1079–1080. (b) Sikriwal, D.; Kant, R.; Maulik, P. R.; Dikshit, D. K. *Tetahedron* **2010**, 66, 6167–6173.

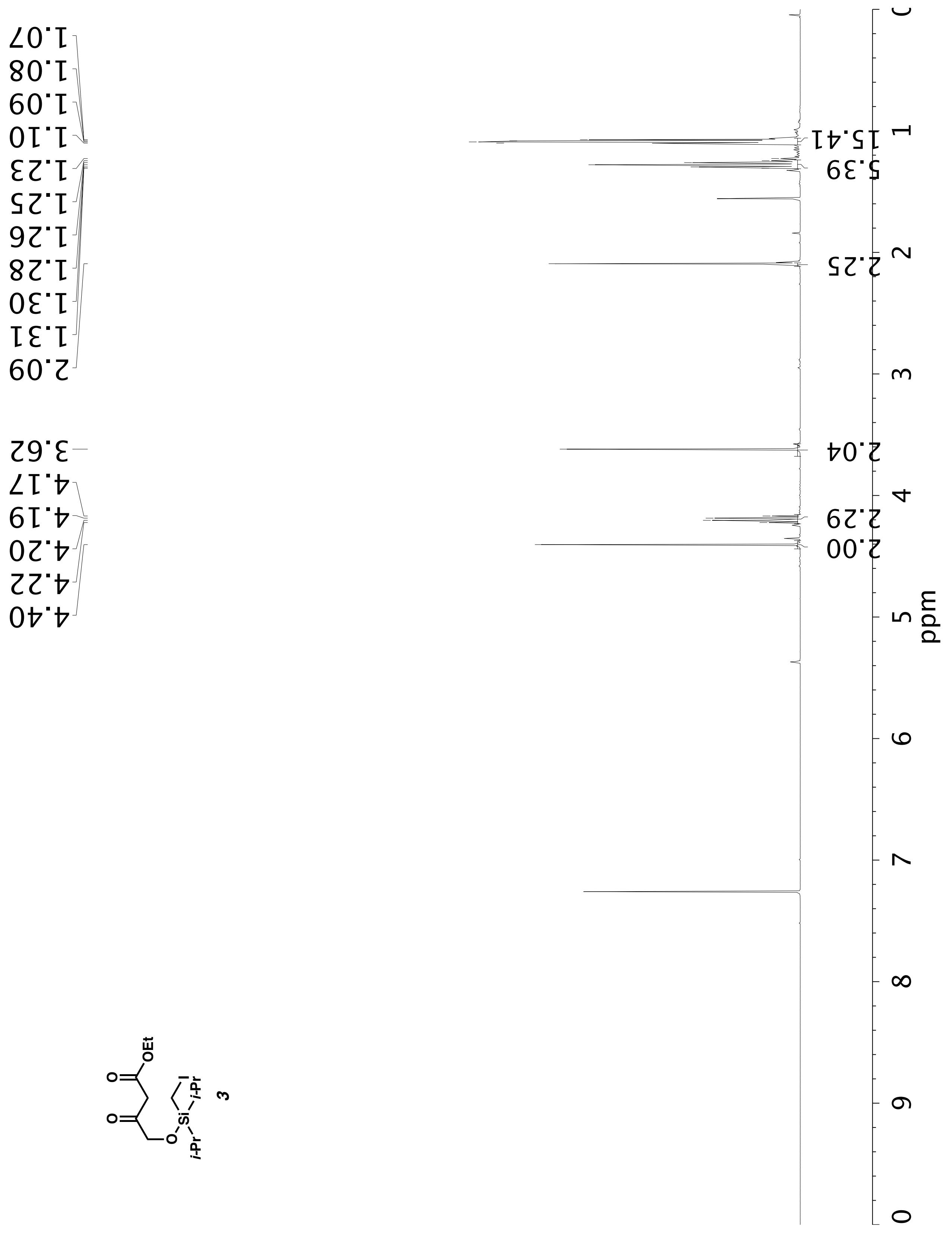

^{1}H NMR (400 MHz, $CDCl_3$) of compound **3**.

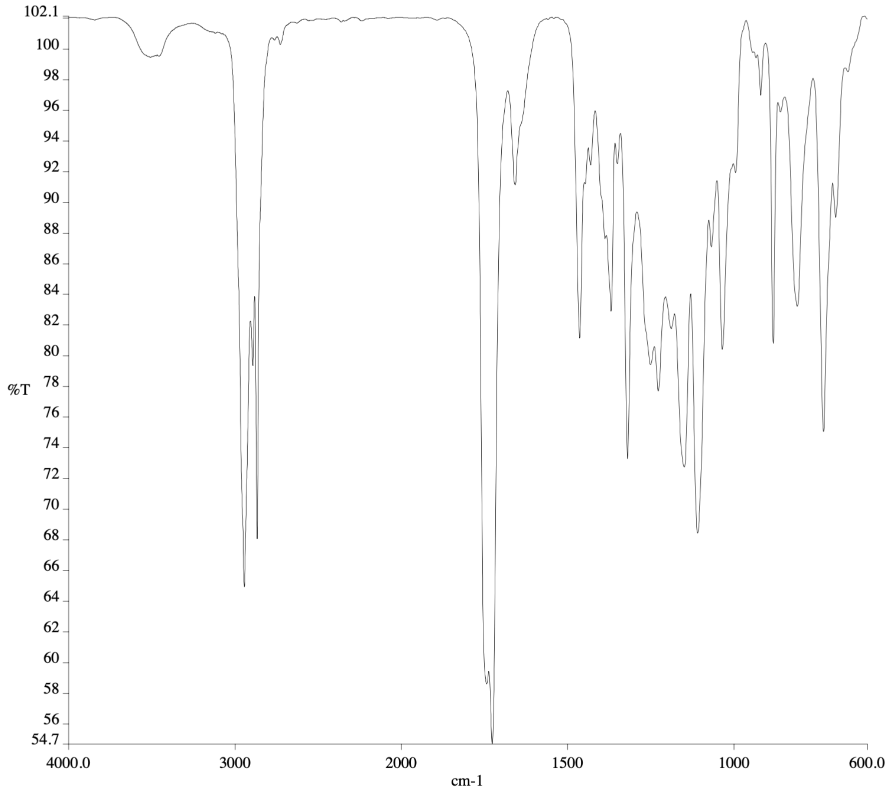

Infrared spectrum (Thin Film, NaCl) of compound **3**.

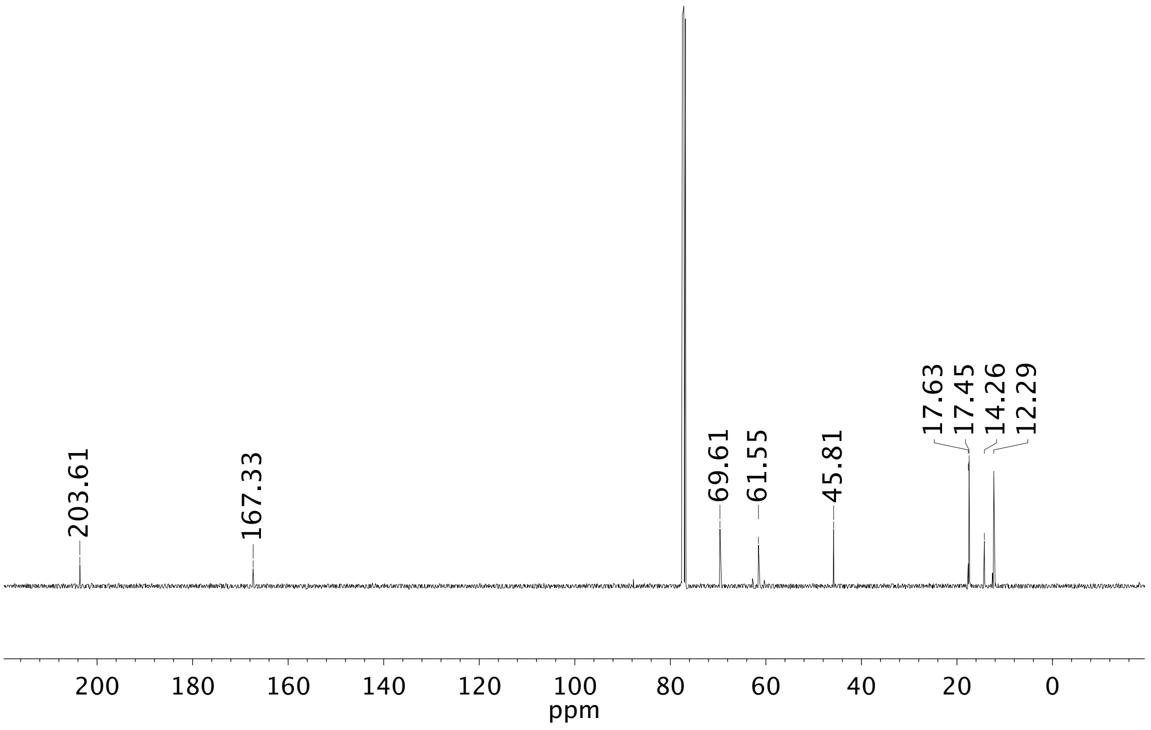

^{13}C NMR (100 MHz, $CDCl_3$) of compound **3**.

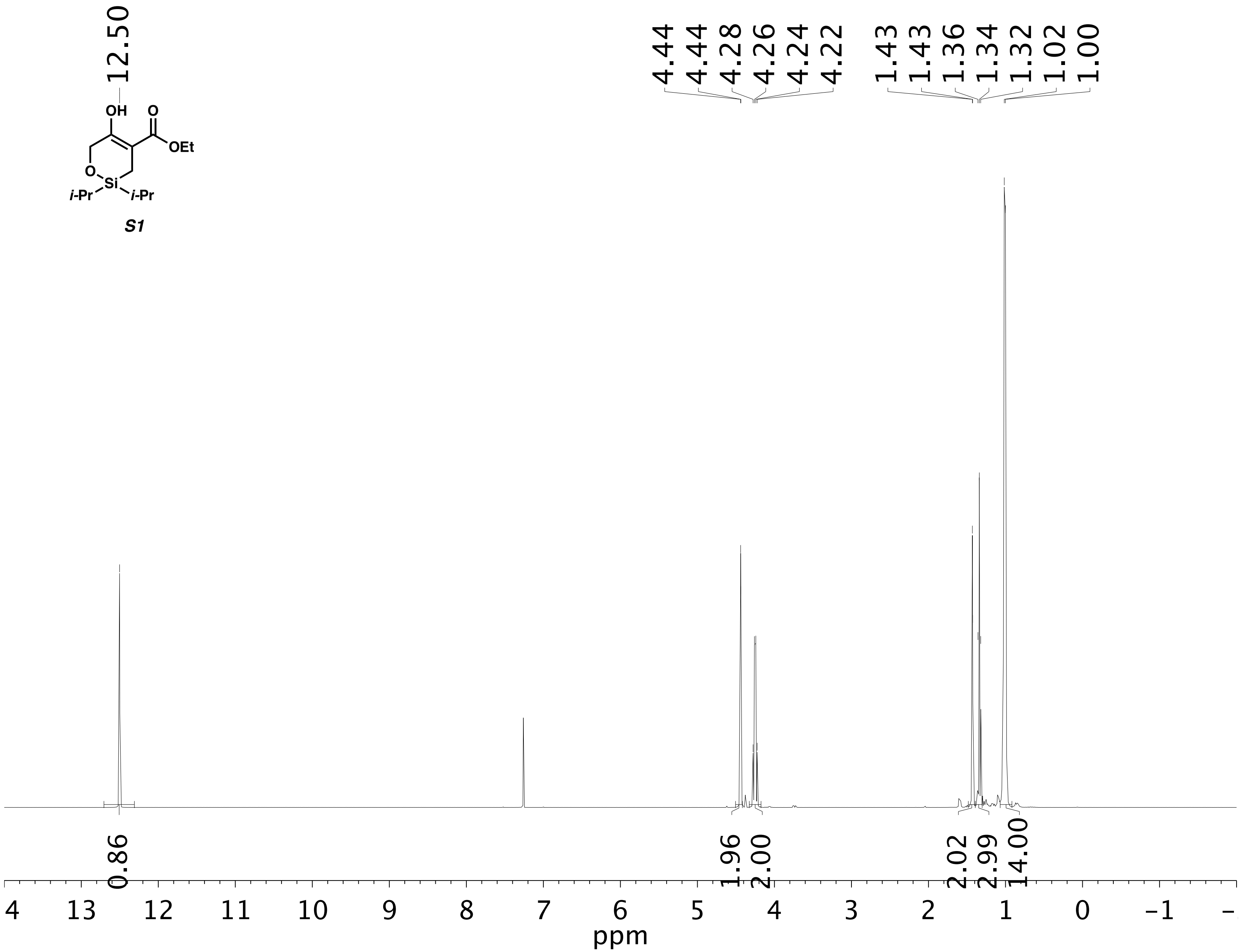

^{1}H NMR (400 MHz, $CDCl_3$) of compound **S1**.

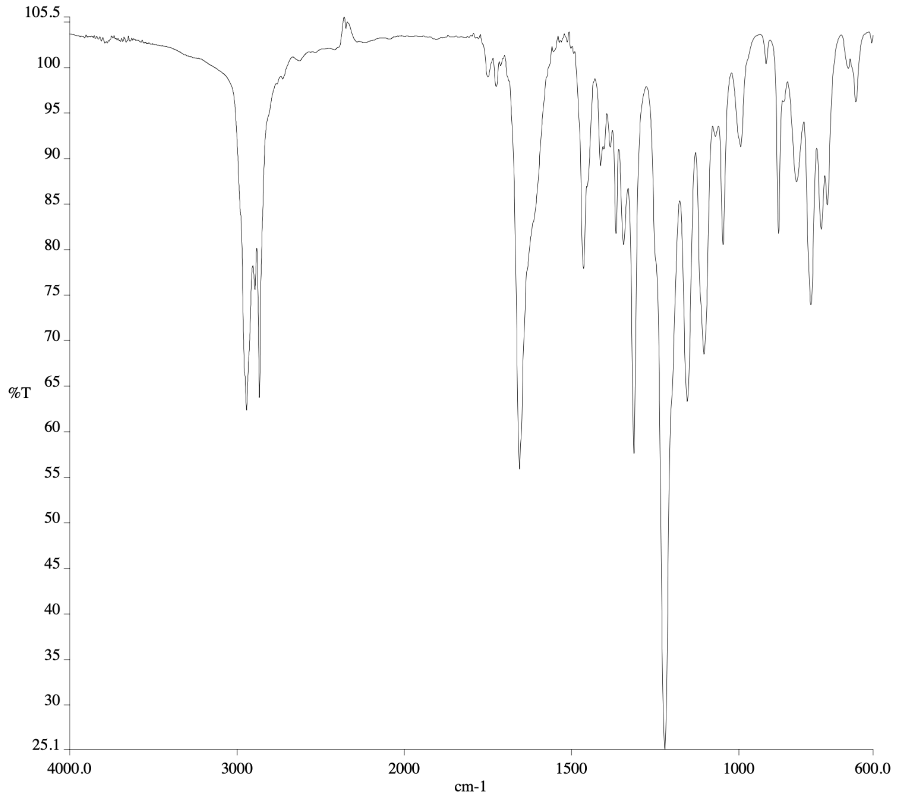

Infrared spectrum (Thin Film, NaCl) of compound **S1**.

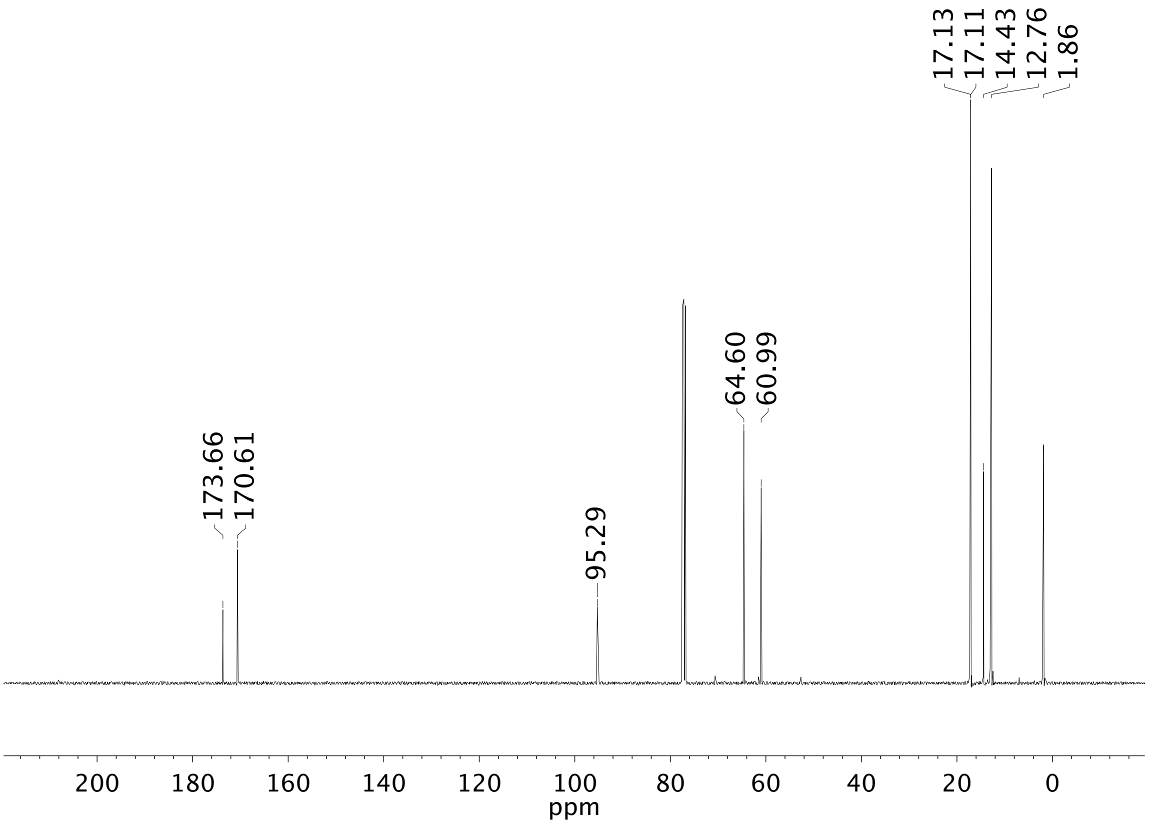

^{13}C NMR (126 MHz, $CDCl_3$) of compound **S1**.

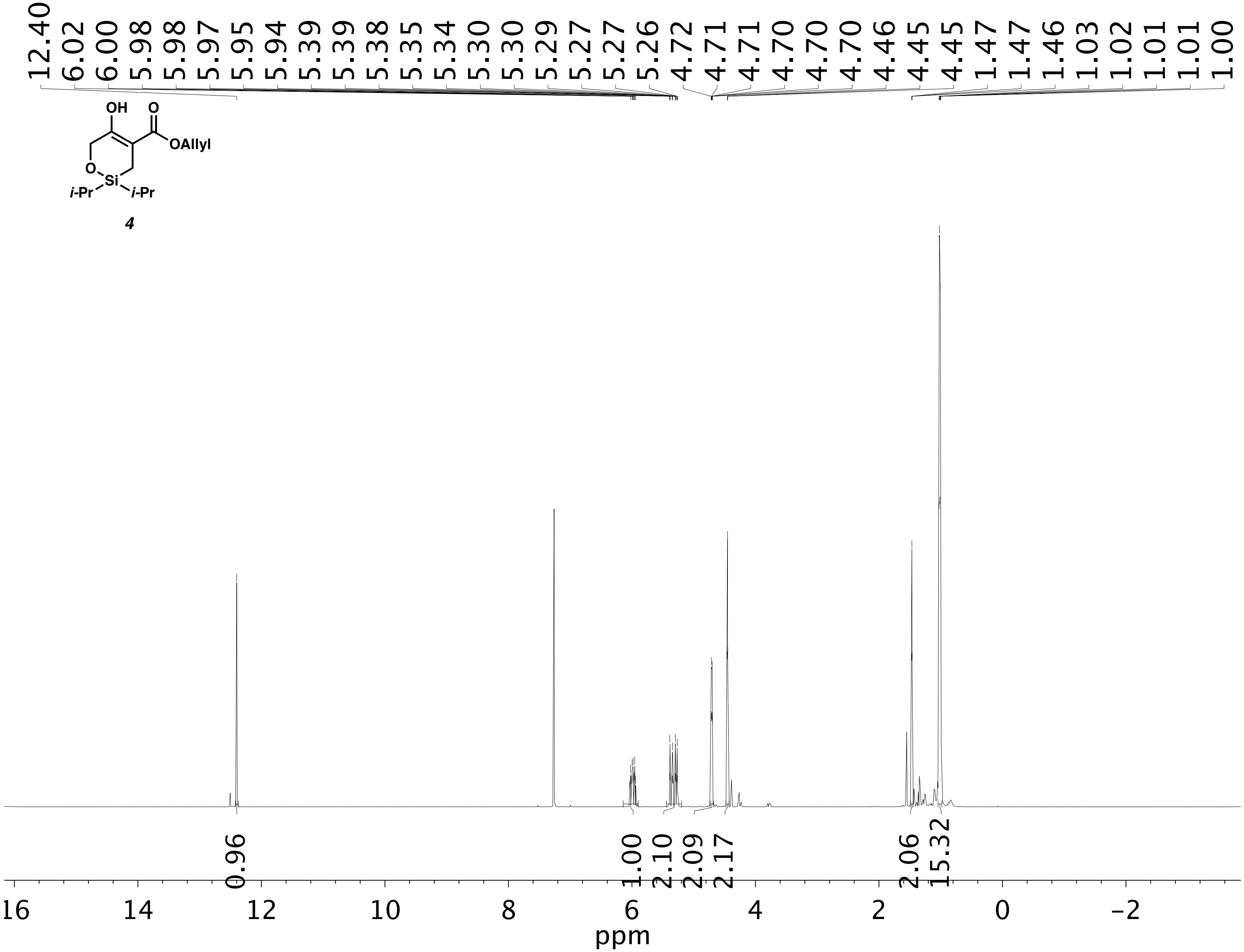

^{1}H NMR (400 MHz, $CDCl_3$) of compound **4**.

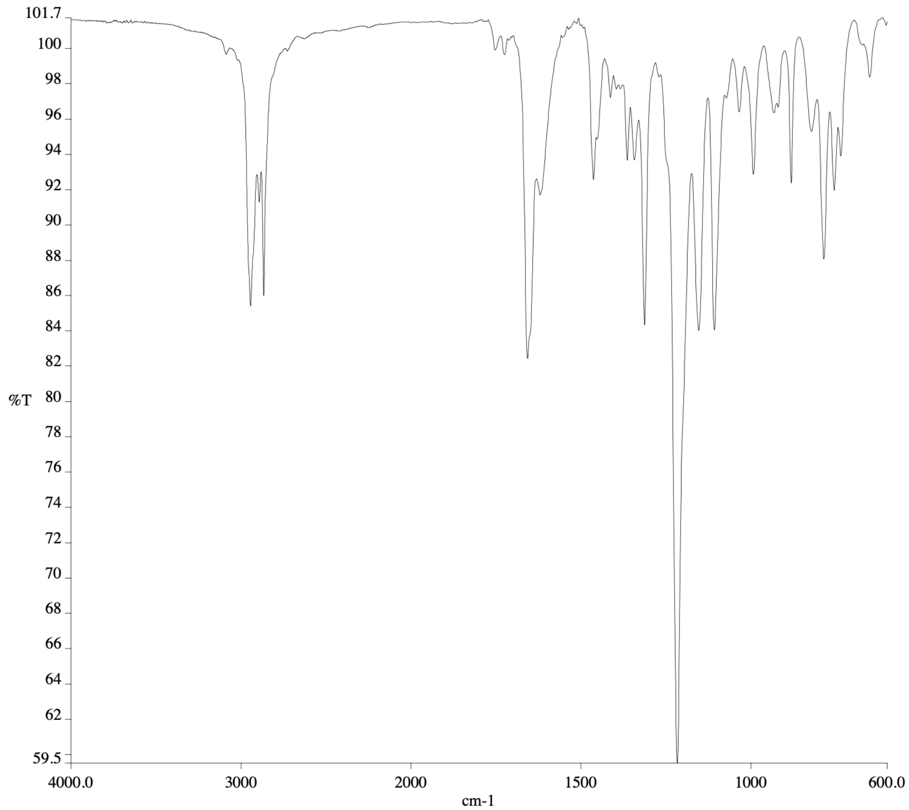

Infrared spectrum (Thin Film, NaCl) of compound **4**.

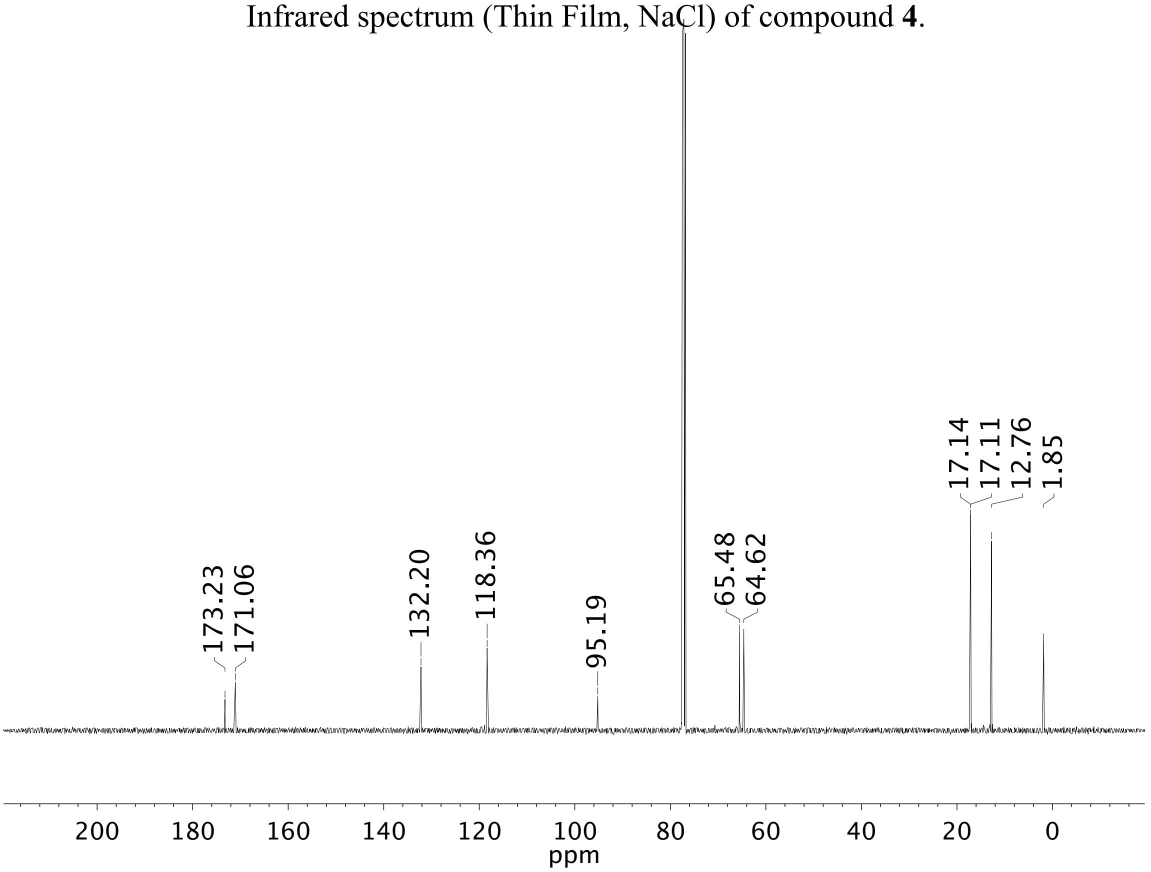

^{13}C NMR (100 MHz, $CDCl_3$) of compound **4**.

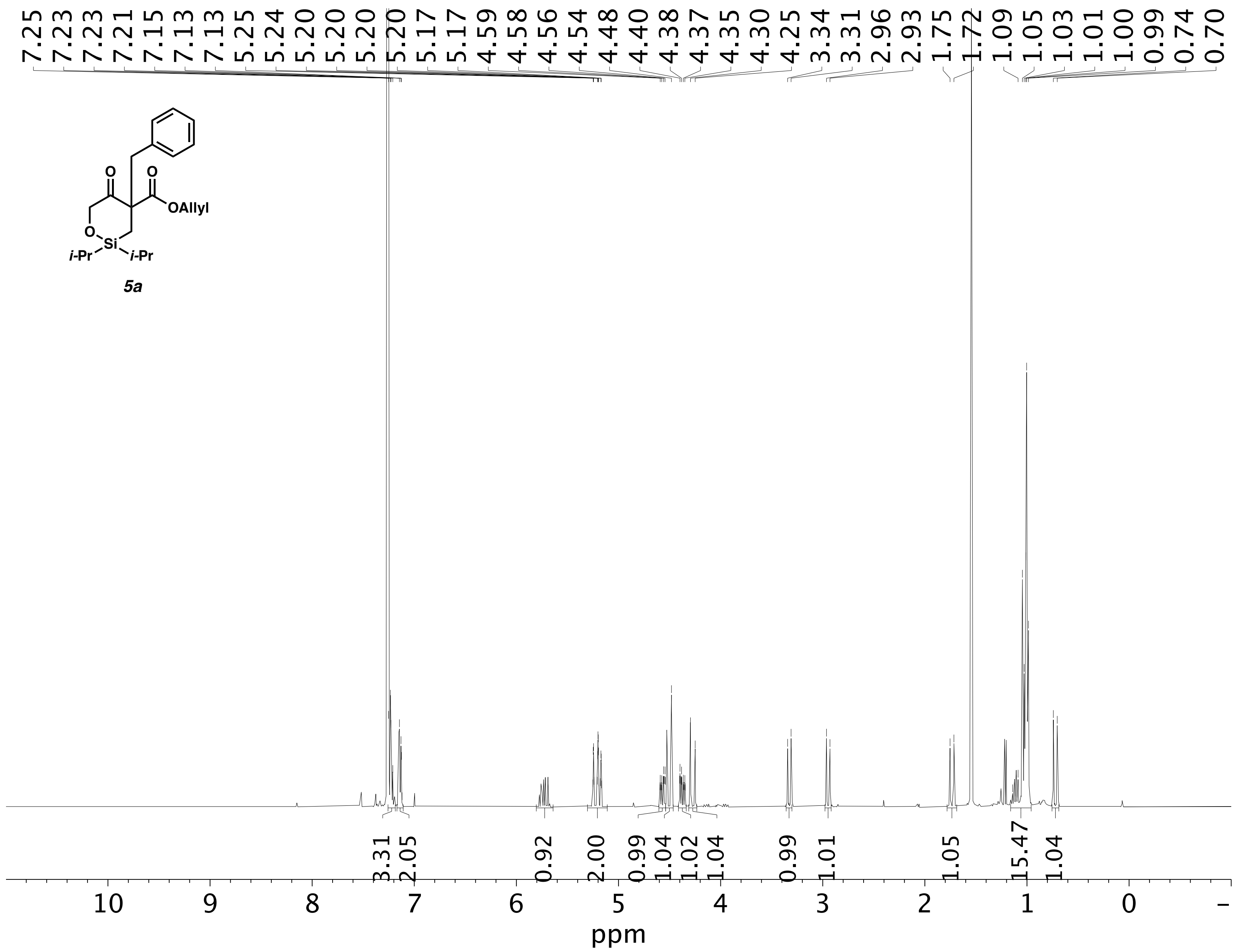

^{1}H NMR (500 MHz, $CDCl_3$) of compound **5a**.

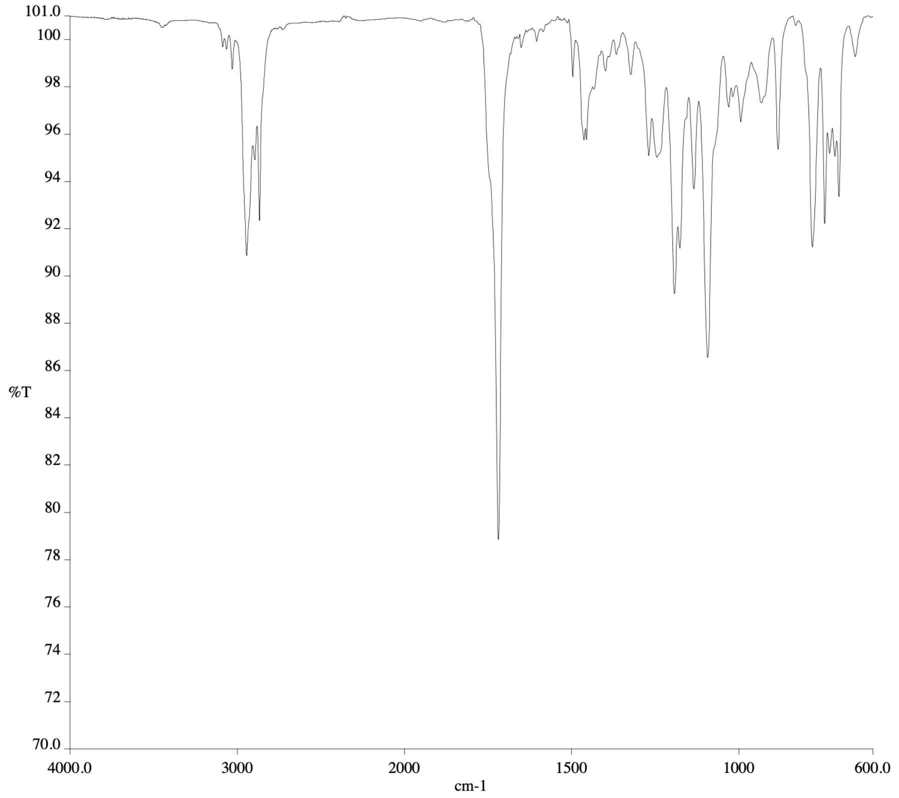

Infrared spectrum (Thin Film, NaCl) of compound **5a**.

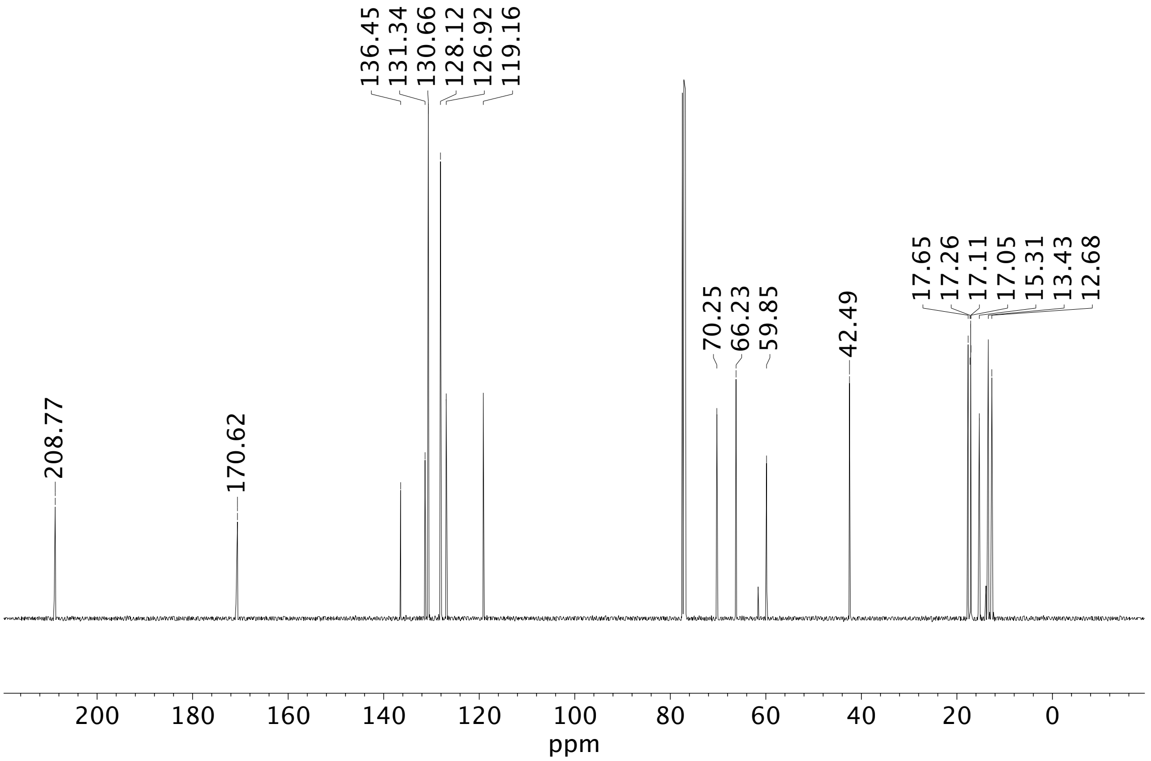

^{13}C NMR (100 MHz, $CDCl_3$) of compound **5a**.

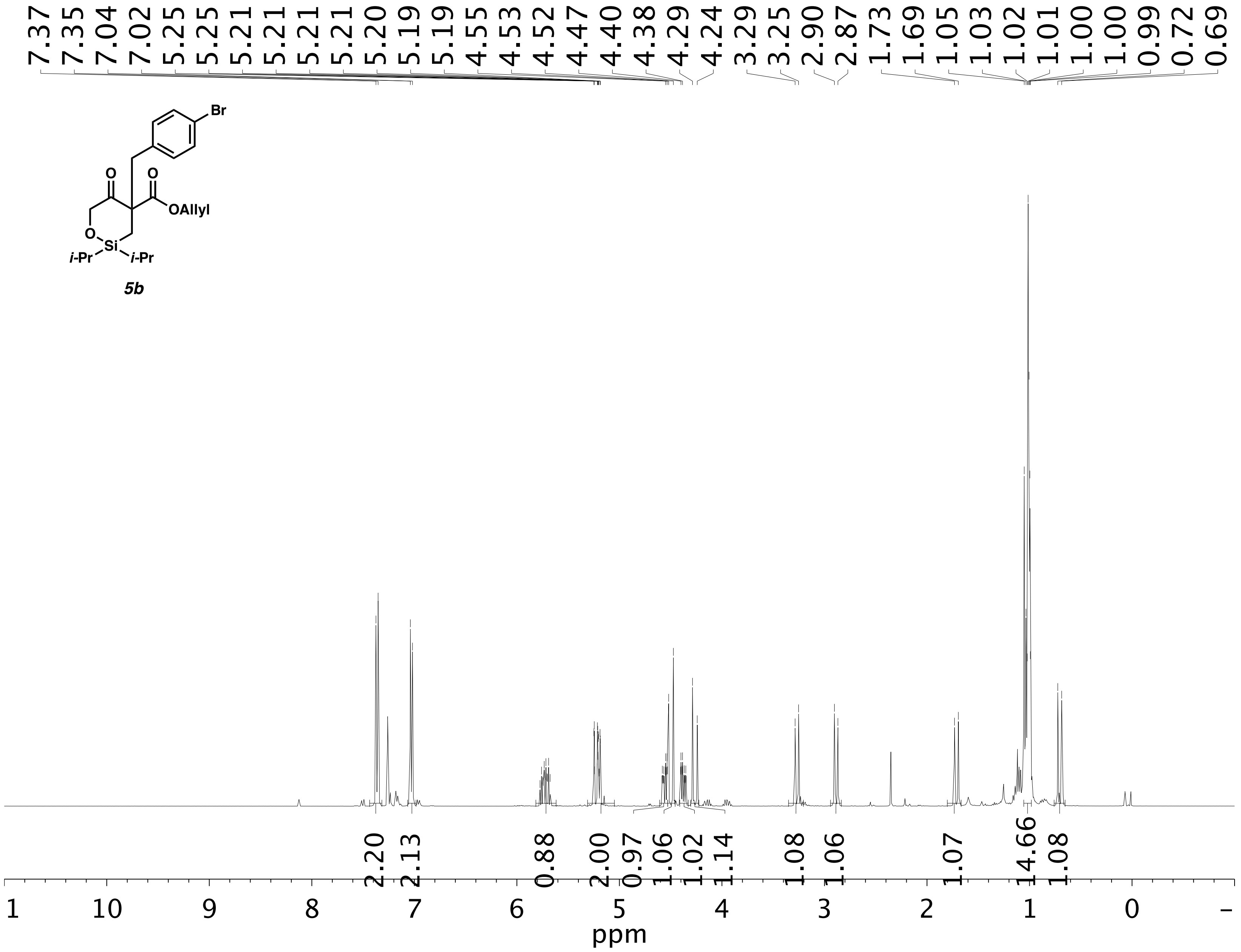

^{1}H NMR (400 MHz, $CDCl_3$) of compound **5b**.

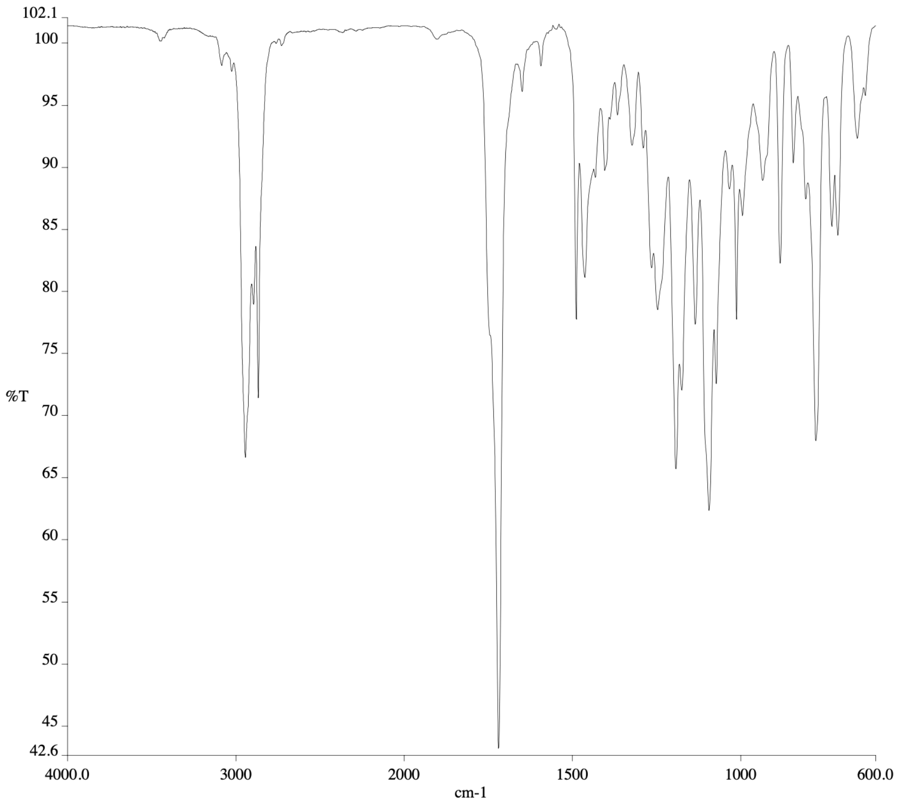

Infrared spectrum (Thin Film, NaCl) of compound **5b**.

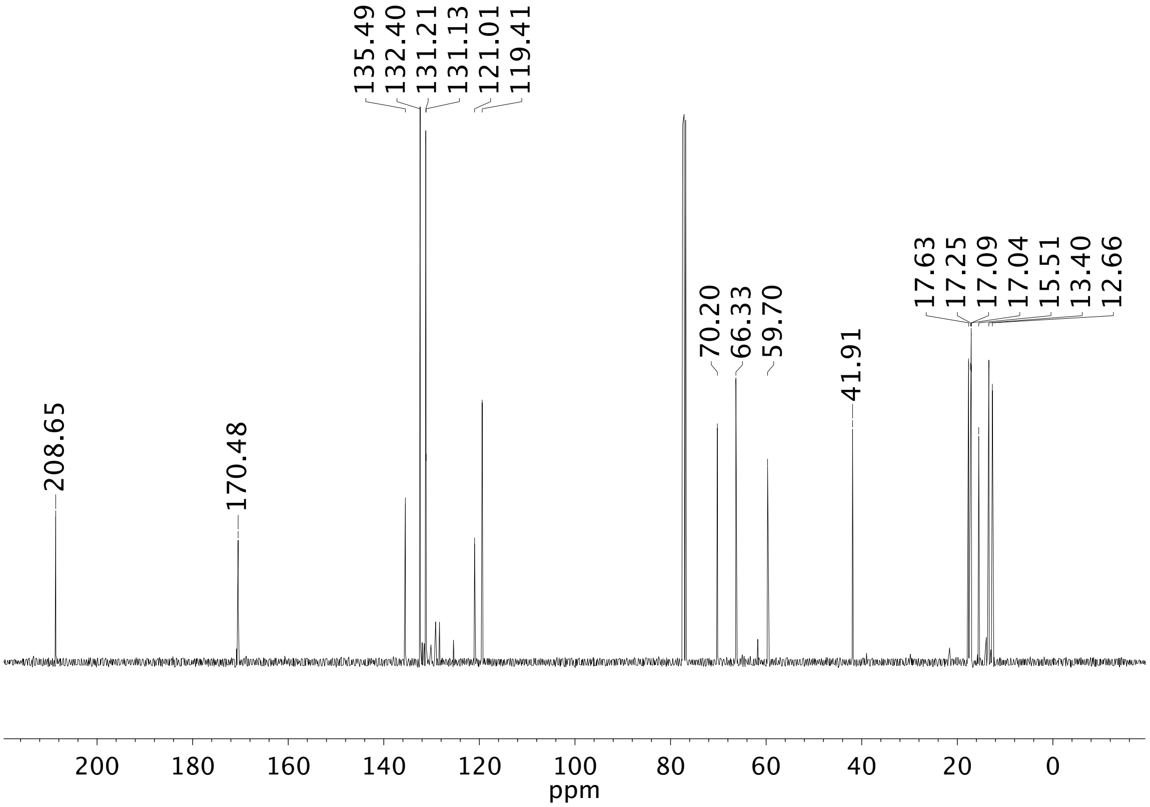

^{13}C NMR (100 MHz, $CDCl_3$) of compound **5b**.

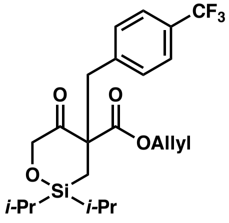

5c

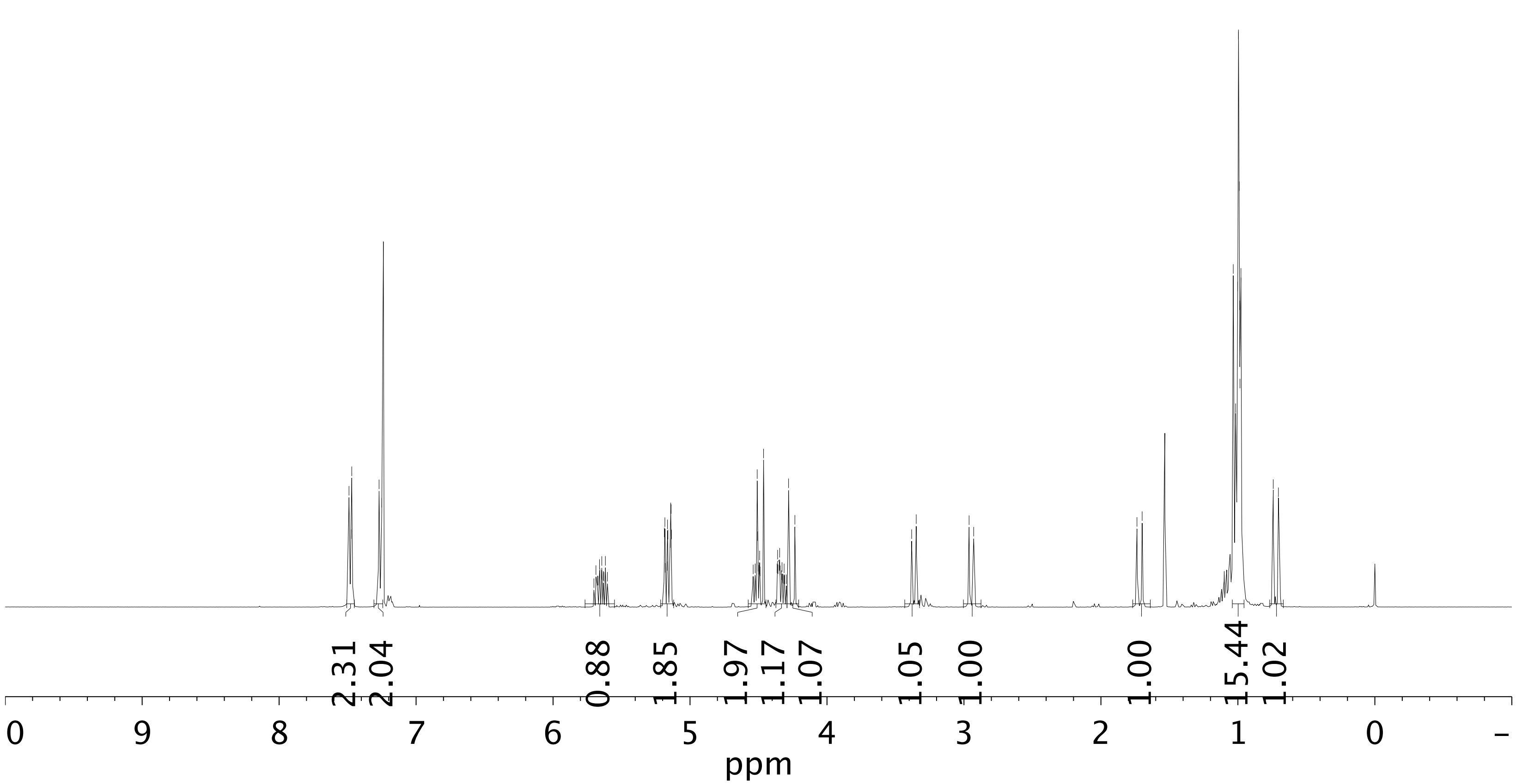

^{1}H NMR (500 MHz, $CDCl_3$) of compound **5c**.

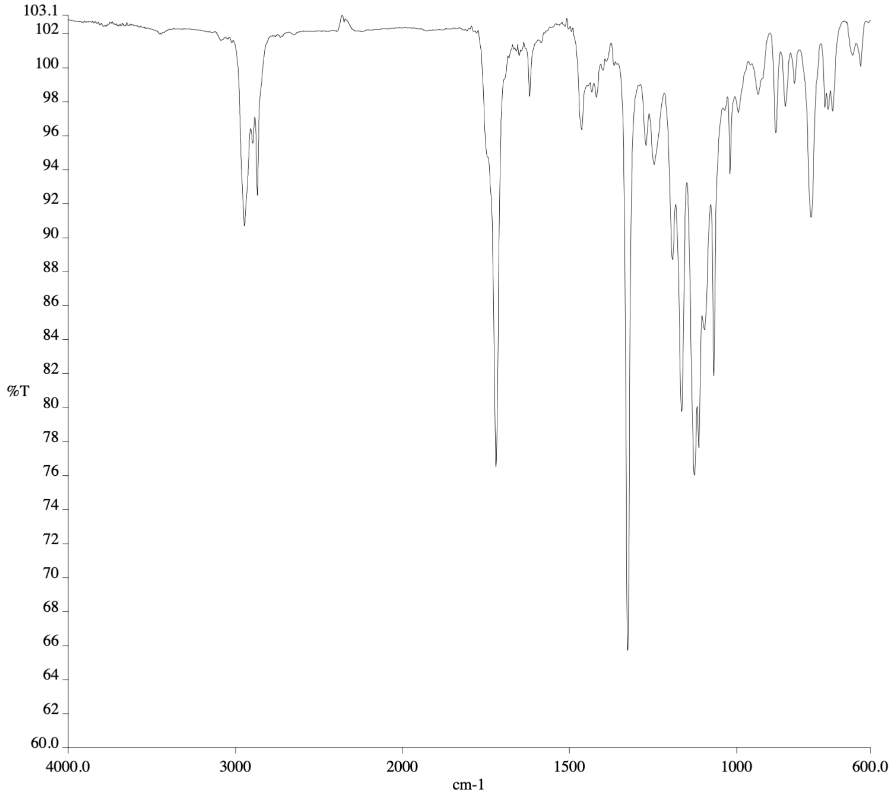

Infrared spectrum (Thin Film, NaCl) of compound **5c**.

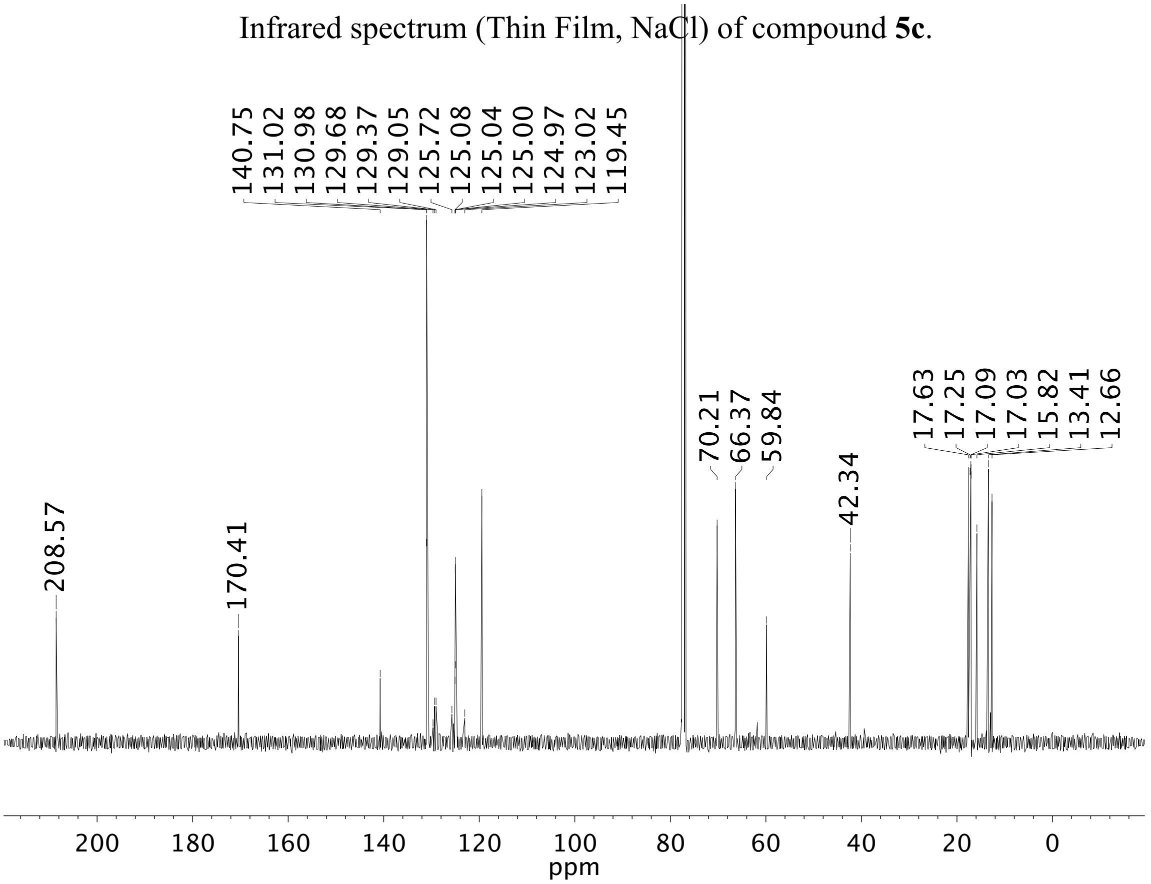

^{13}C NMR (100 MHz, $CDCl_3$) of compound **5c**.

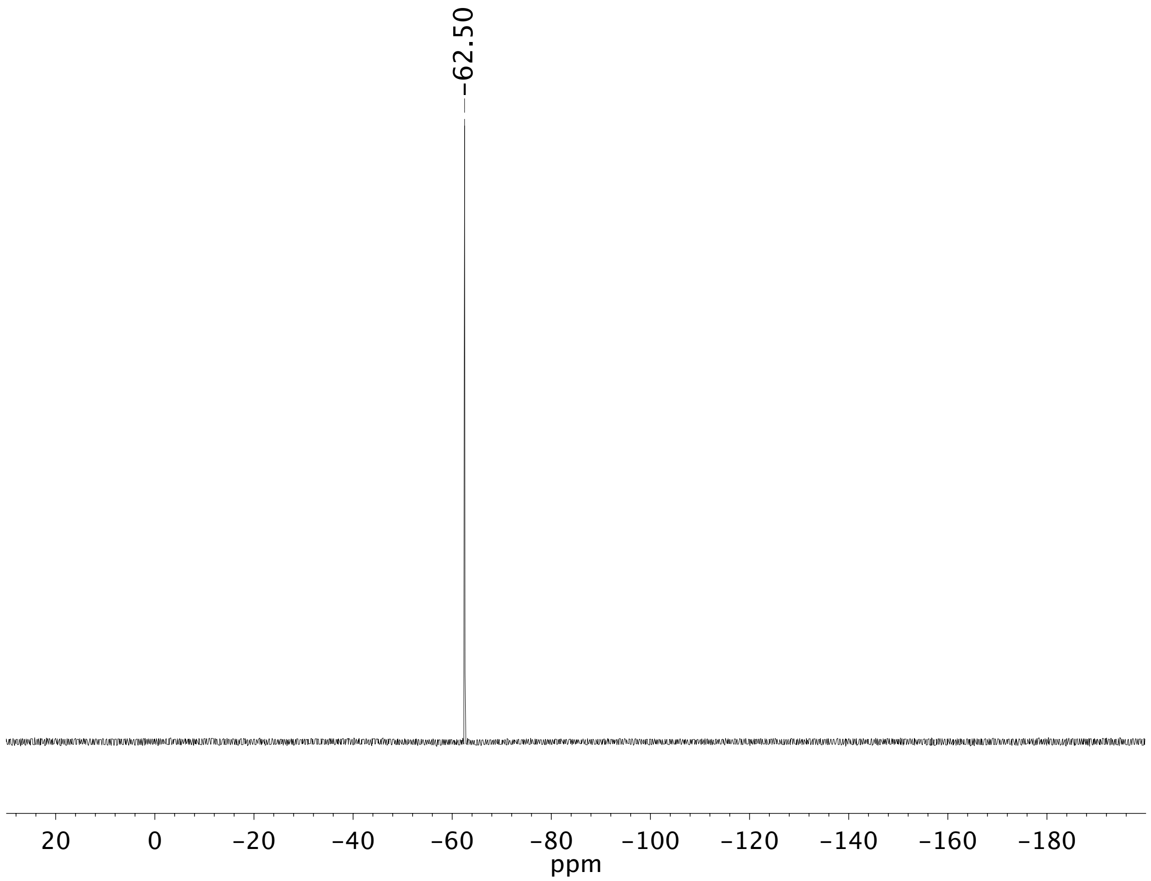

^{19}F NMR (282 MHz, $CDCl_3$) of compound **5c**.

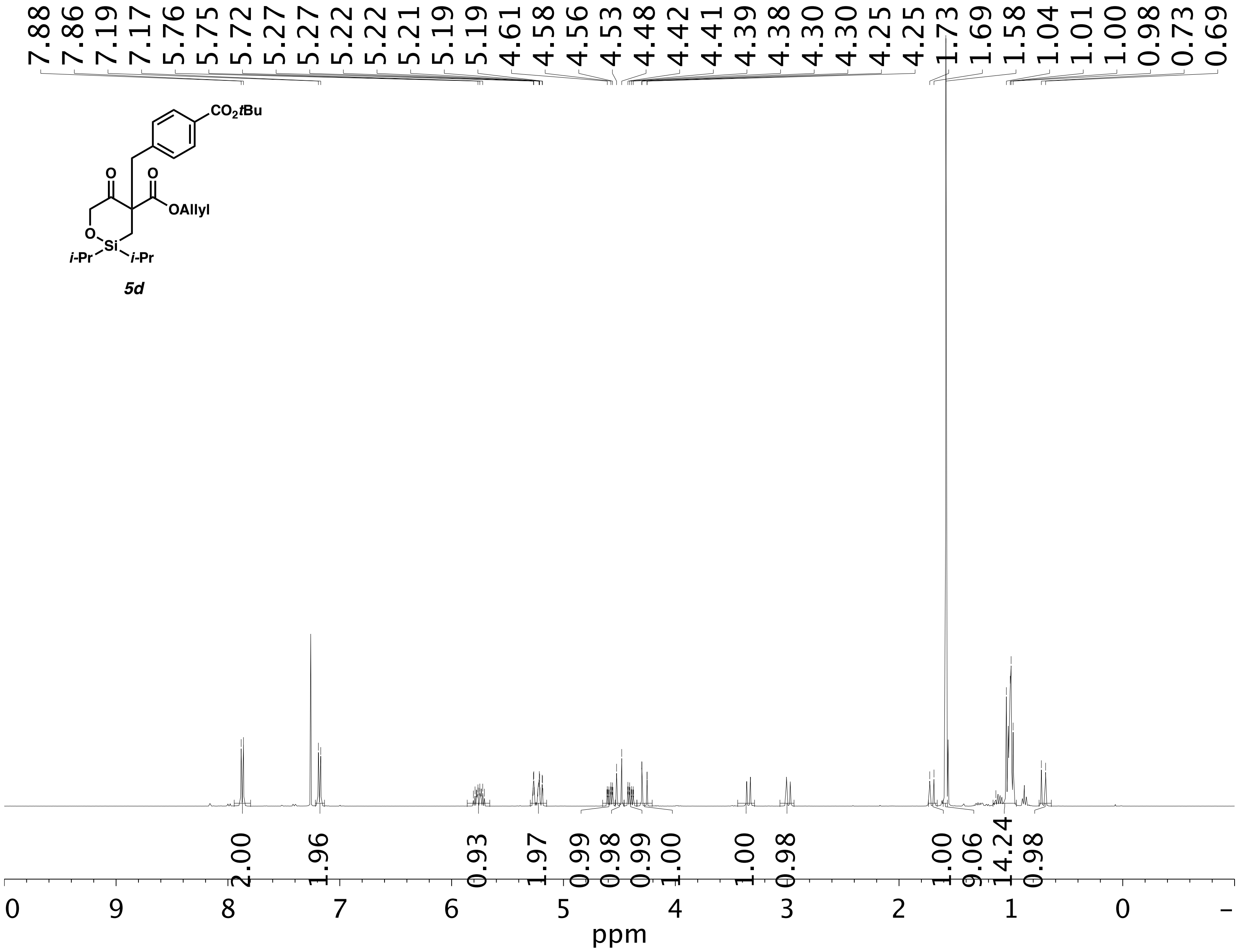

^{1}H NMR (500 MHz, $CDCl_3$) of compound **5d**.

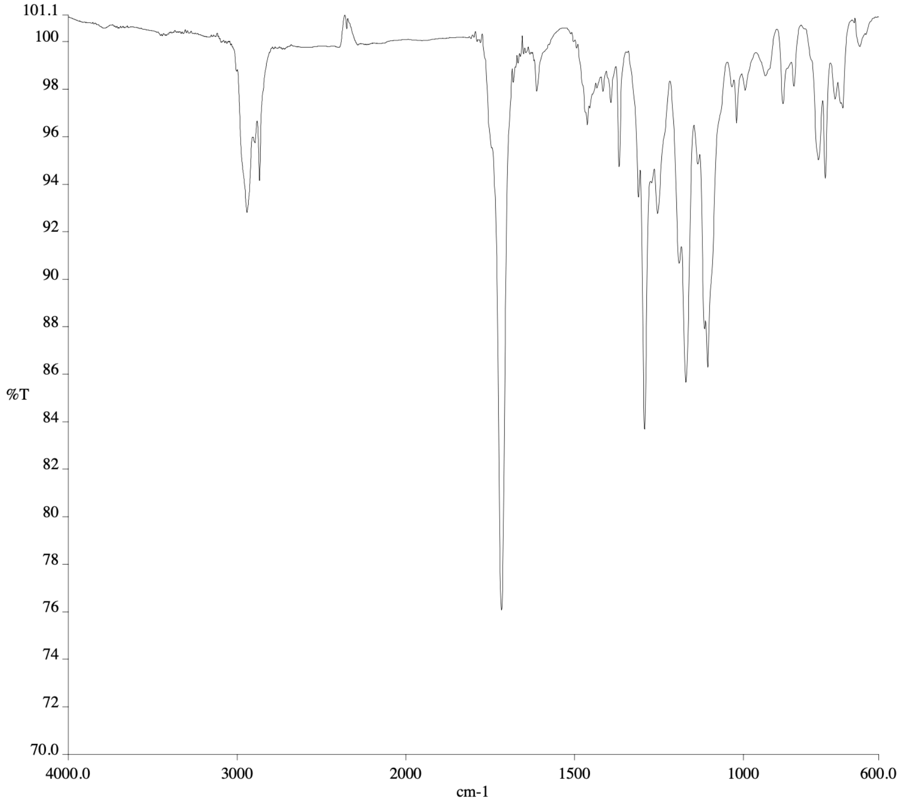

Infrared spectrum (Thin Film, NaCl) of compound **5d**.

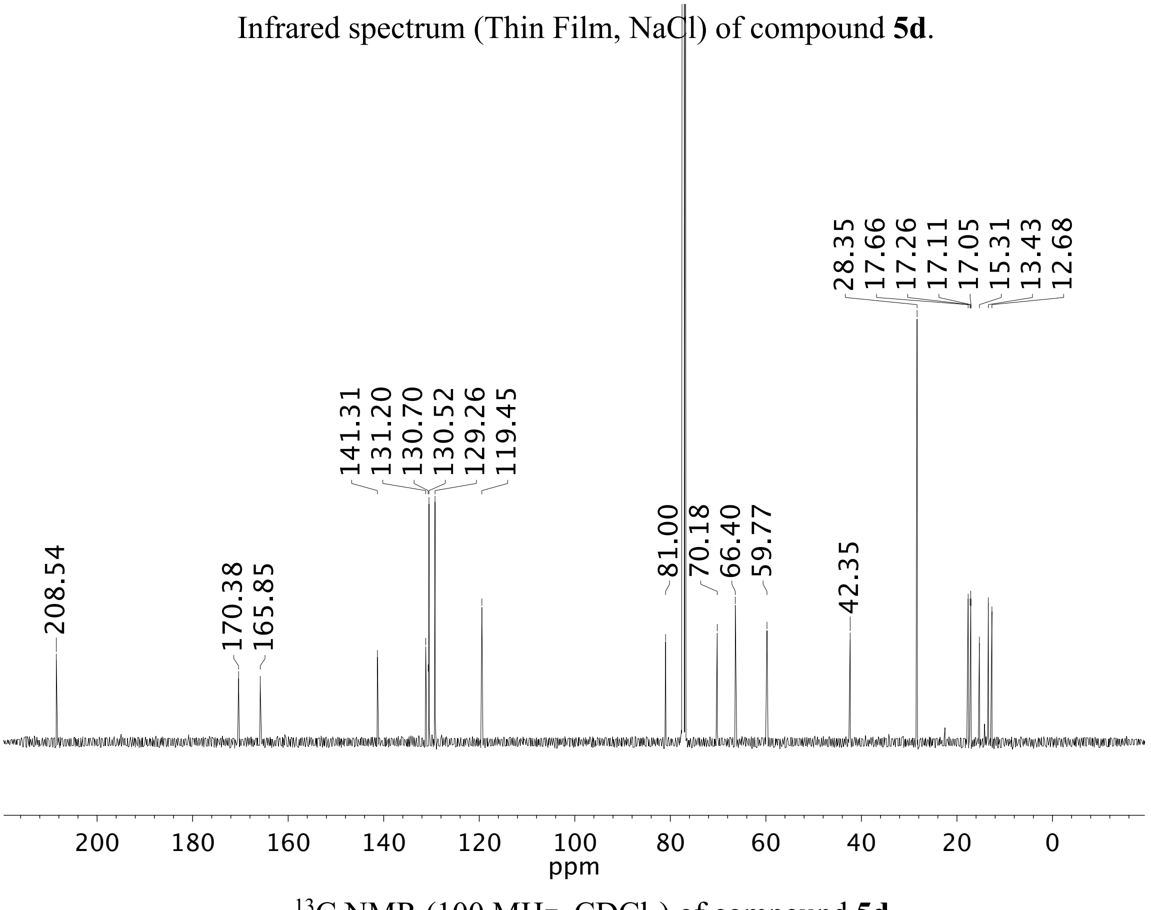

^{13}C NMR (100 MHz, $CDCl_3$) of compound **5d**.

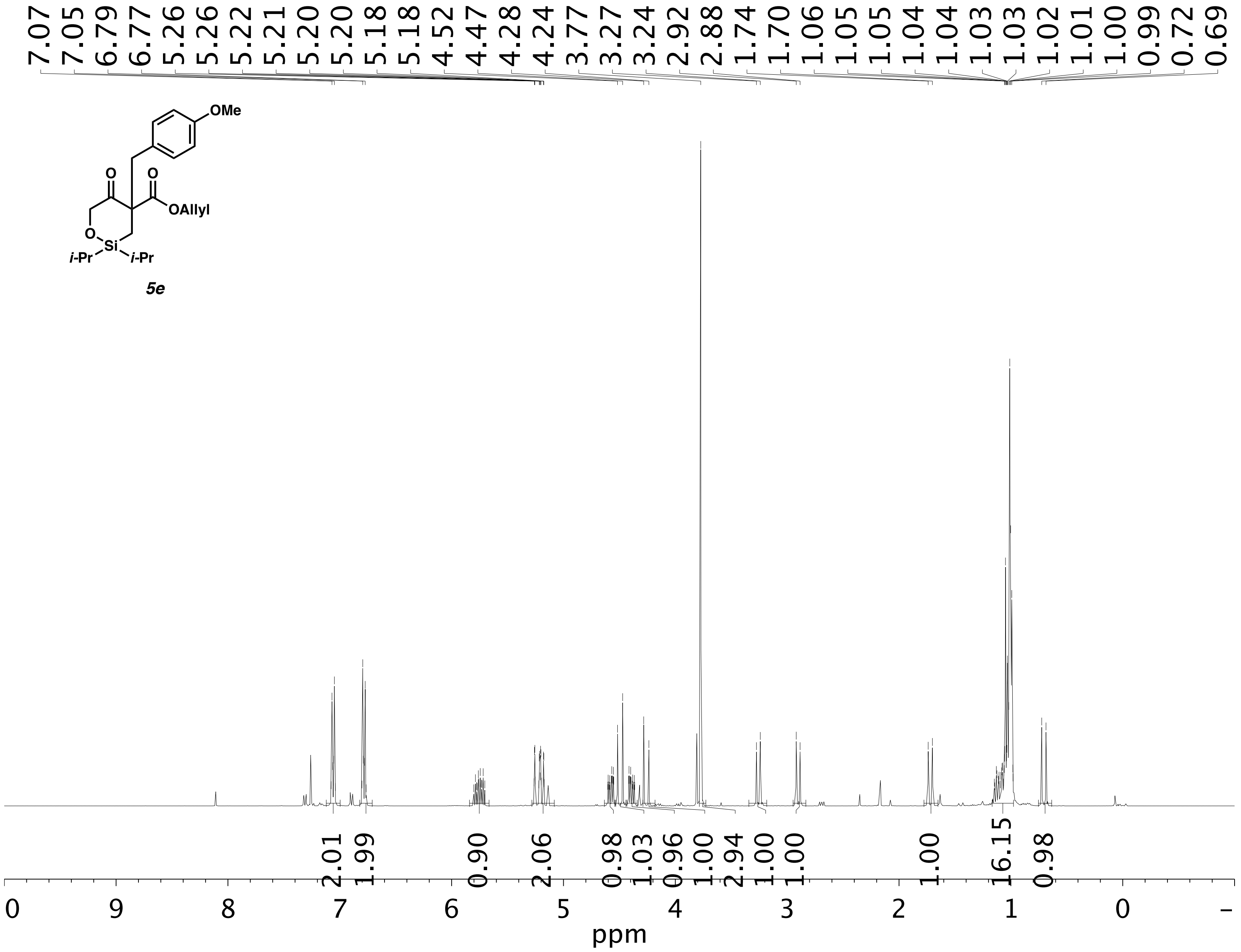

^{1}H NMR (400 MHz, $CDCl_3$) of compound **5e**.

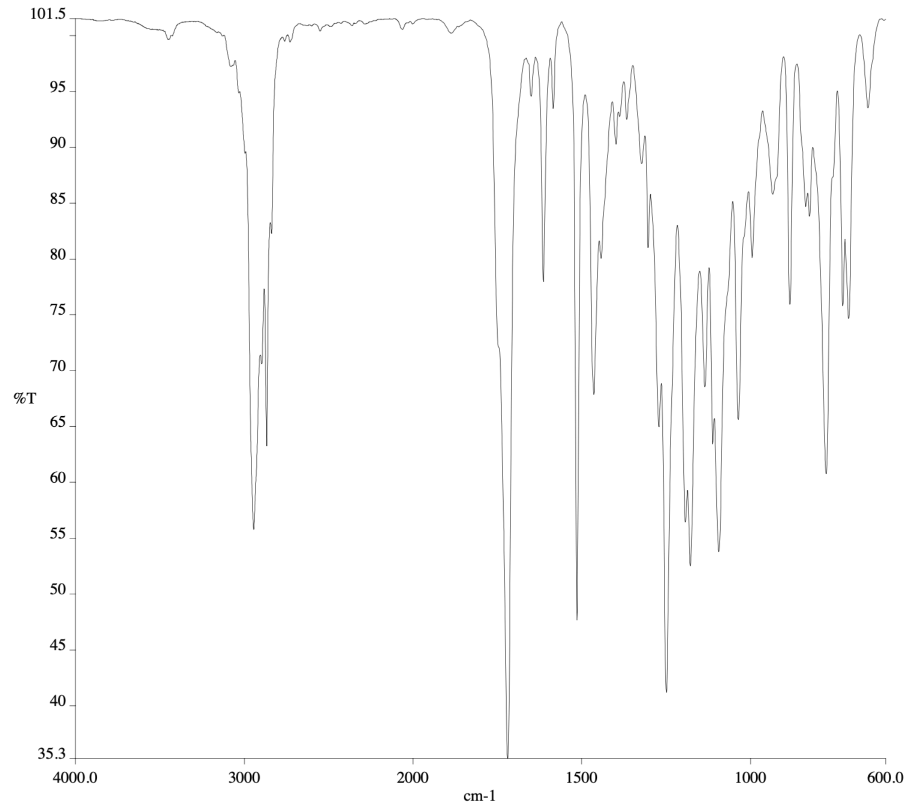

Infrared spectrum (Thin Film, NaCl) of compound **5e**.

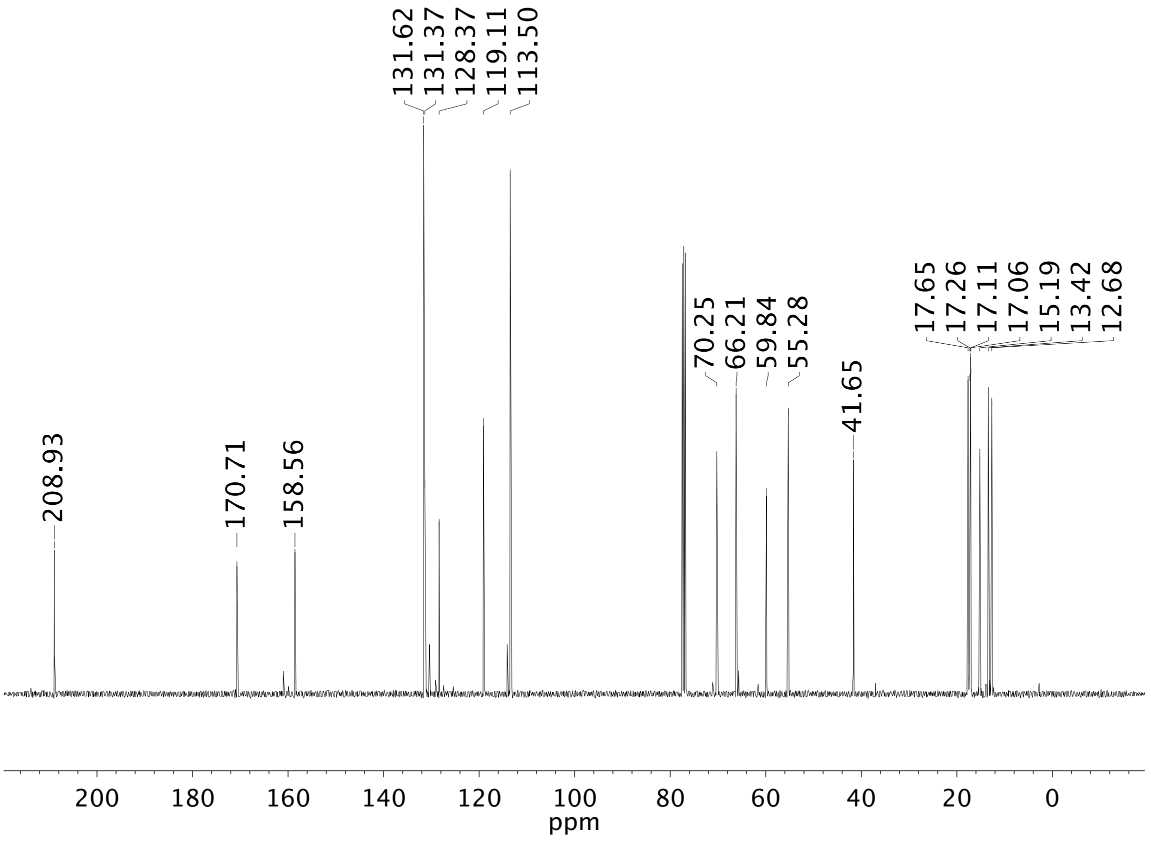

^{13}C NMR (100 MHz, $CDCl_3$) of compound **5e**.

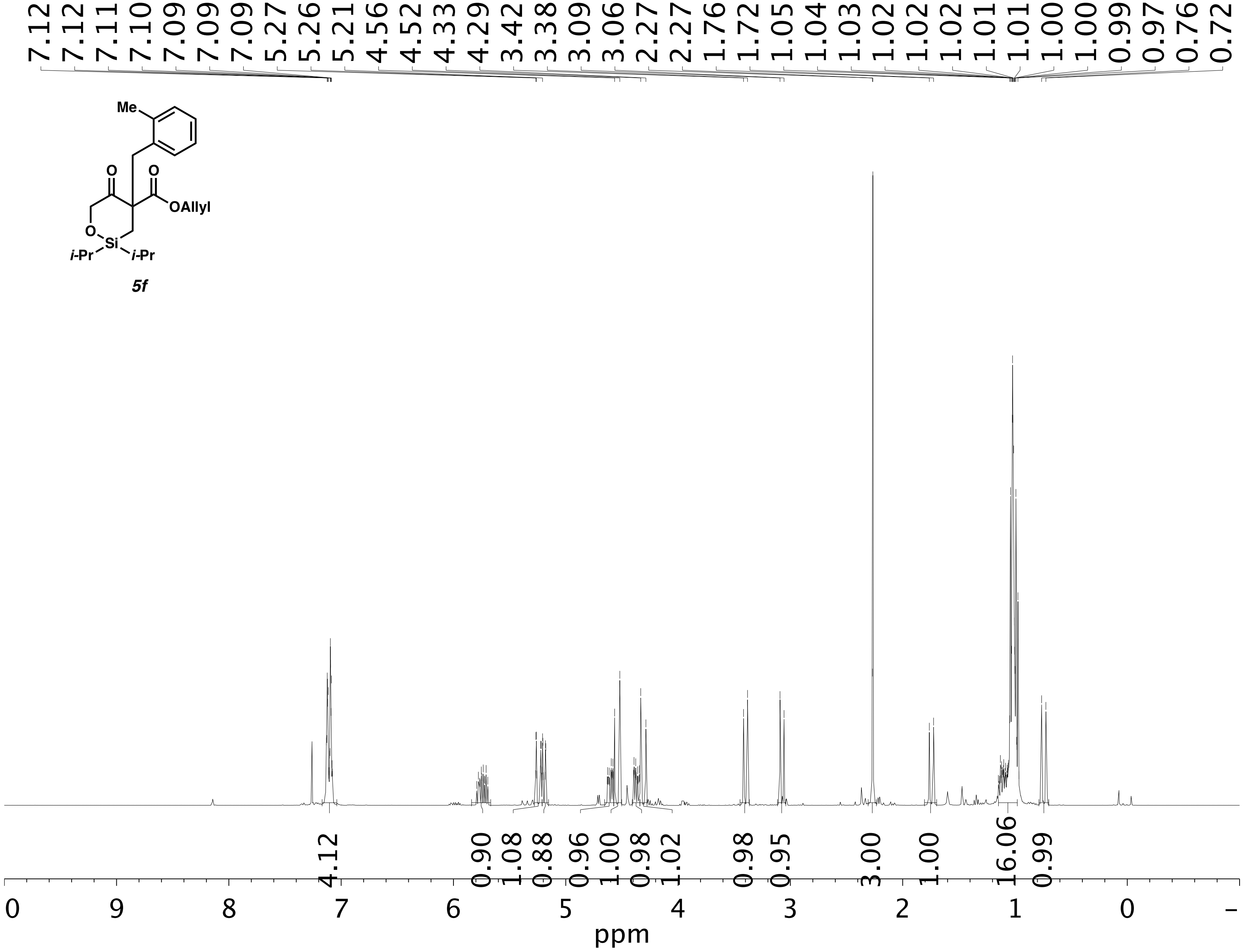

^{1}H NMR (400 MHz, $CDCl_3$) of compound **5f**.

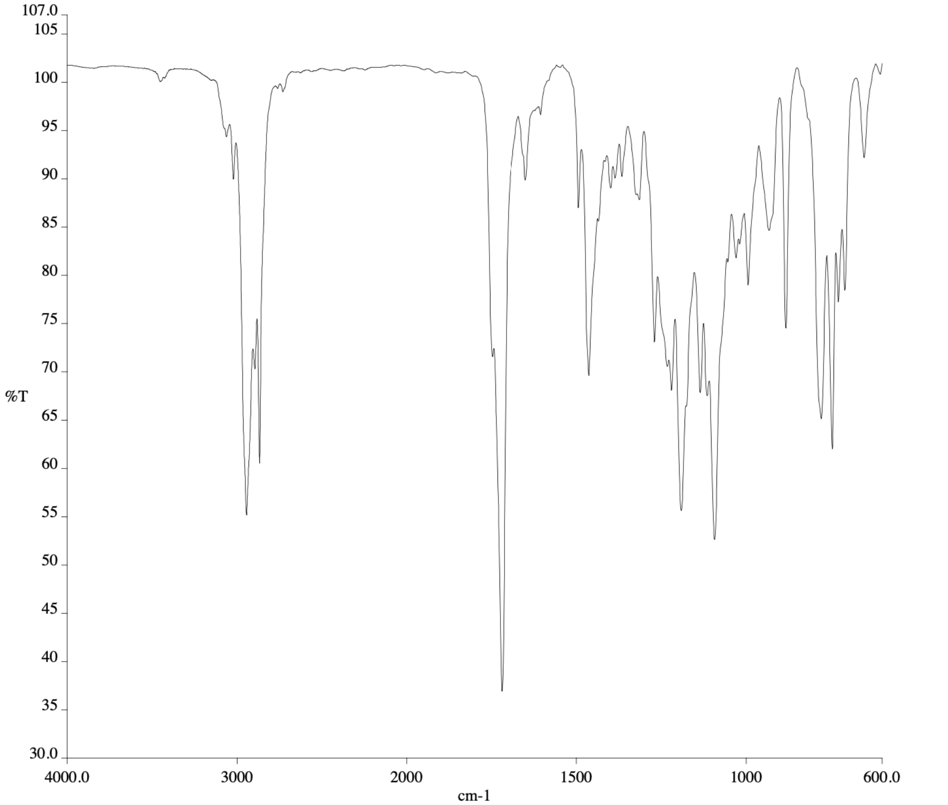

Infrared spectrum (Thin Film, NaCl) of compound **5f**.

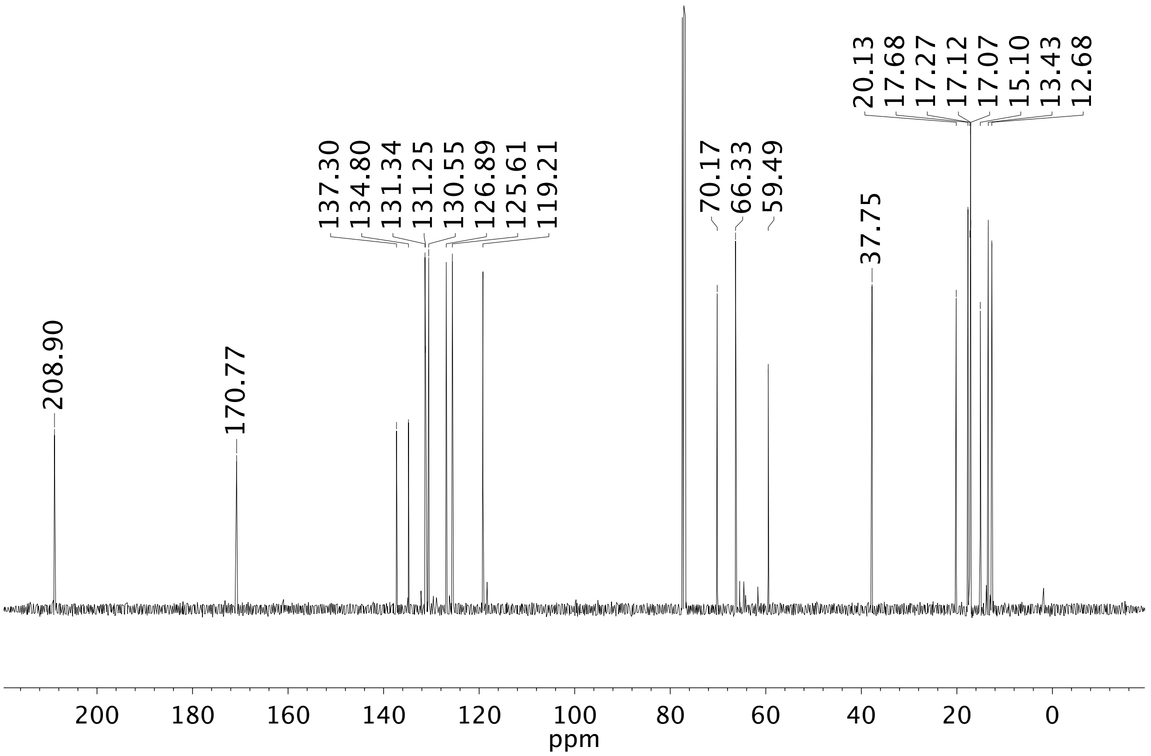

^{13}C NMR (100 MHz, $CDCl_3$) of compound **5f**.

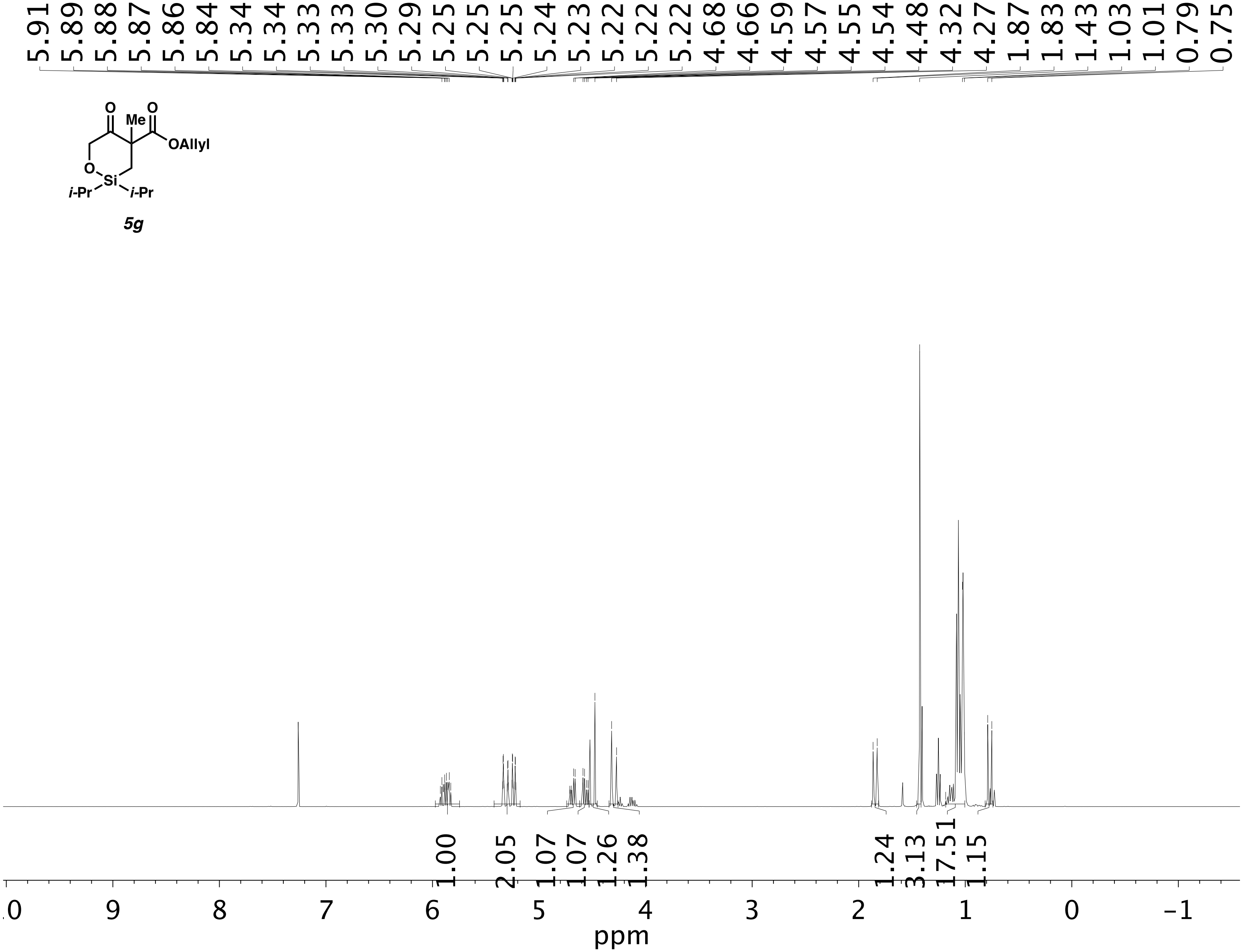

^1H NMR (400 MHz, $CDCl_3$) of compound **5g**.

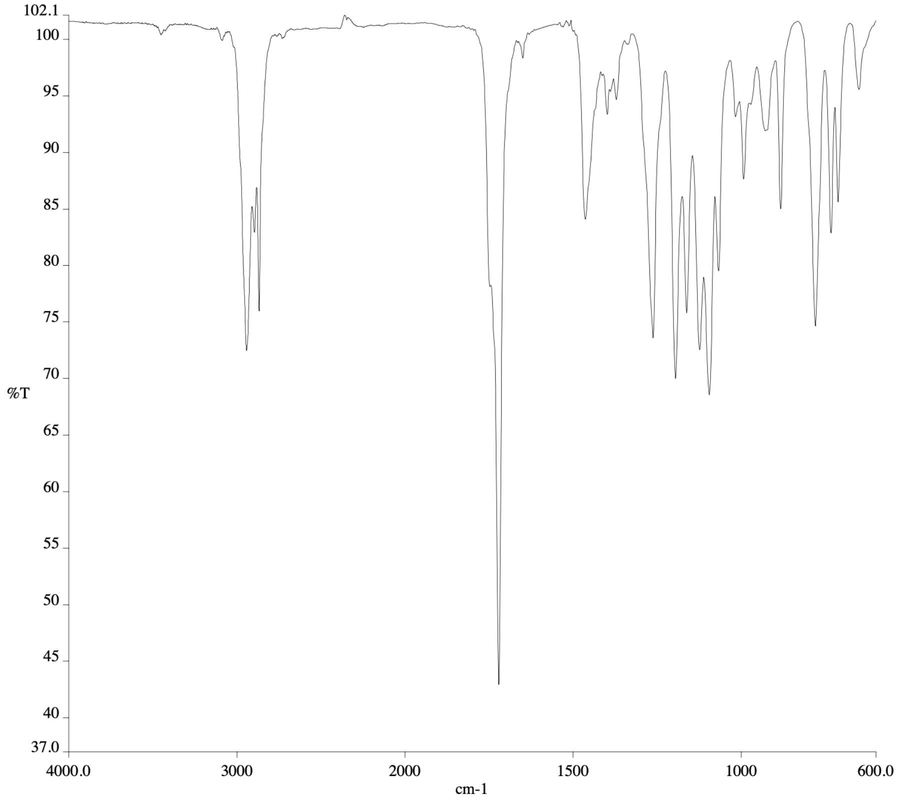

Infrared spectrum (Thin Film, NaCl) of compound **5g**.

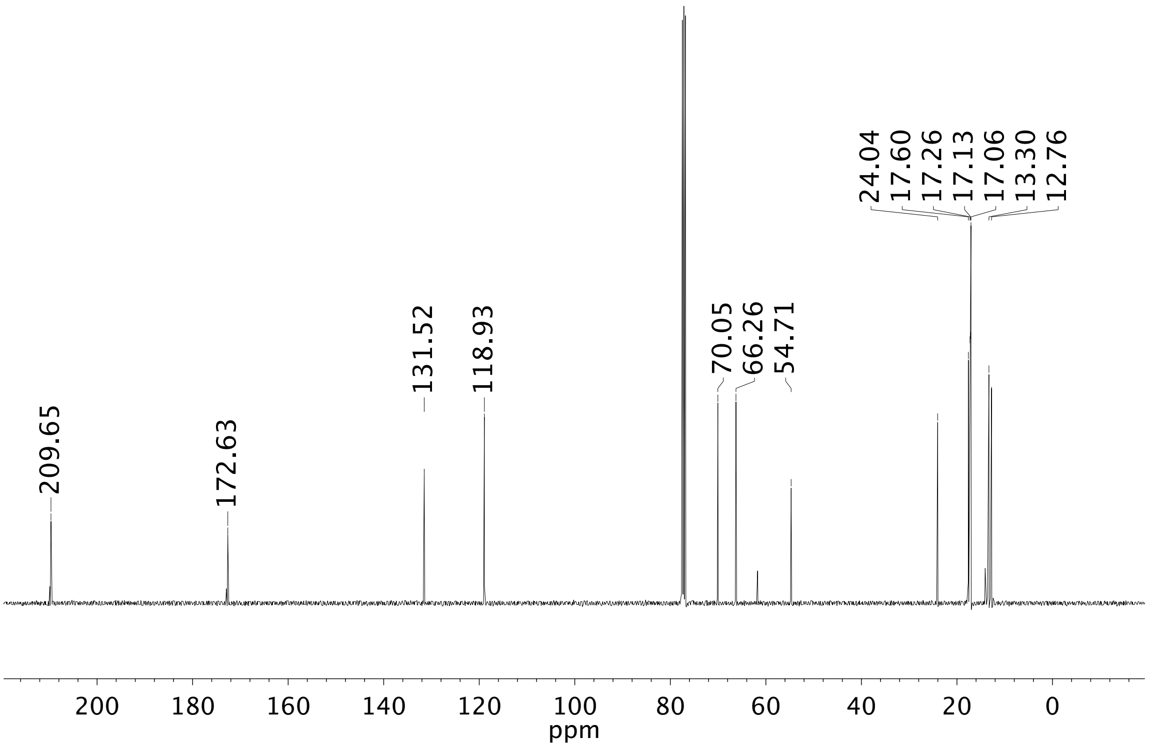

^{13}C NMR (100 MHz, $CDCl_3$) of compound **5g**.

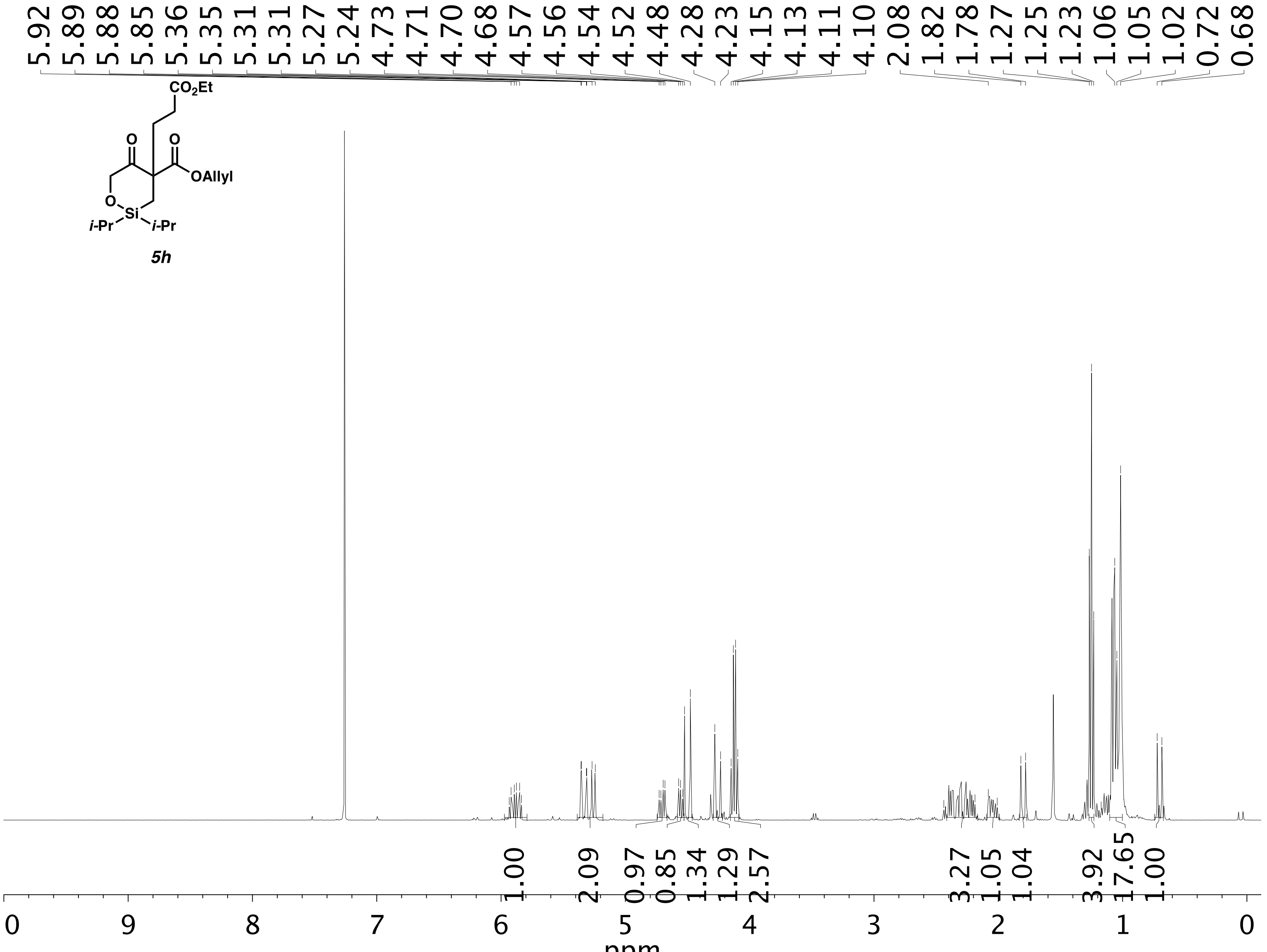

^{1}H NMR (400 MHz, $CDCl_3$) of compound **5h**.

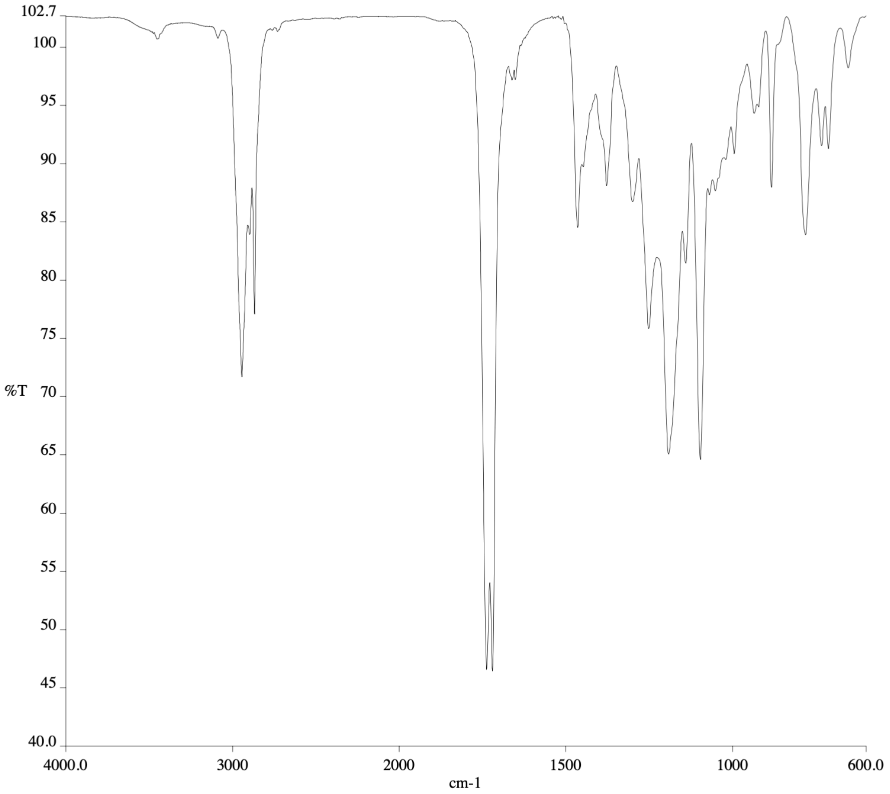

Infrared spectrum (Thin Film, NaCl) of compound **5h**.

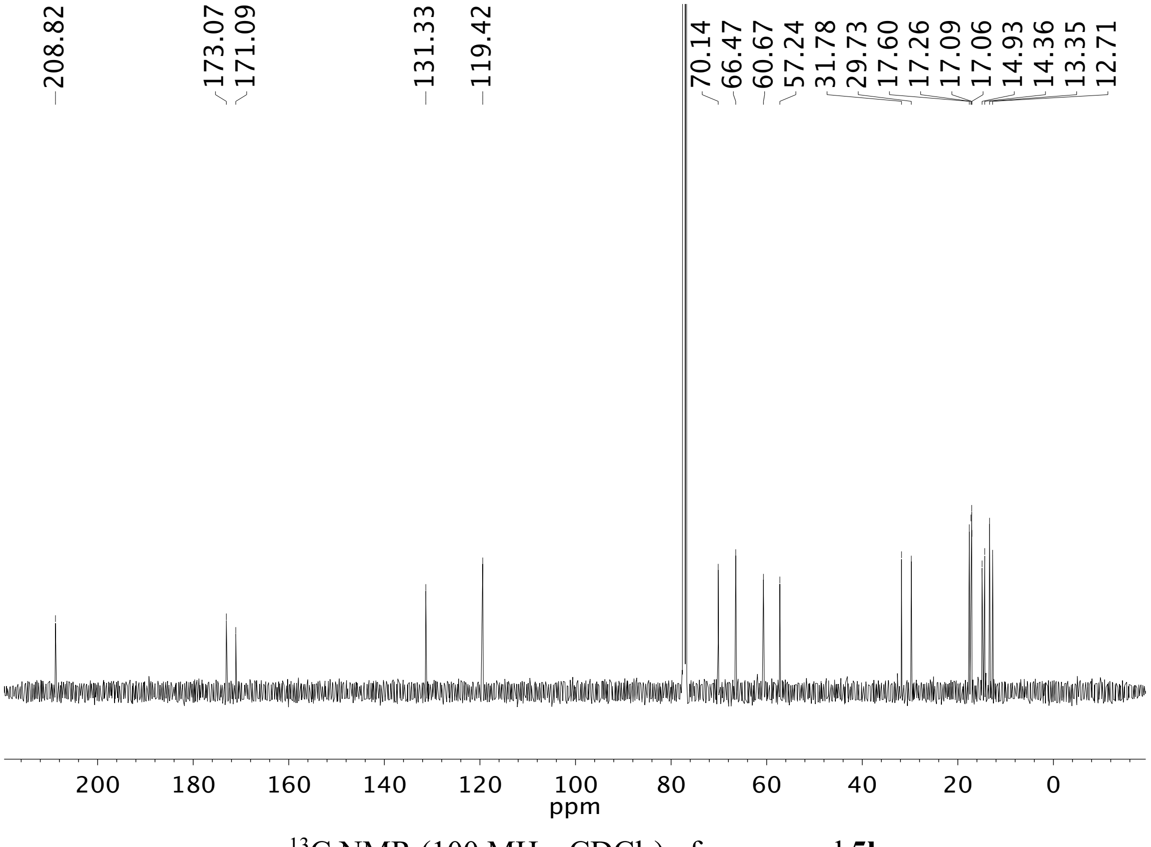

^{13}C NMR (100 MHz, $CDCl_3$) of compound **5h**.

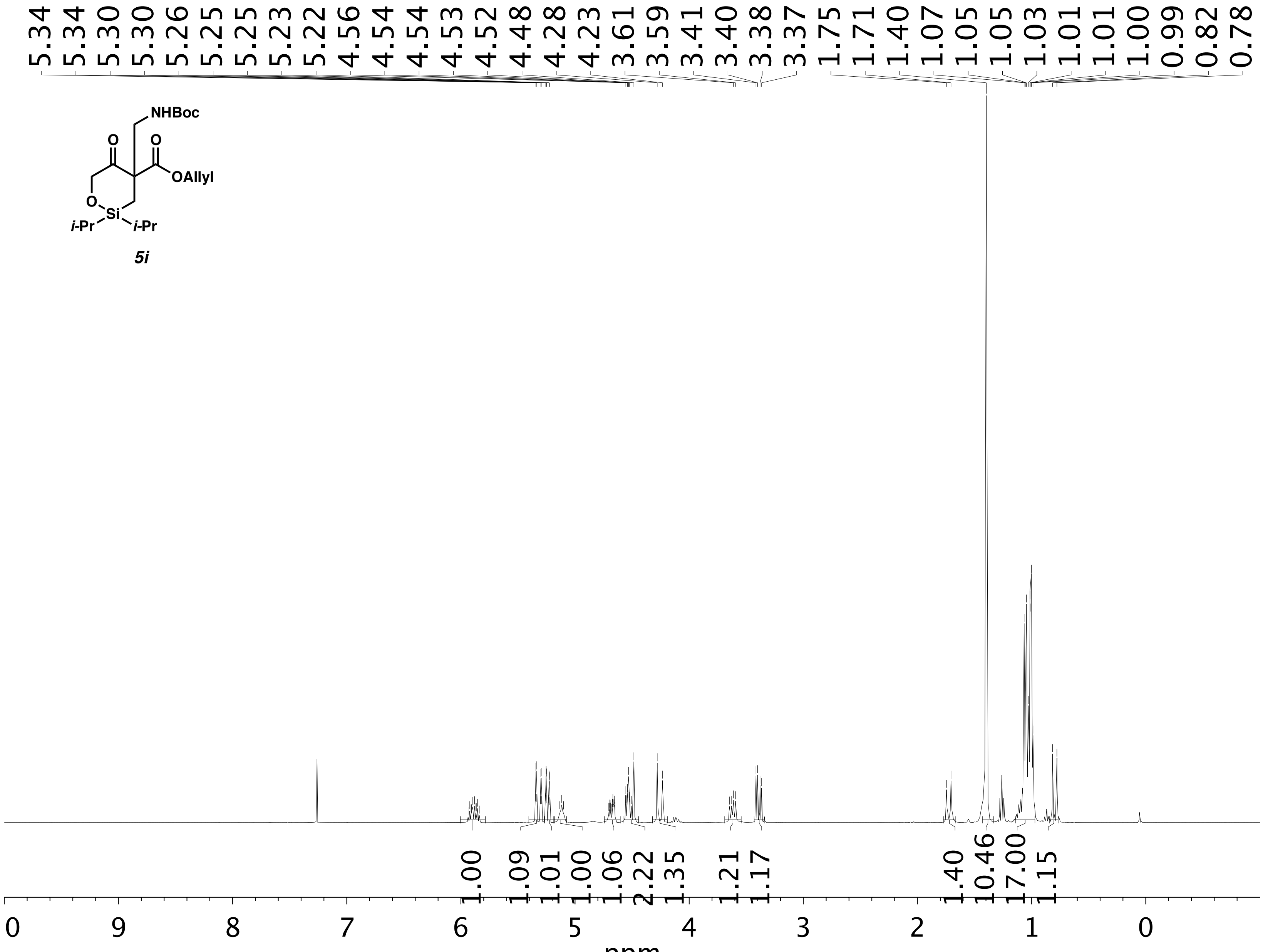

^{1}H NMR (400 MHz, $CDCl_3$) of compound **5i**

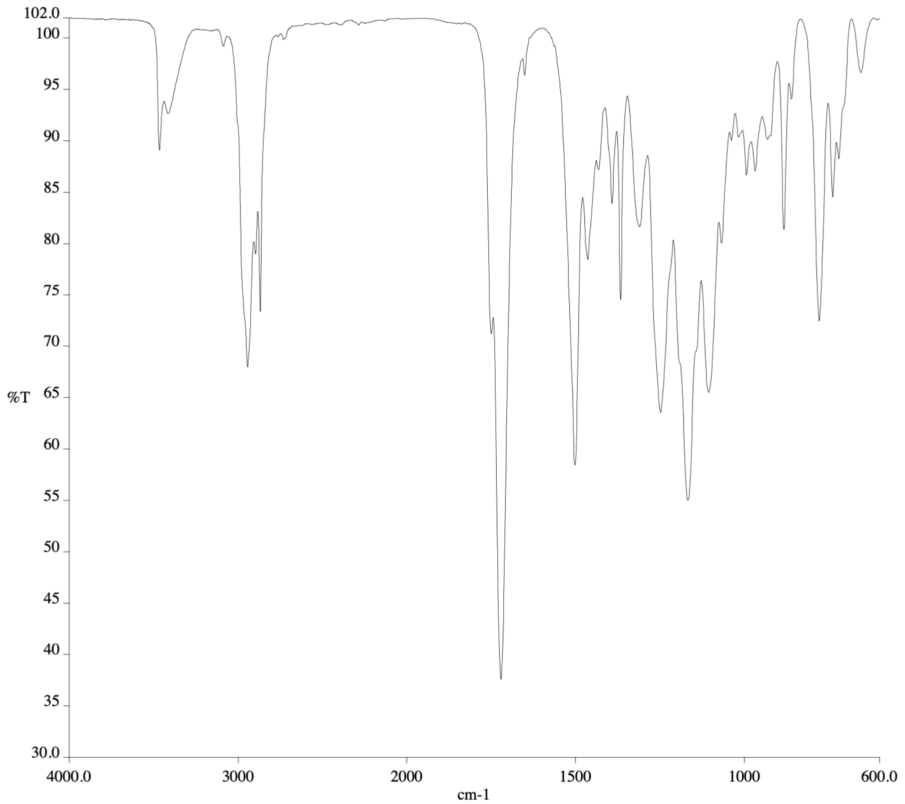

Infrared spectrum (Thin Film, NaCl) of compound **5i**.

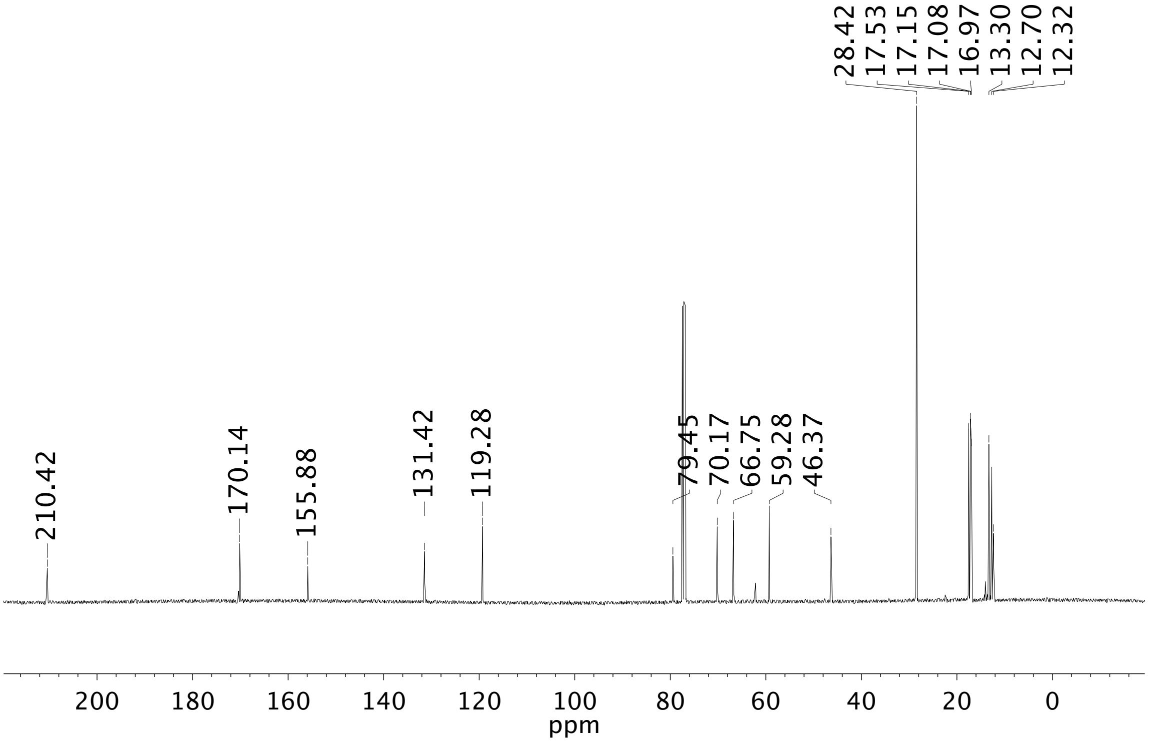

^{13}C NMR (100 MHz, $CDCl_3$) of compound **5i**.

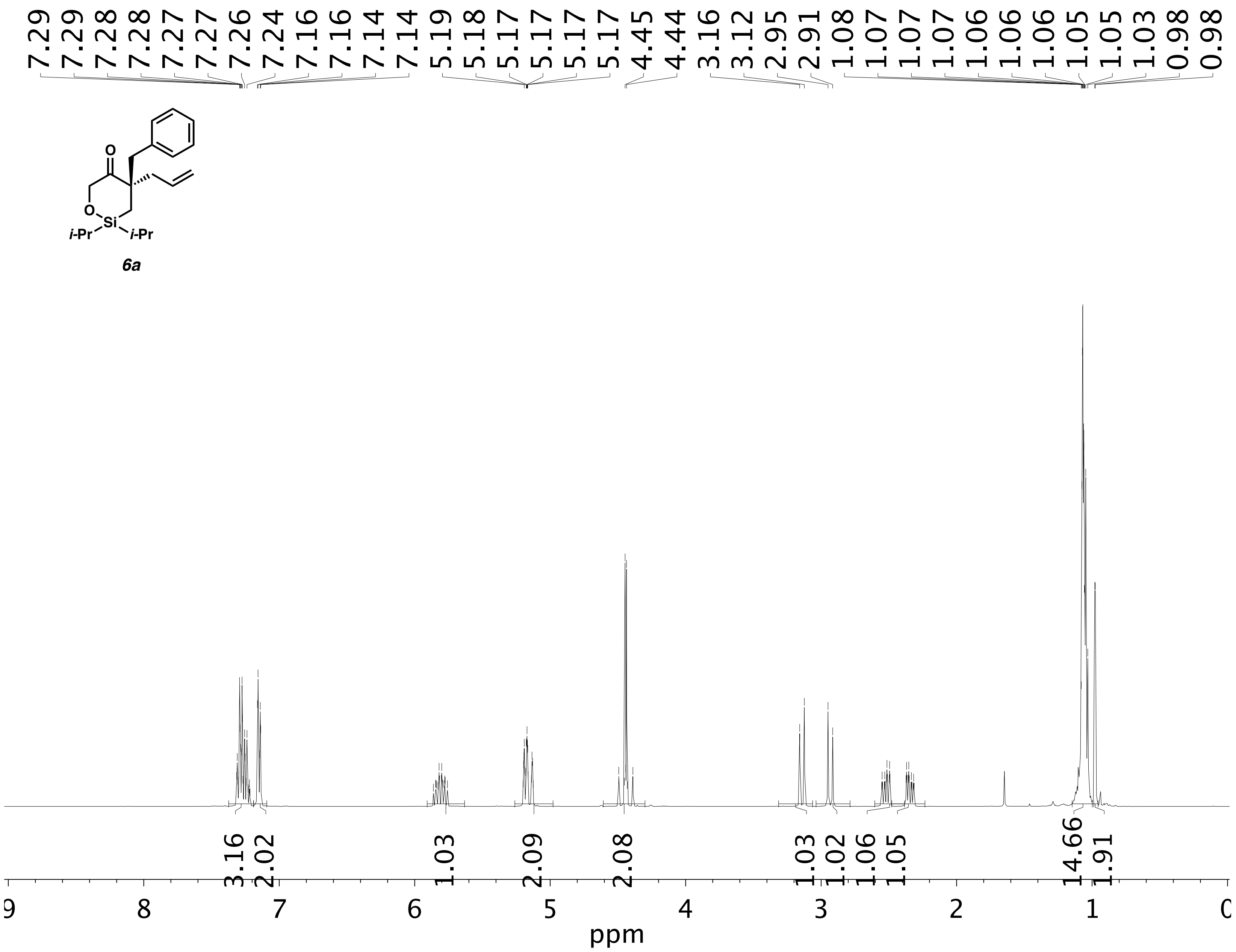

^{1}H NMR (400 MHz, $CDCl_3$) of compound **6a**.

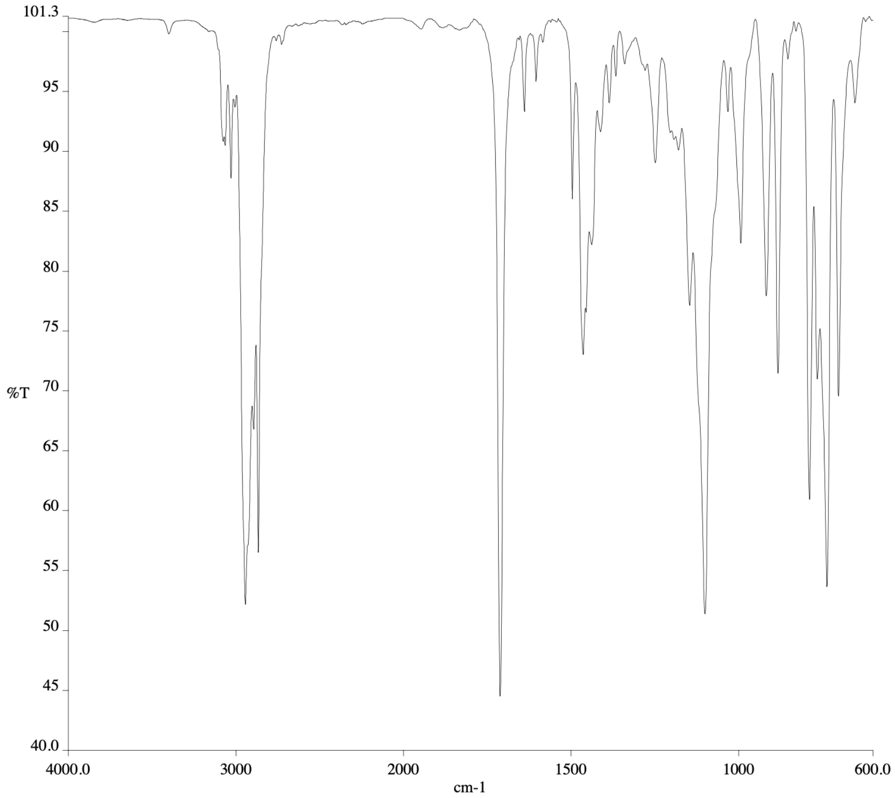

Infrared spectrum (Thin Film, NaCl) of compound **6a**.

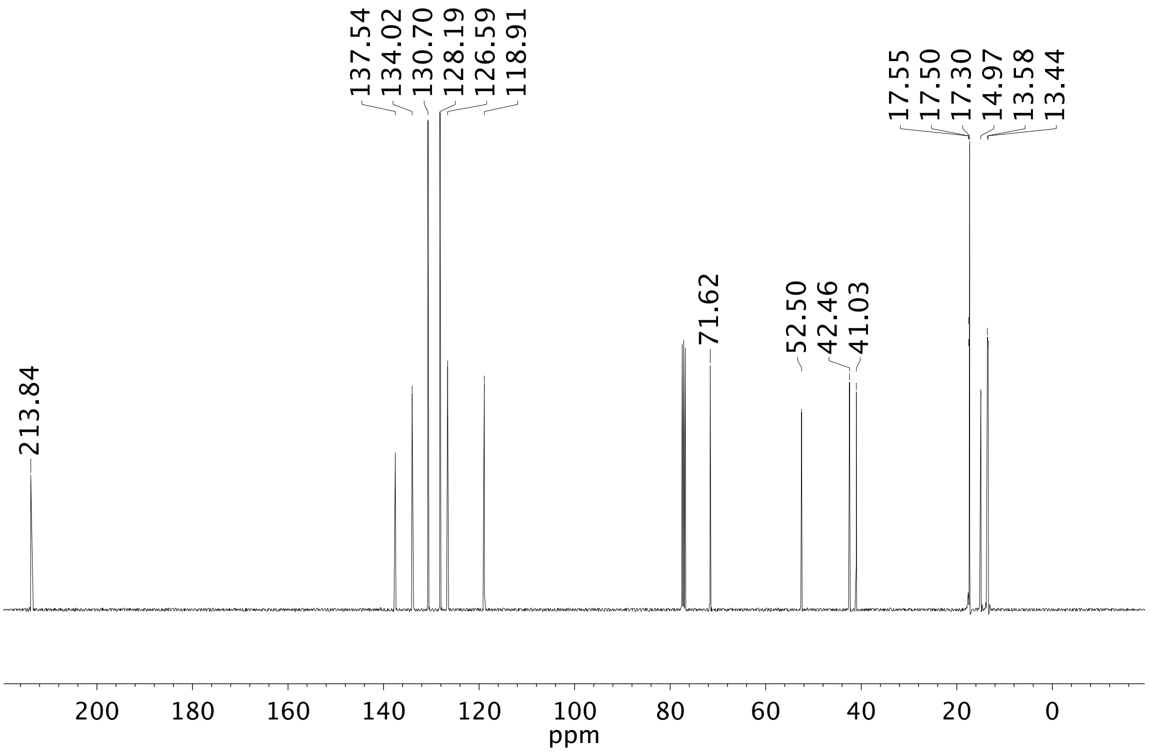

^{13}C NMR (100 MHz, $CDCl_3$) of compound **6a**.

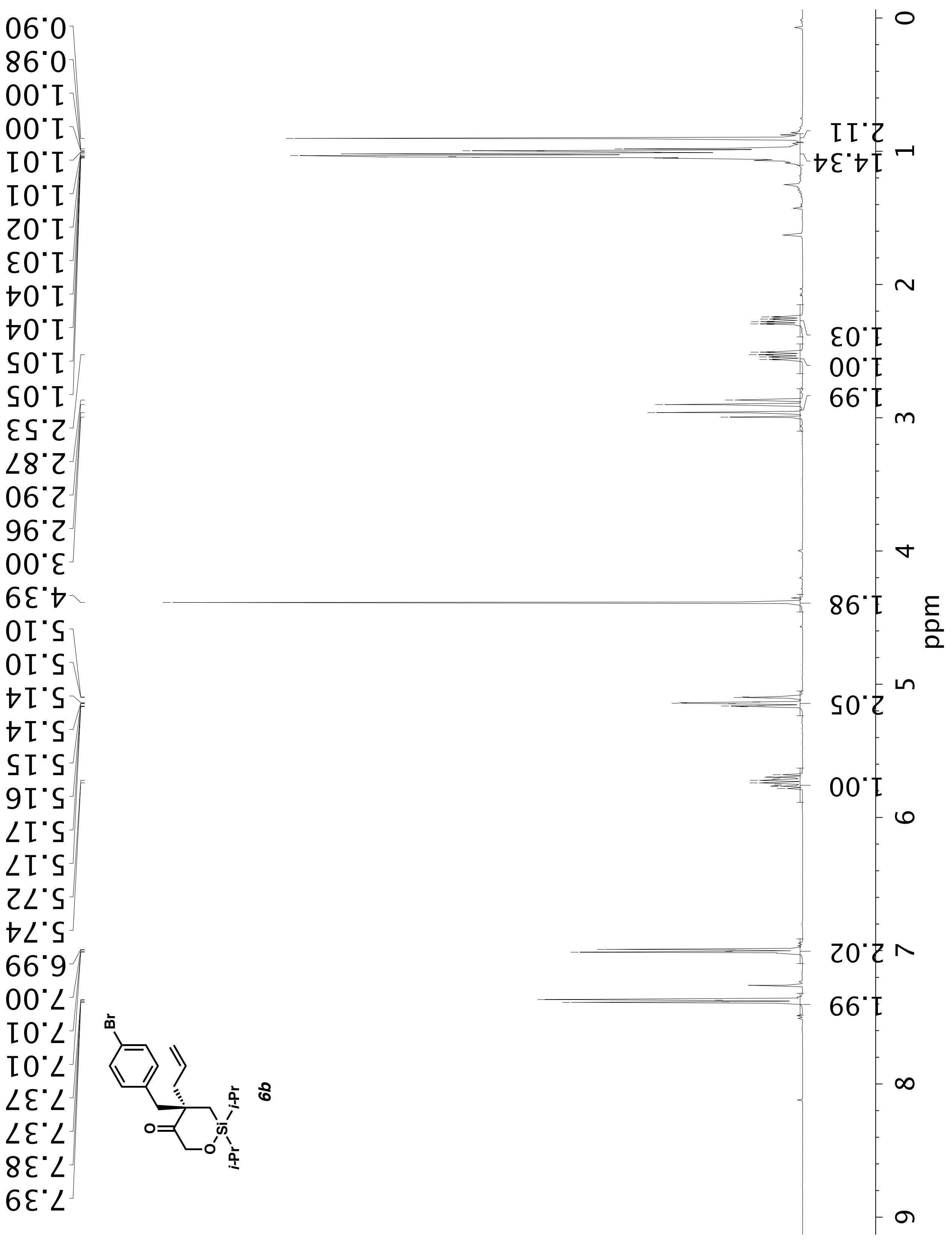

^1H NMR (400 MHz, $CDCl_3$) of compound **6b**.

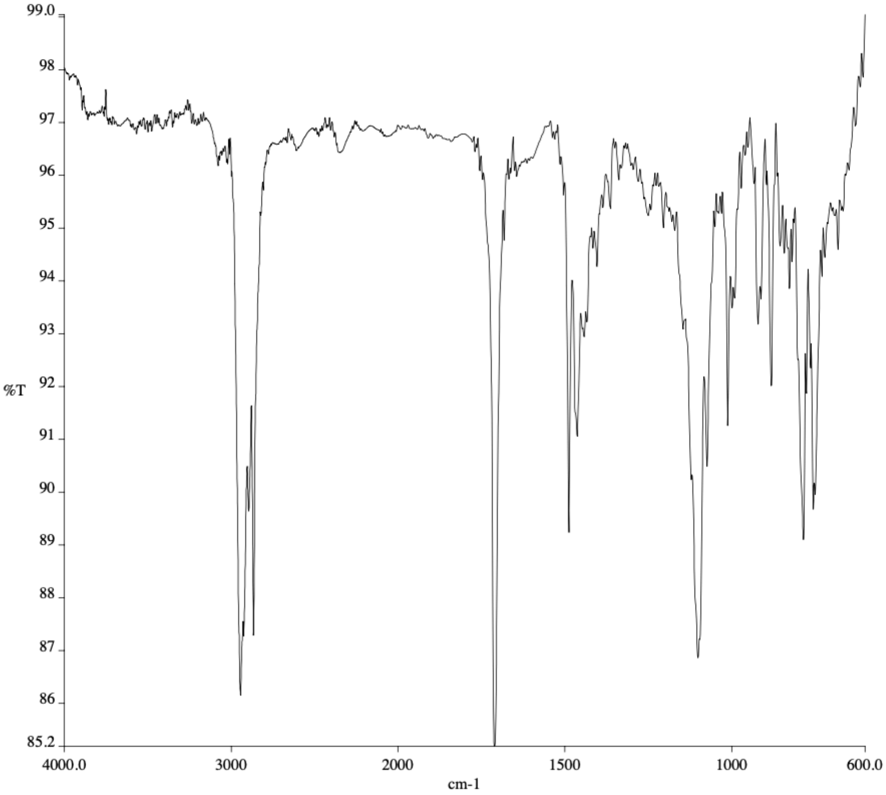

Infrared spectrum (Thin Film, NaCl) of compound **6b**.

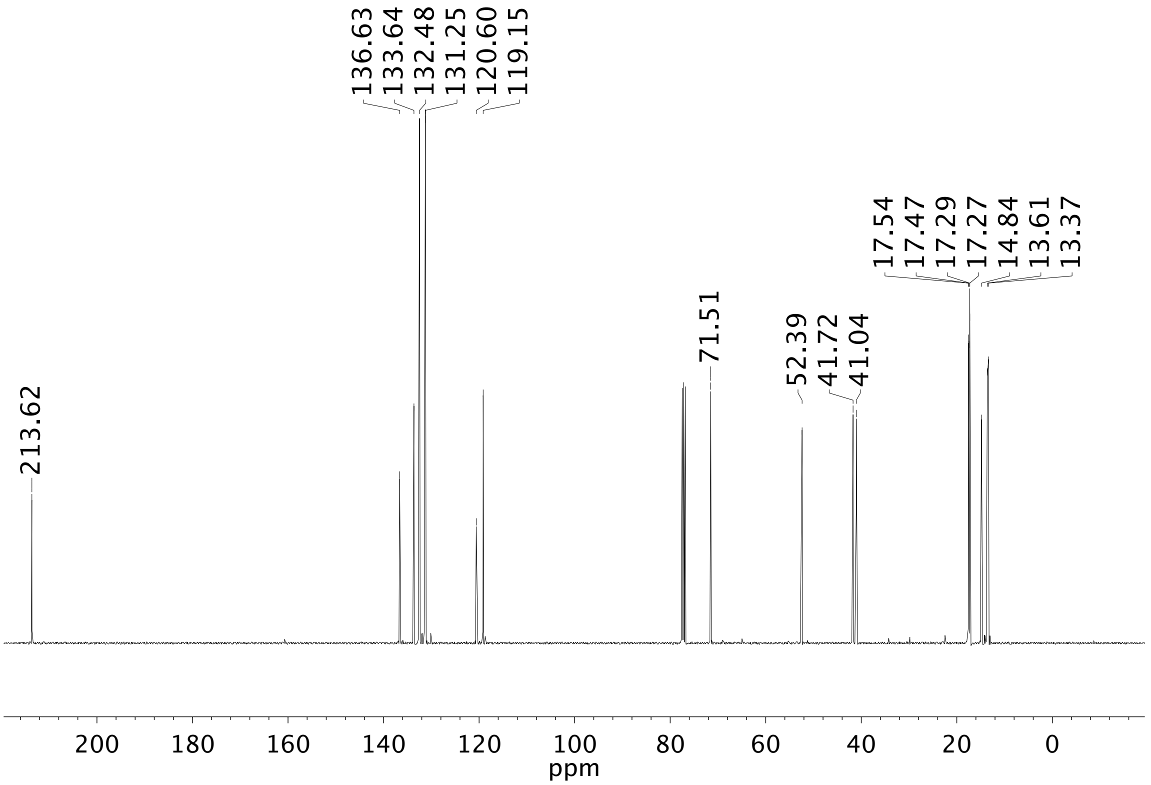

^{13}C NMR (100 MHz, $CDCl_3$) of compound **6b**.

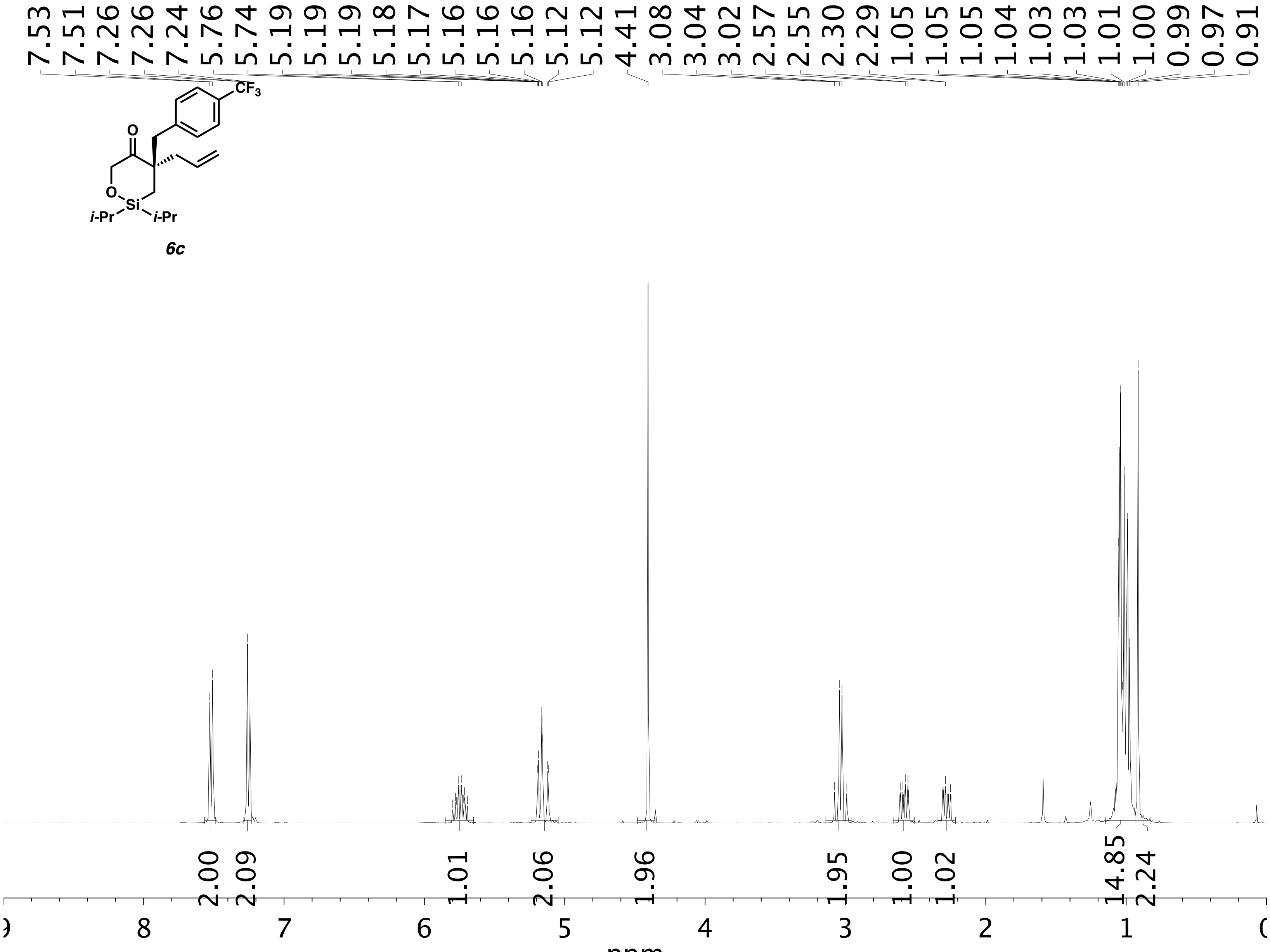

^{1}H NMR (400 MHz, $CDCl_3$) of compound **6c**.

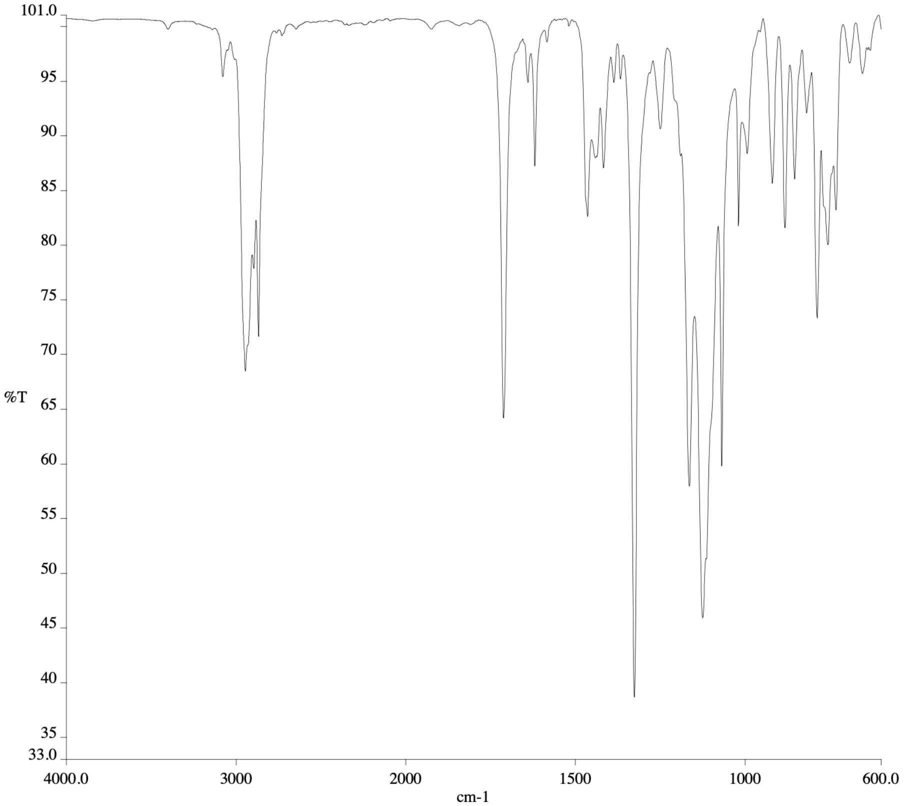

Infrared spectrum (Thin Film, NaCl) of compound **6c**.

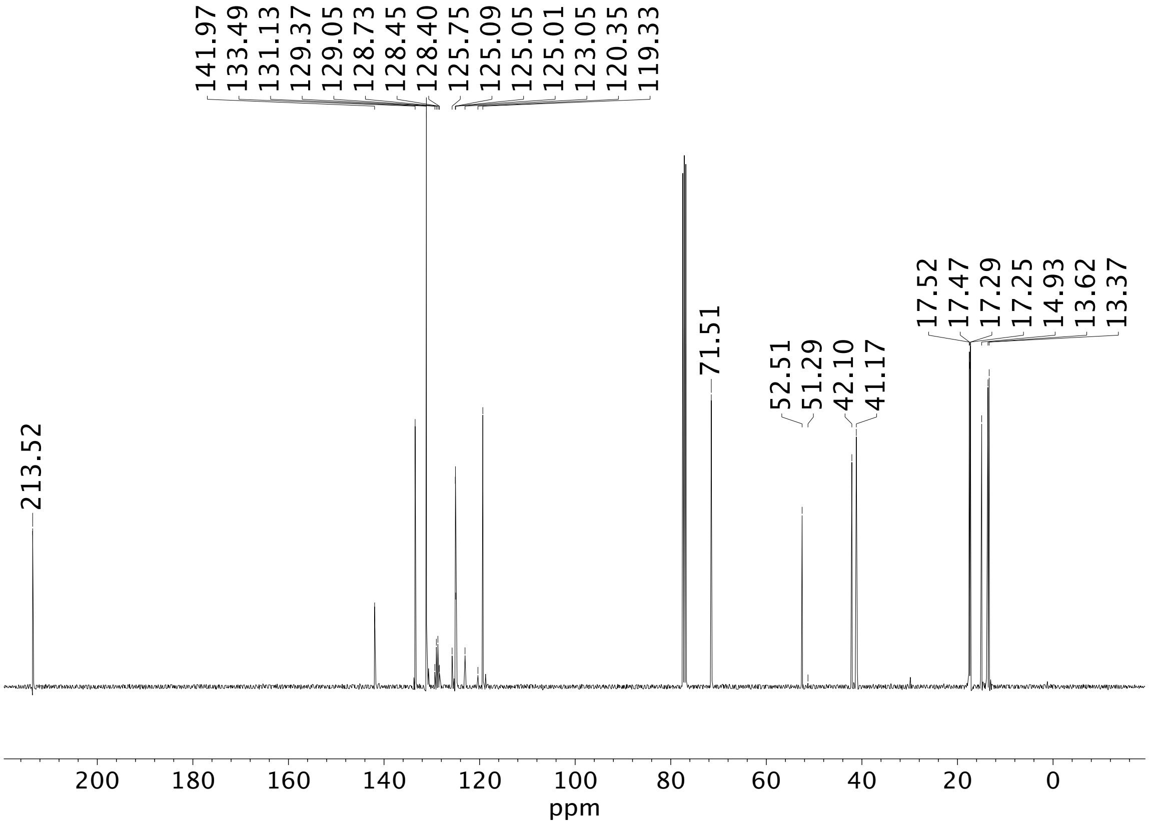

^{13}C NMR (100 MHz, $CDCl_3$) of compound **6c**.

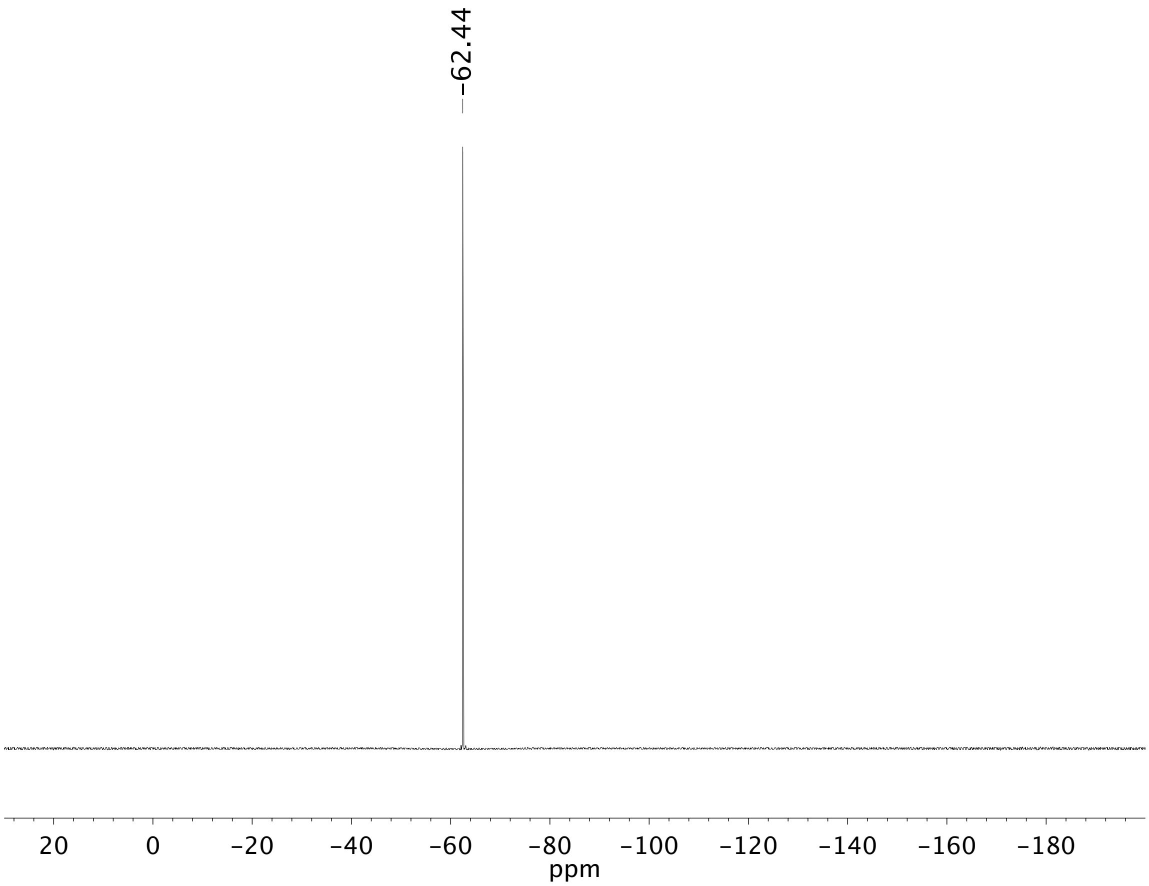

^{19}F NMR (282 MHz, $CDCl_3$) of compound **6c**.

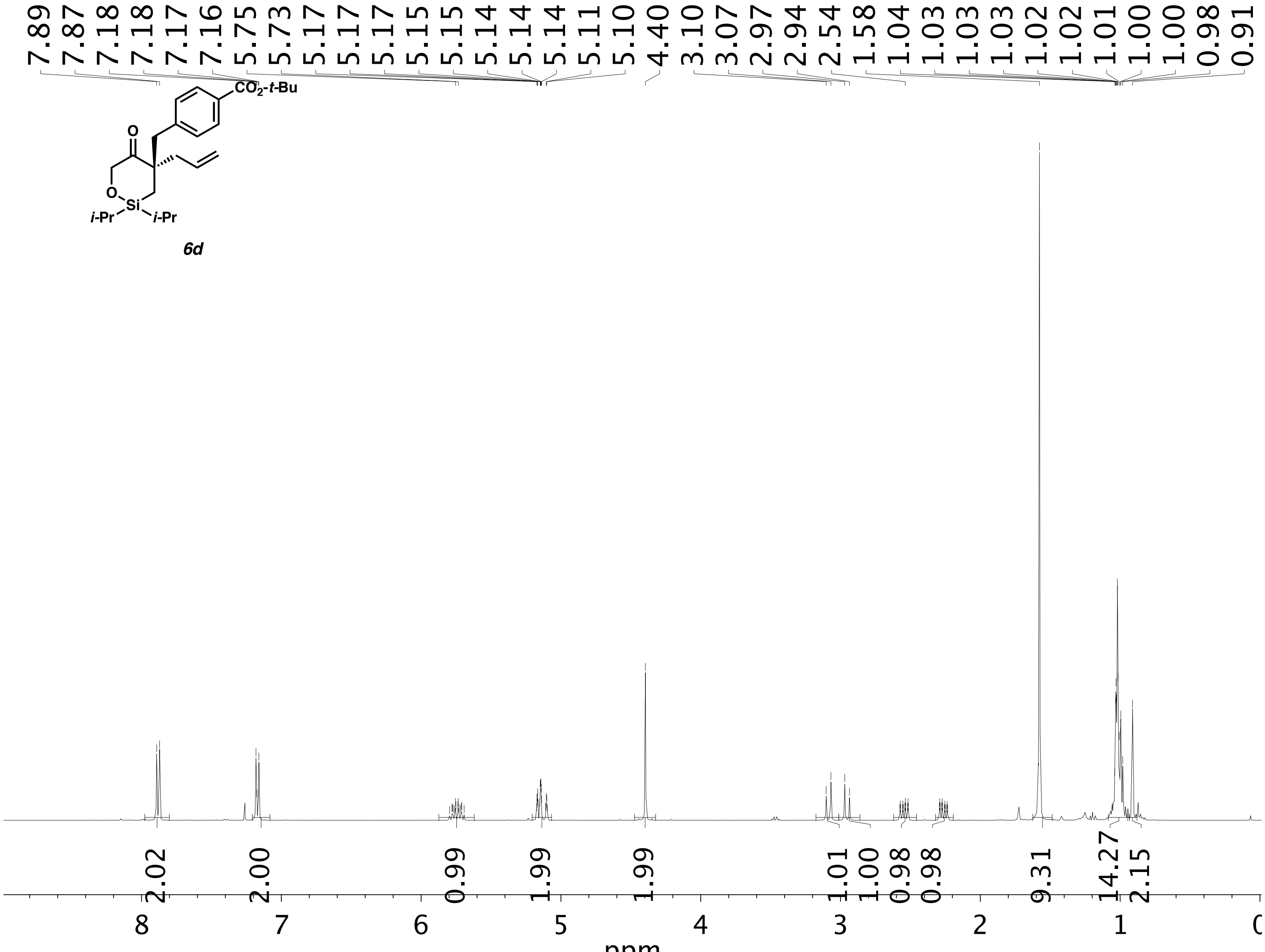

^{1}H NMR (400 MHz, $CDCl_3$) of compound **6d**

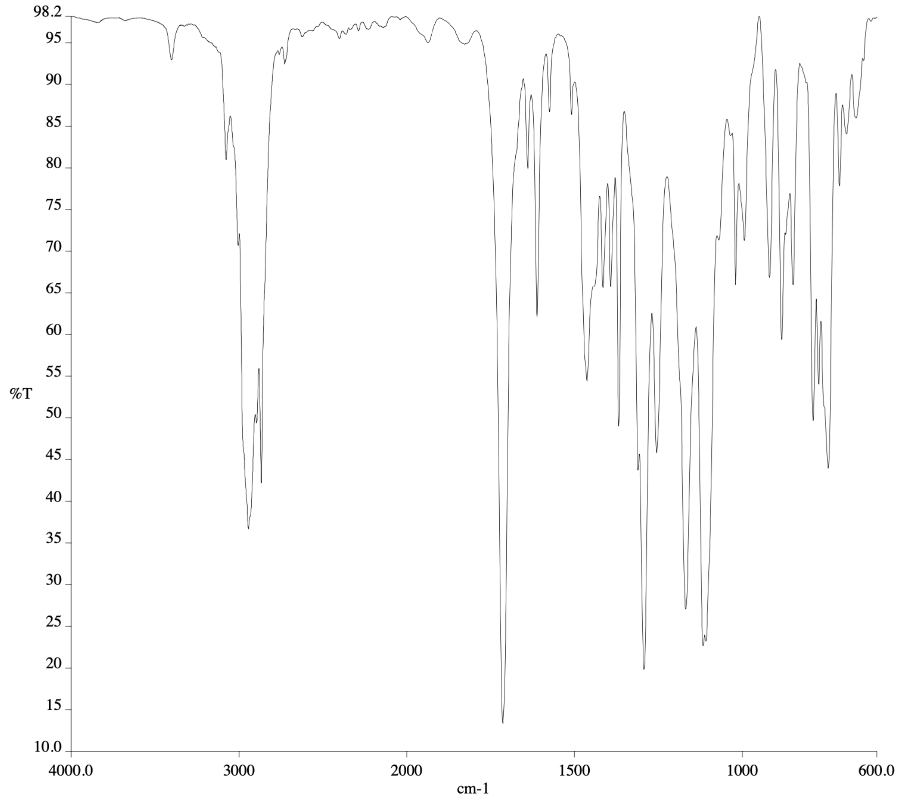

Infrared spectrum (Thin Film, NaCl) of compound **6d**.

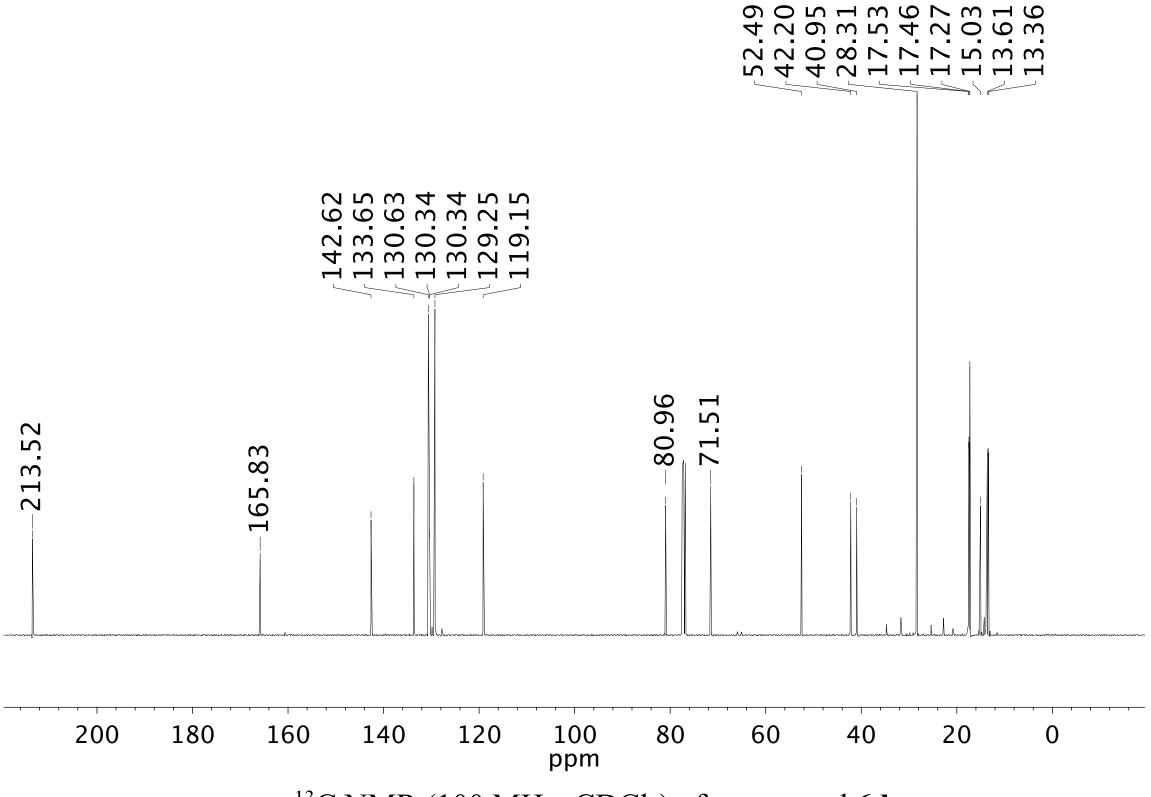

^{13}C NMR (100 MHz, $CDCl_3$) of compound **6d**.

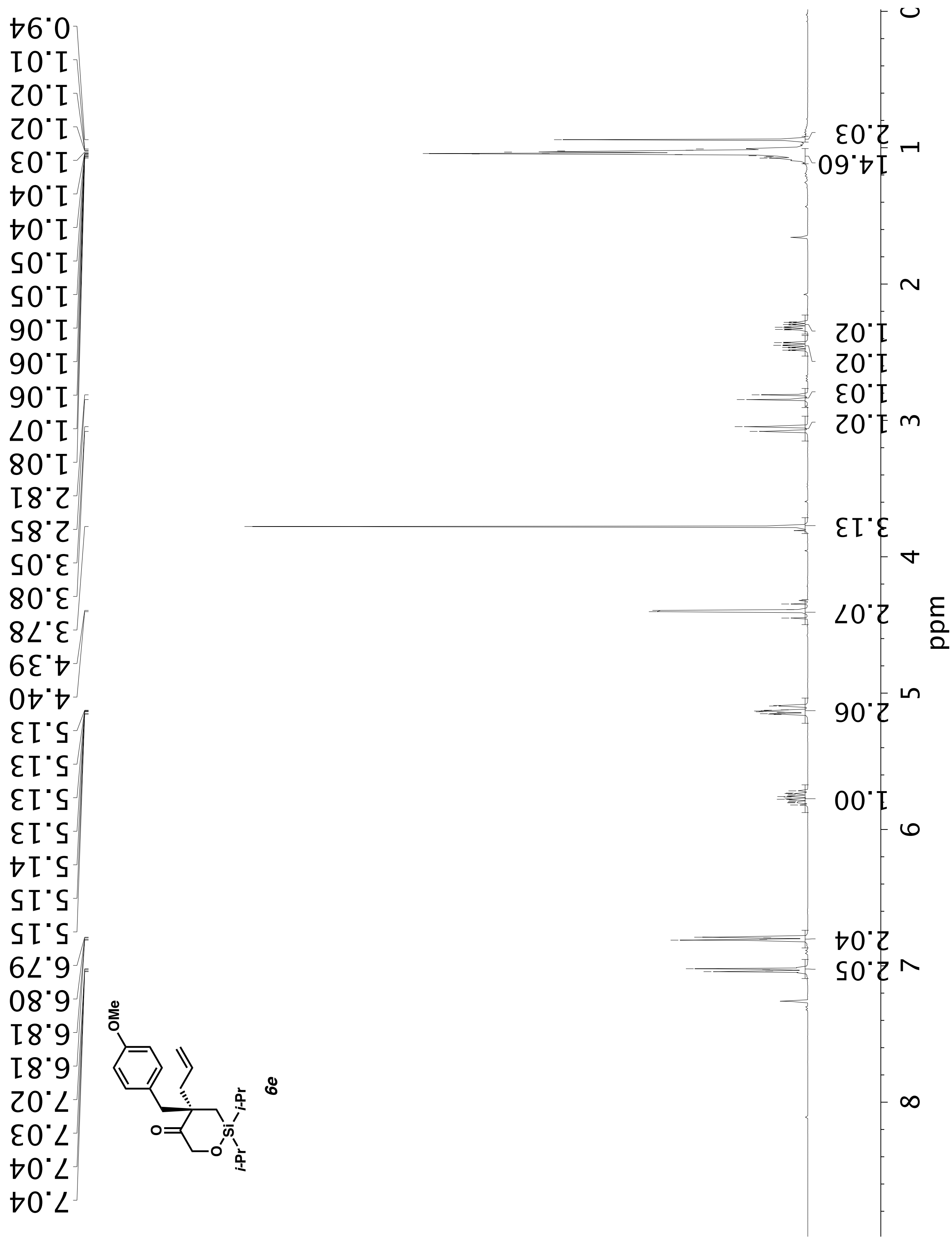

^1H NMR (400 MHz, $CDCl_3$) of compound **6e**.

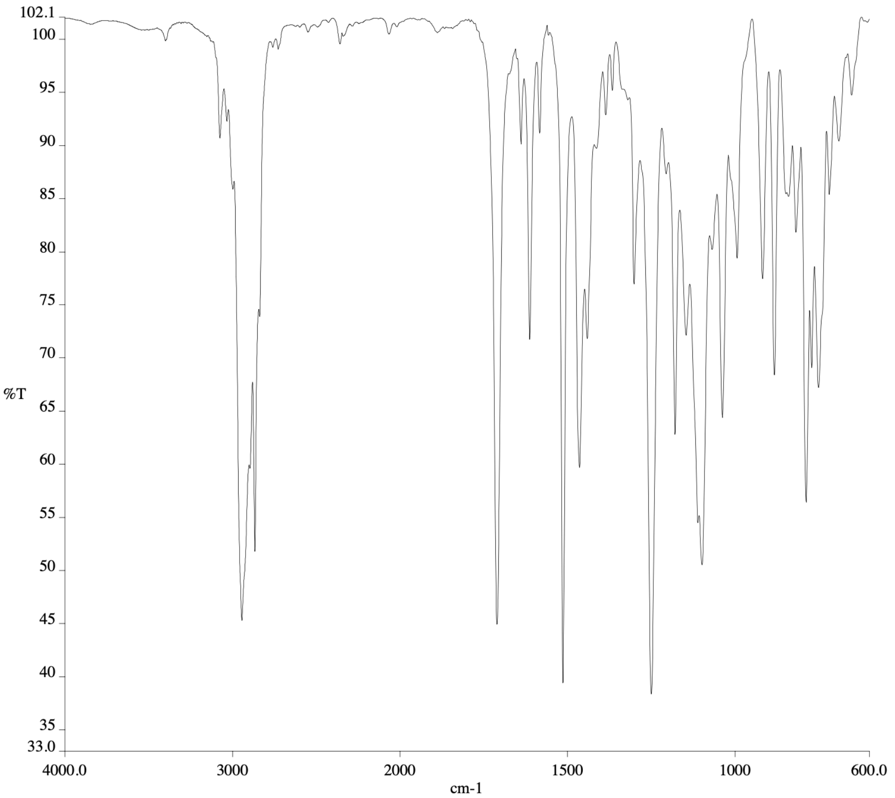

Infrared spectrum (Thin Film, NaCl) of compound **6e**.

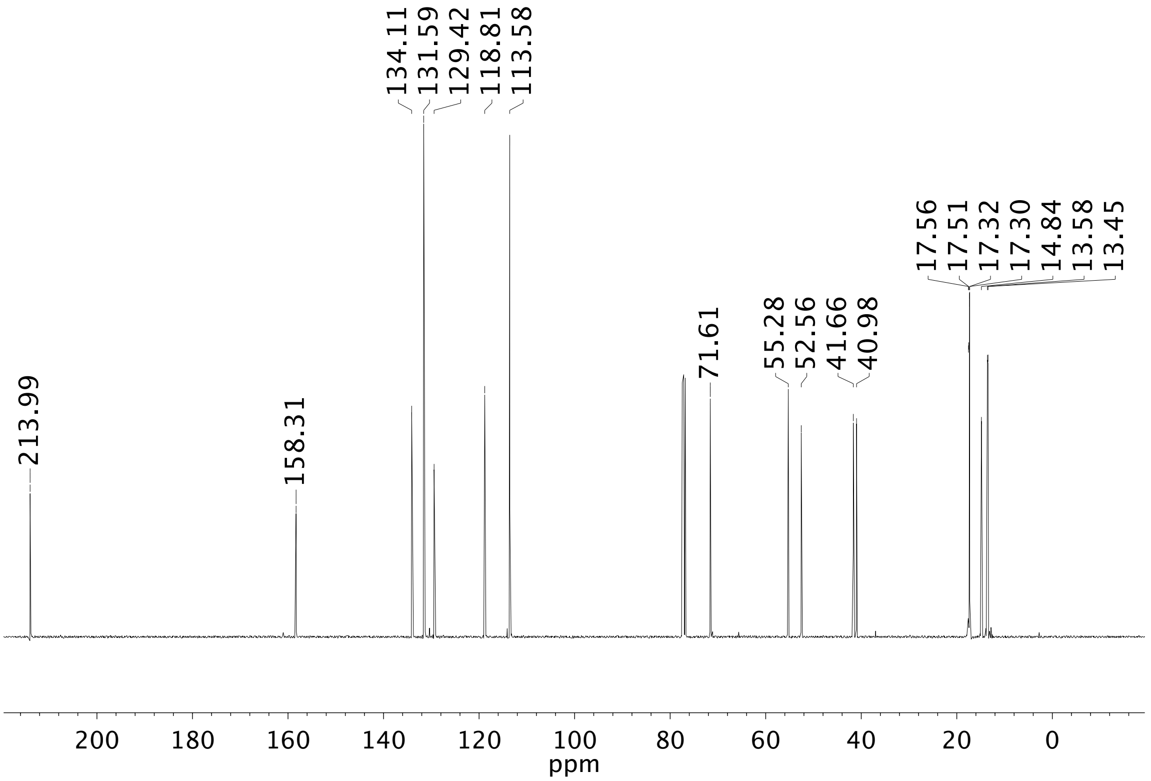

^{13}C NMR (100 MHz, $CDCl_3$) of compound **6e**.

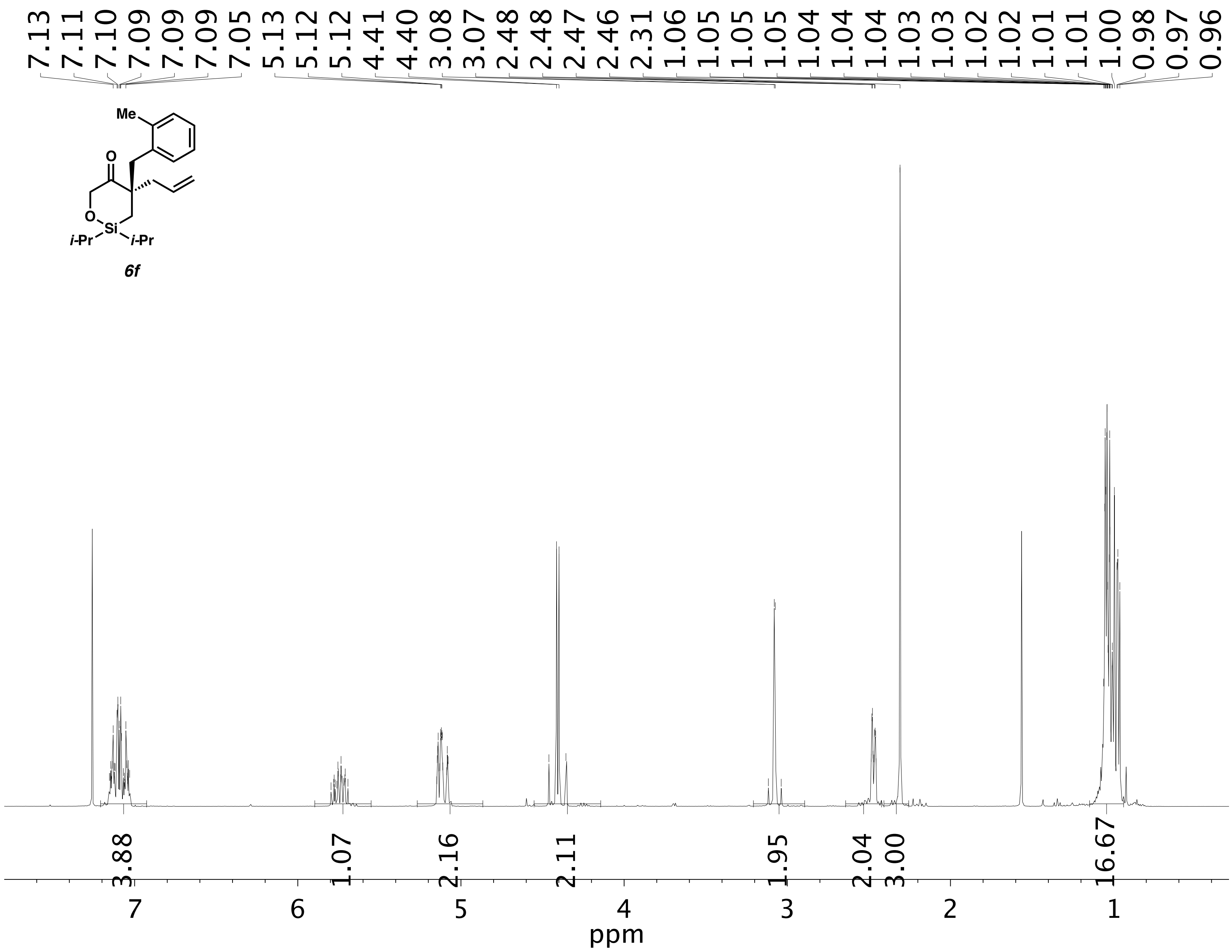

^{1}H NMR (400 MHz, $CDCl_3$) of compound **6f**.

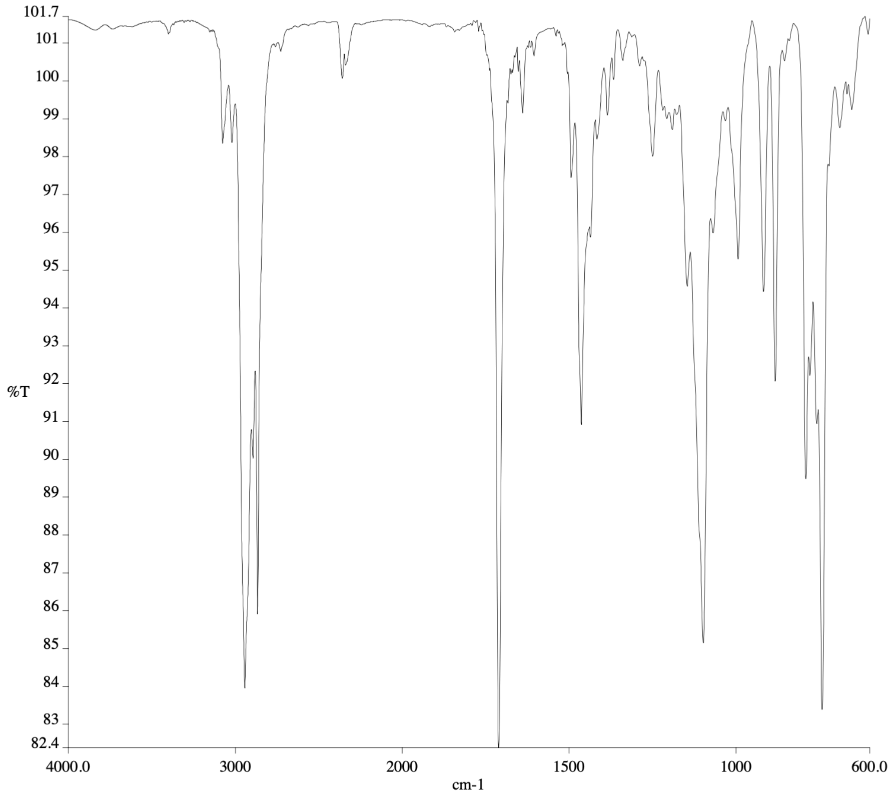

Infrared spectrum (Thin Film, NaCl) of compound **6f**.

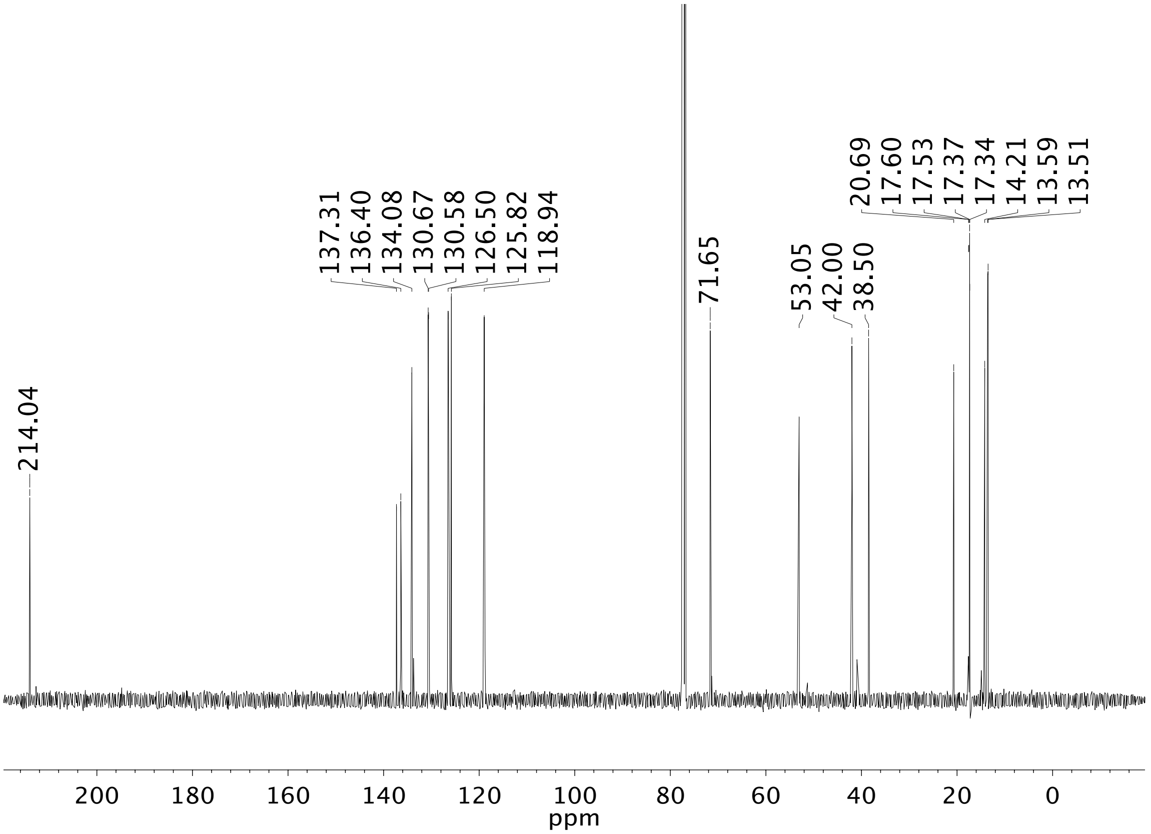

^{13}C NMR (100 MHz, $CDCl_3$) of compound **6f**.

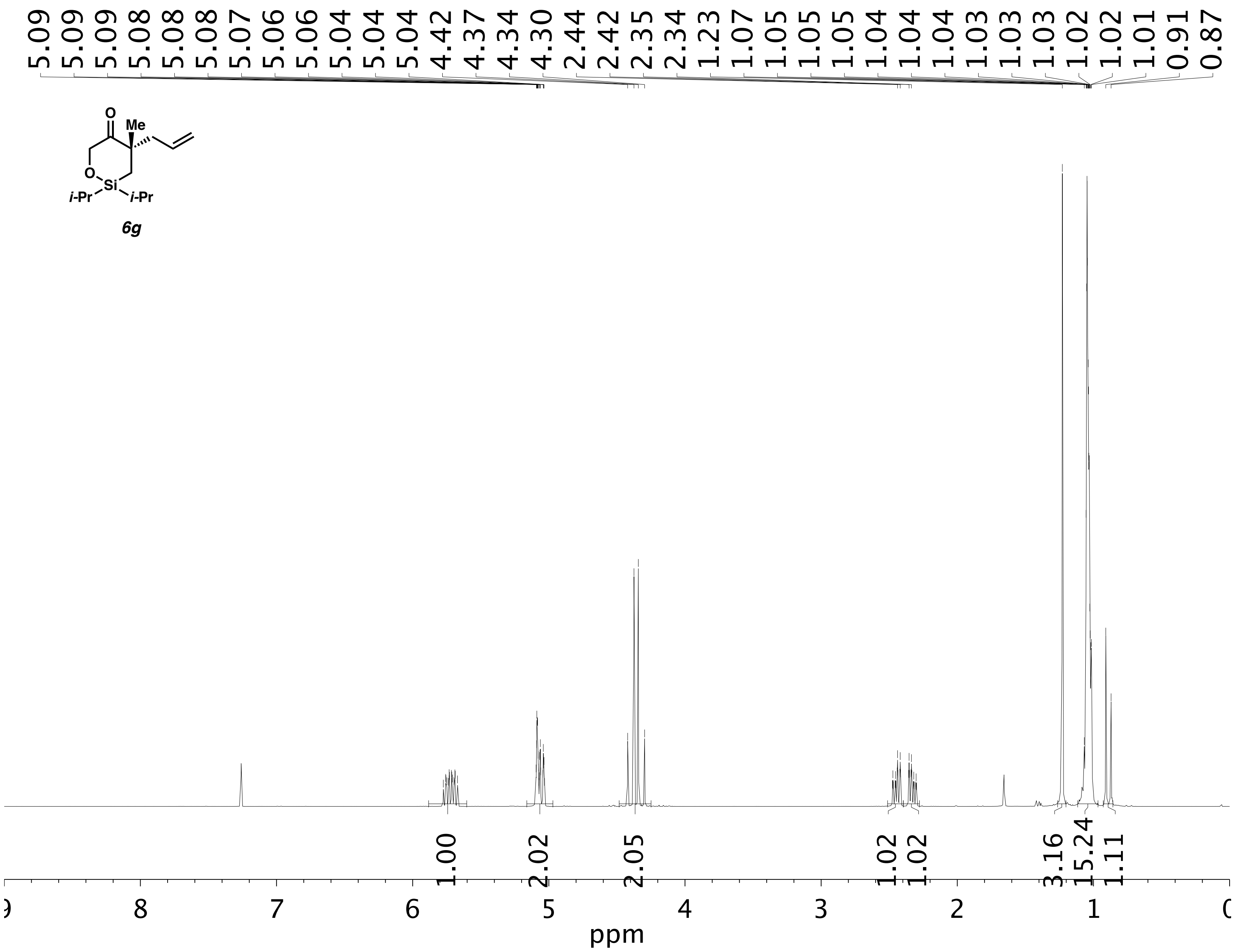

^{1}H NMR (400 MHz, $CDCl_3$) of compound **6g**.

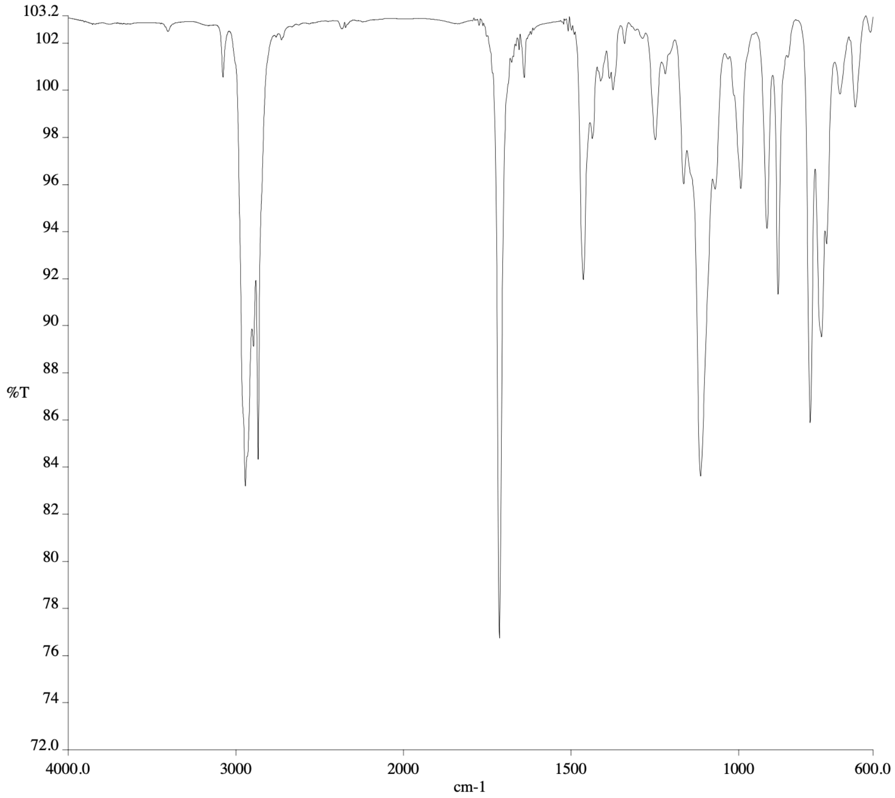

Infrared spectrum (Thin Film, NaCl) of compound **6g**.

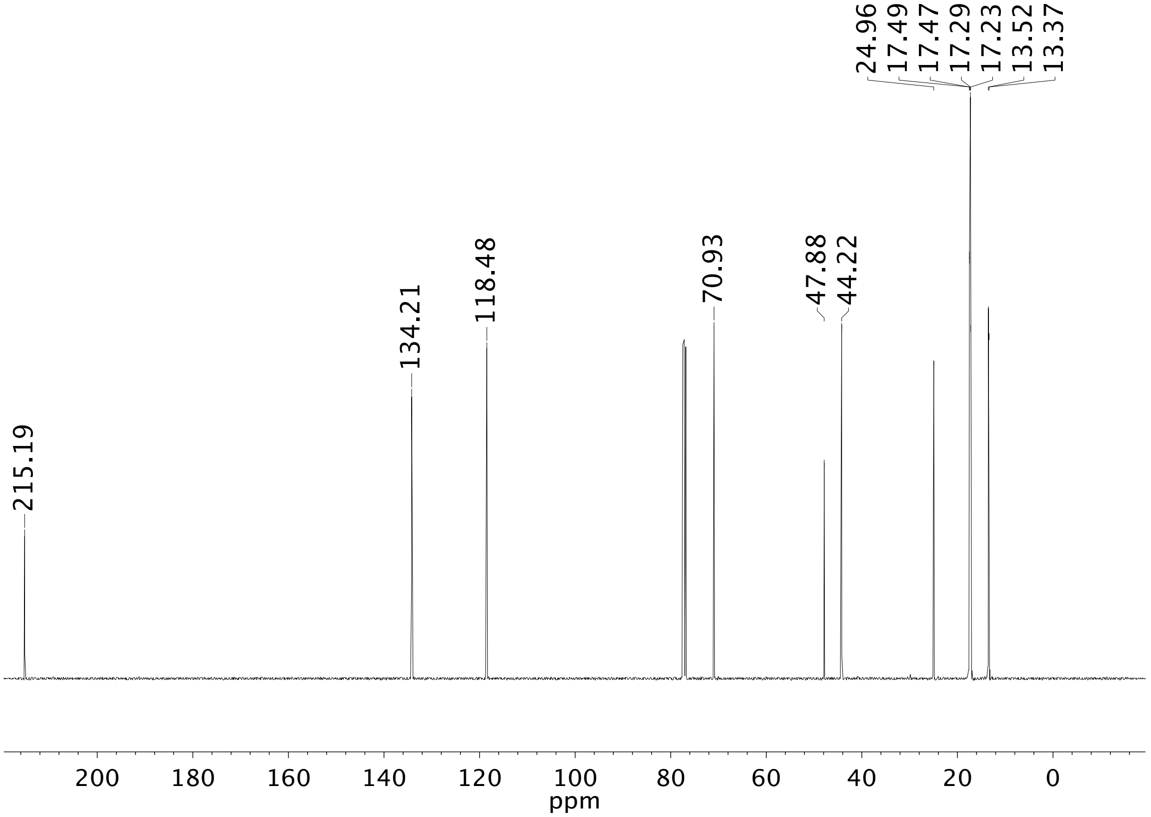

^{13}C NMR (100 MHz, $CDCl_3$) of compound **6g**.

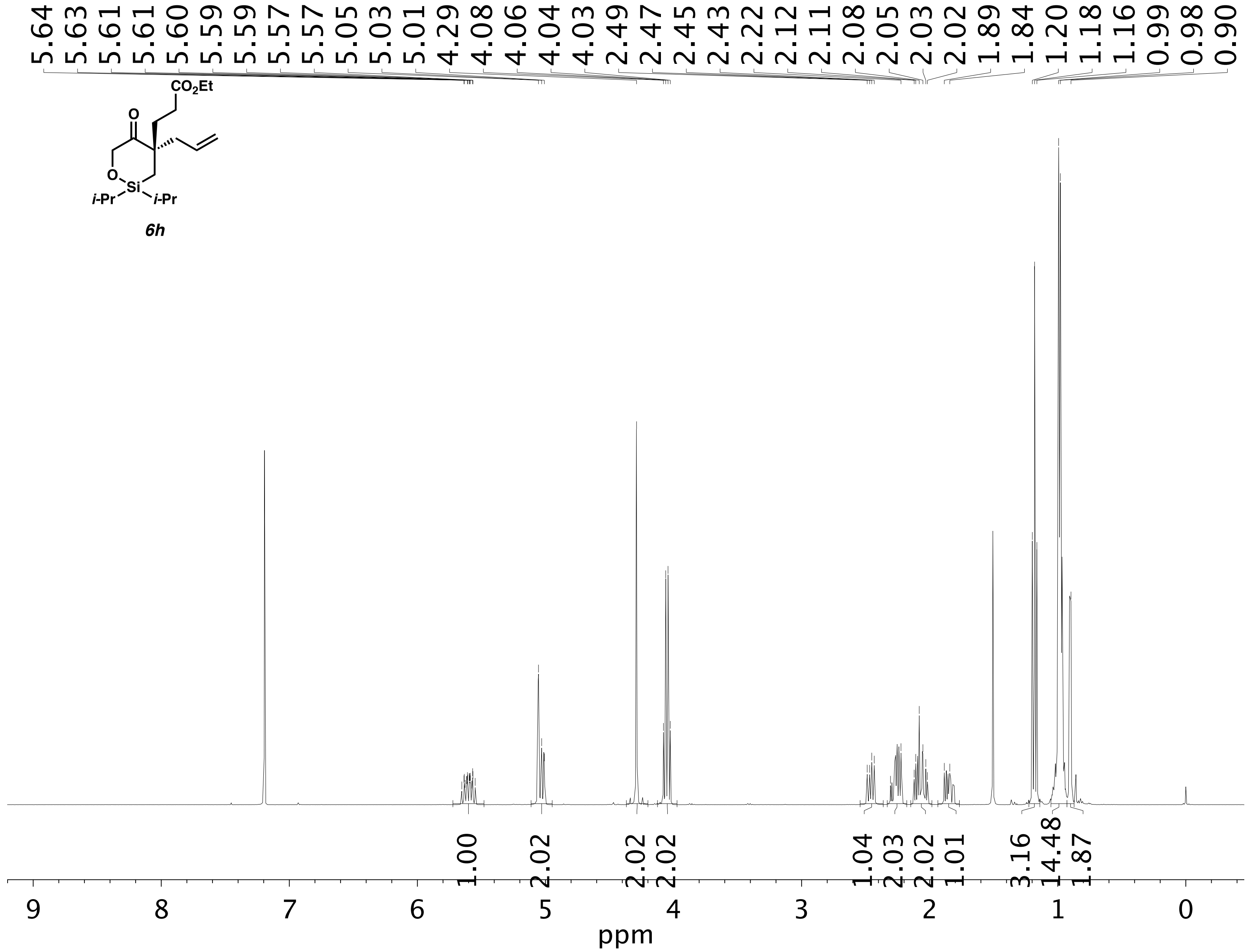

^{1}H NMR (400 MHz, $CDCl_3$) of compound **6h**.

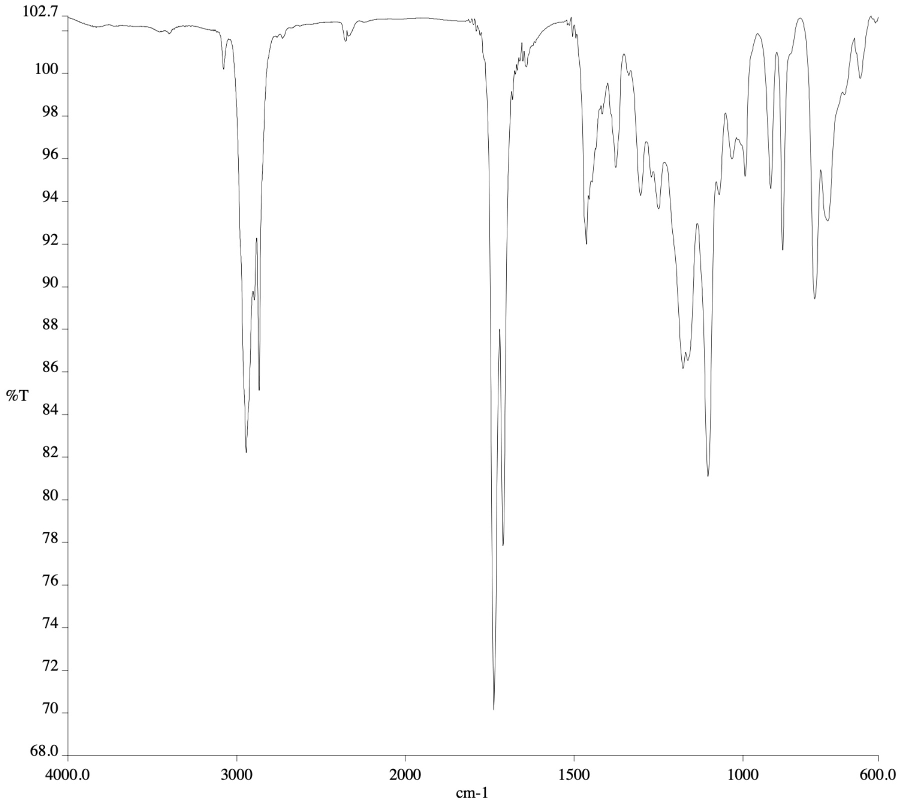

Infrared spectrum (Thin Film, NaCl) of compound **6h**.

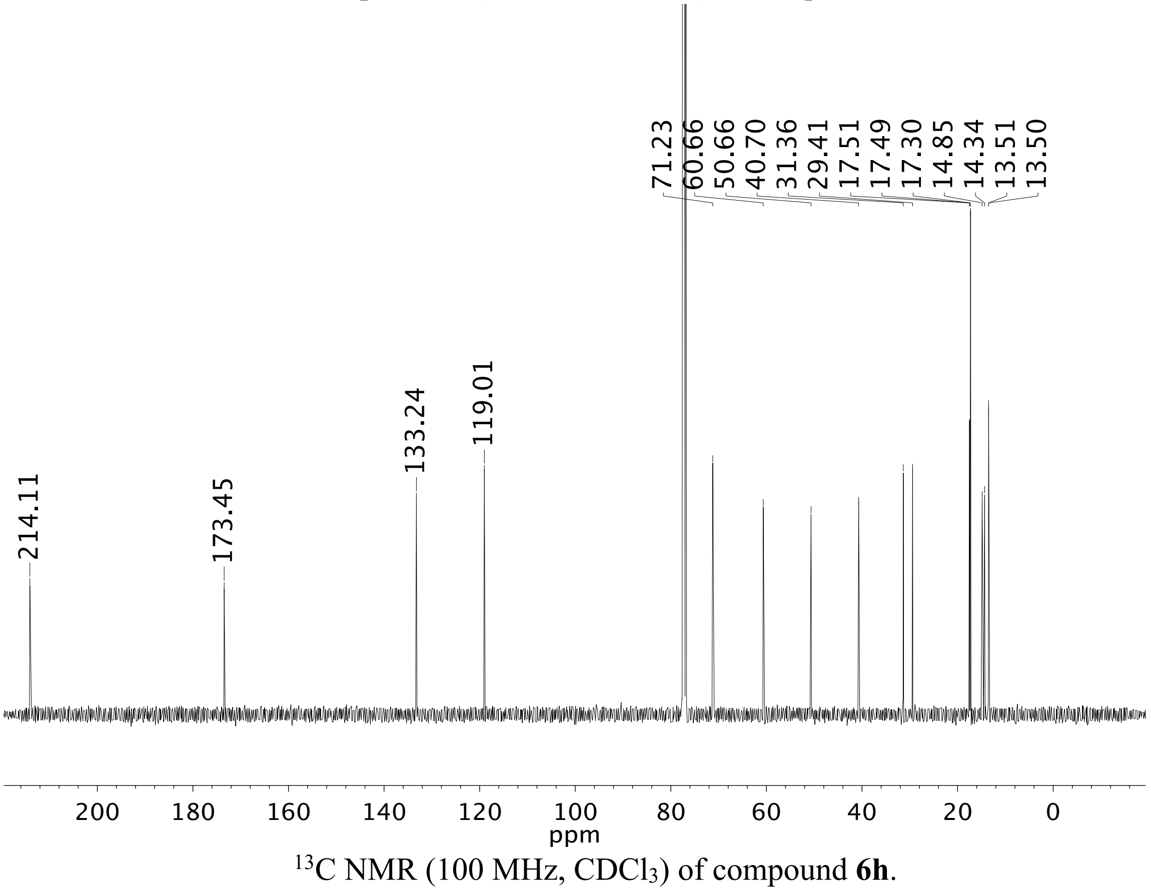

^{13}C NMR (100 MHz, $CDCl_3$) of compound **6h**.

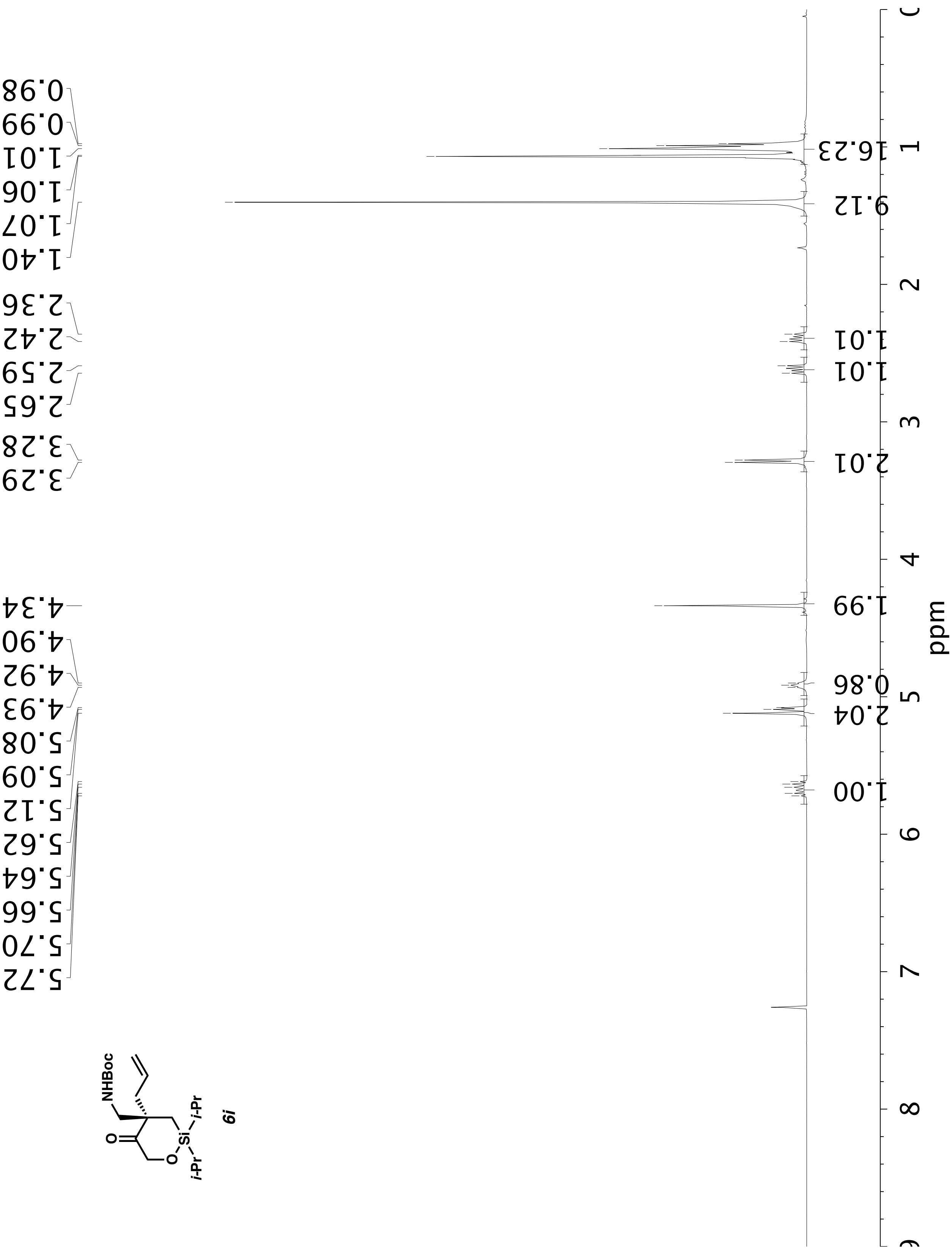

^{1}H NMR (400 MHz, $CDCl_3$) of compound **6i**.

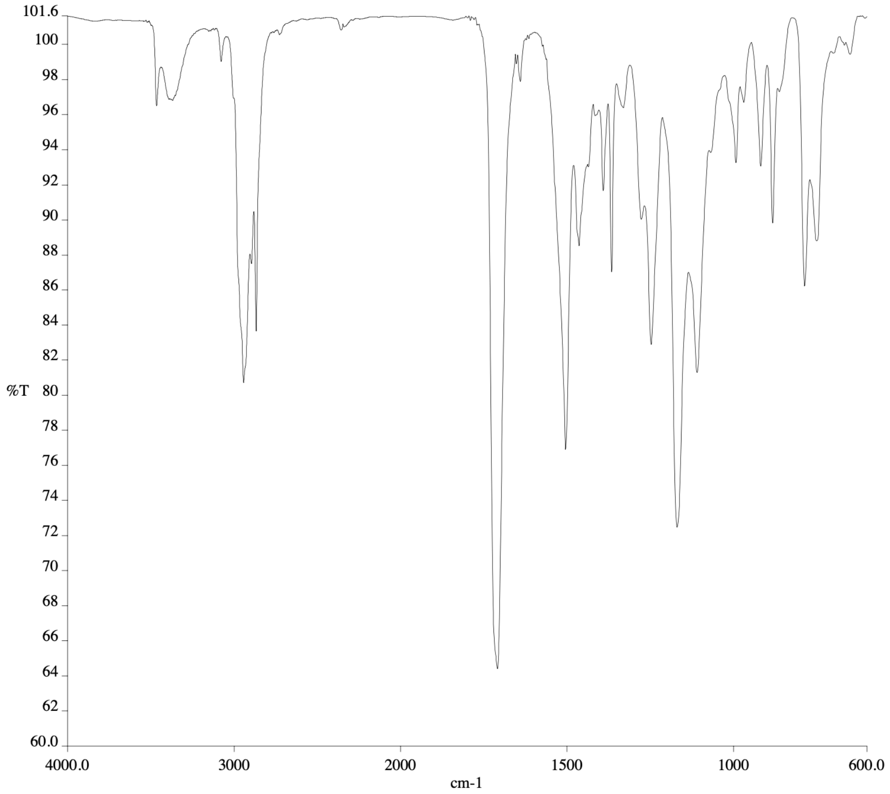

Infrared spectrum (Thin Film, NaCl) of compound **6i**.

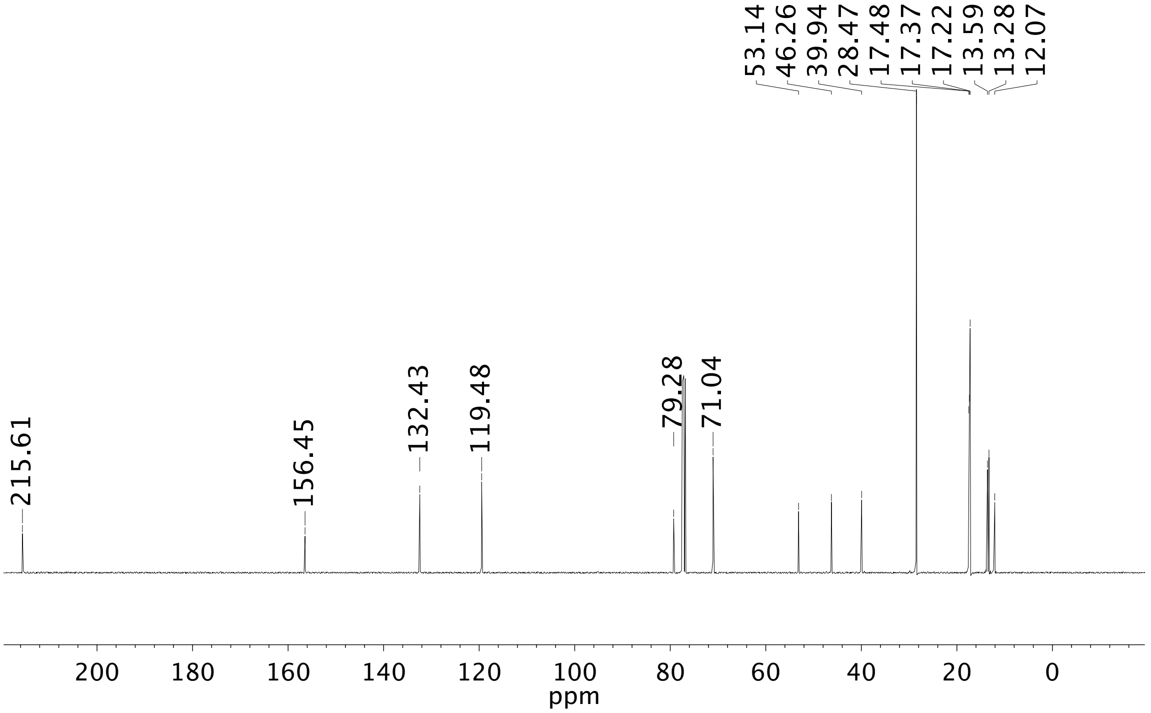

^{13}C NMR (100 MHz, $CDCl_3$) of compound **6i**.

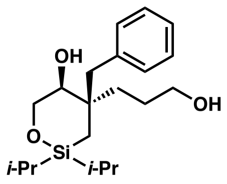

7

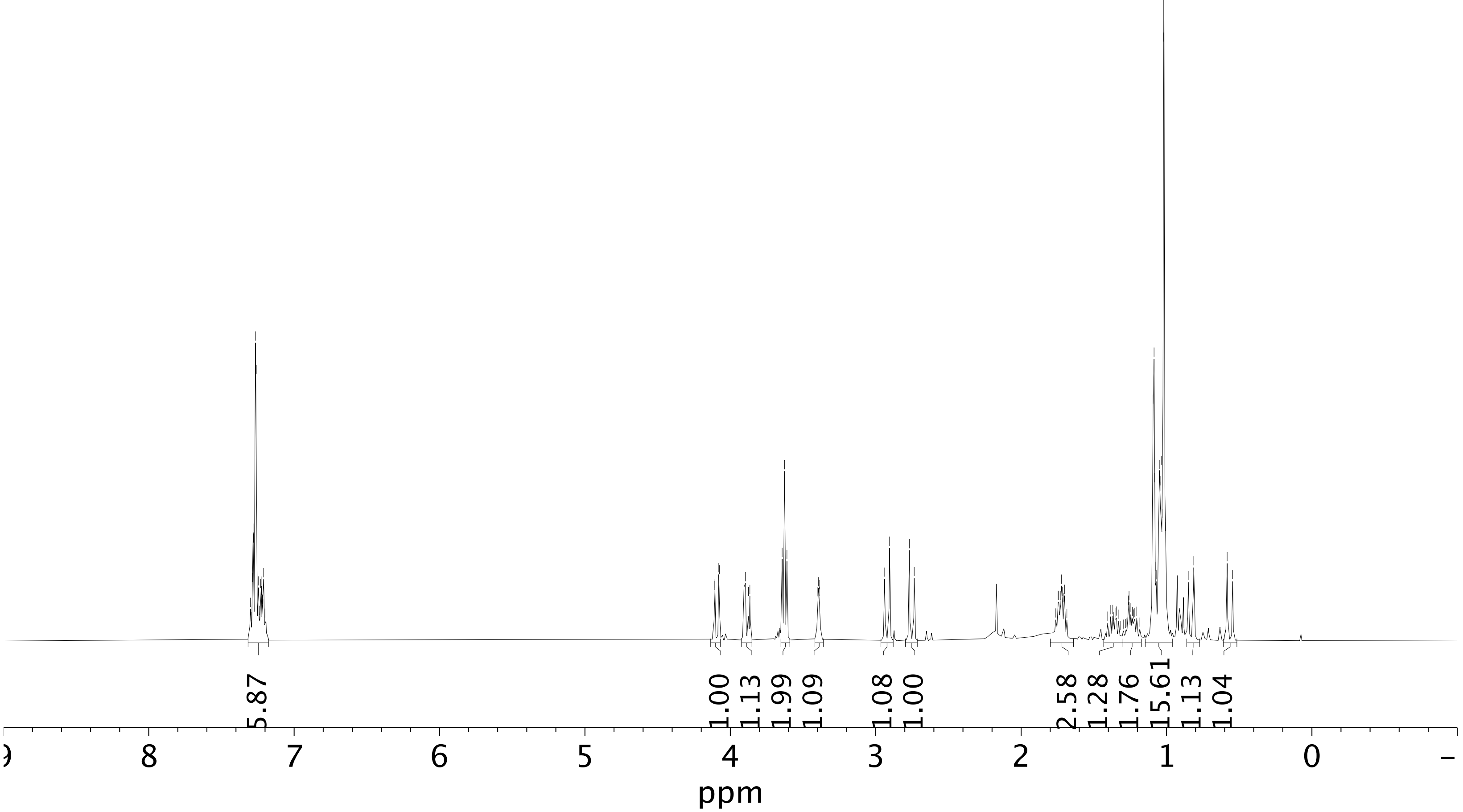

^{1}H NMR (400 MHz, $CDCl_3$) of compound **7**.

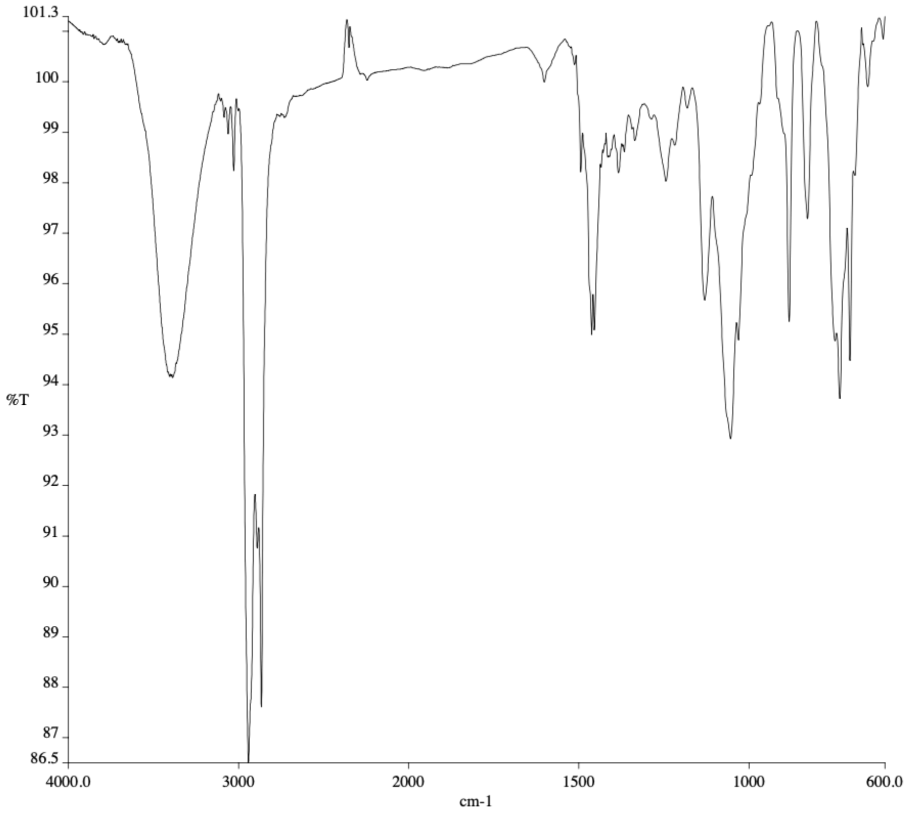

Infrared spectrum (Thin Film, NaCl) of compound **7**.

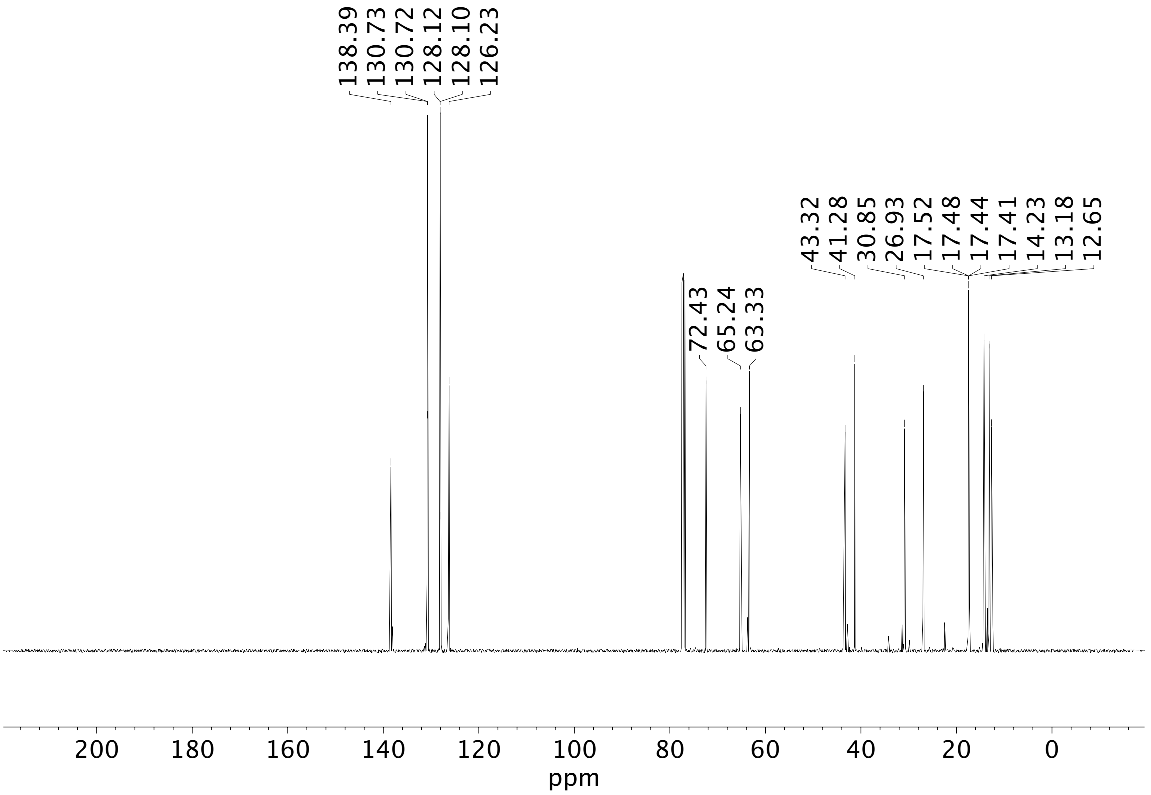

^{13}C NMR (100 MHz, $CDCl_3$) of compound **7**.

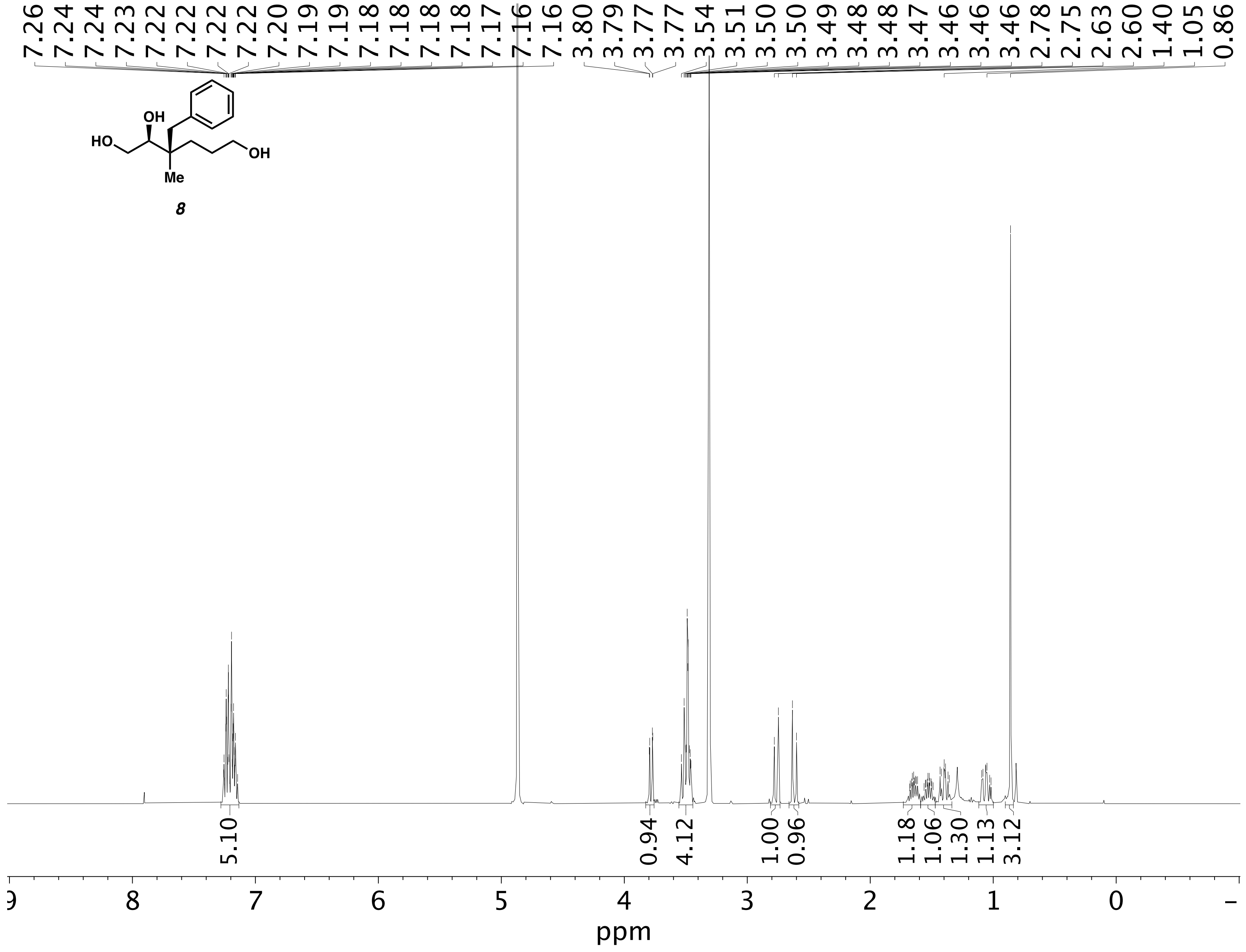

^{1}H NMR (400 MHz, CD_3OD) of compound **8**.

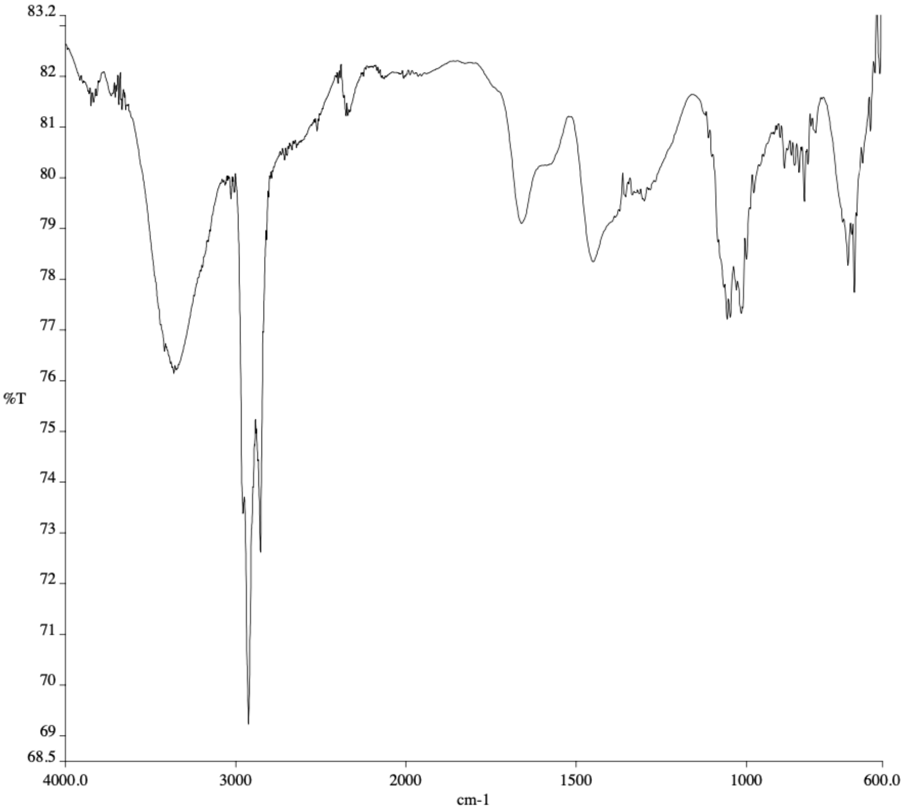

Infrared spectrum (Thin Film, NaCl) of compound **8**.

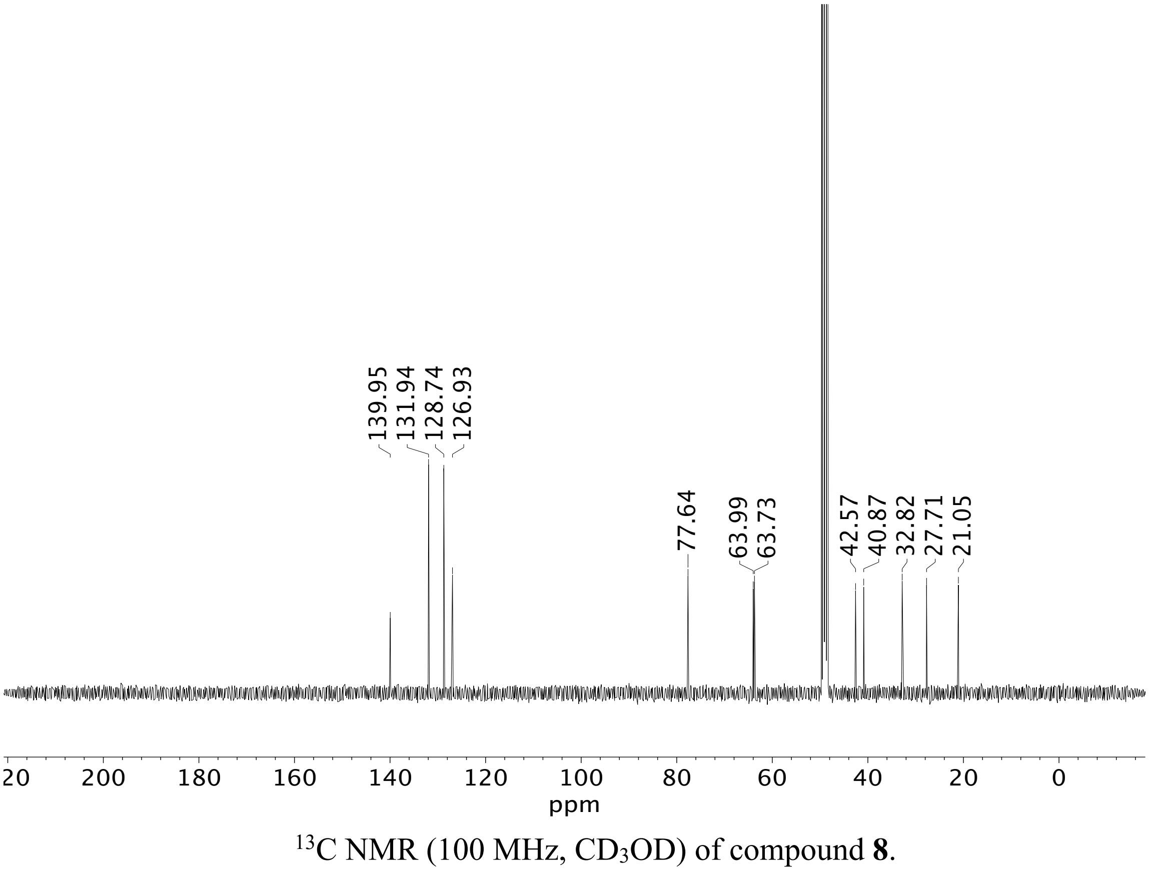

^{13}C NMR (100 MHz, CD_3OD) of compound **8**.

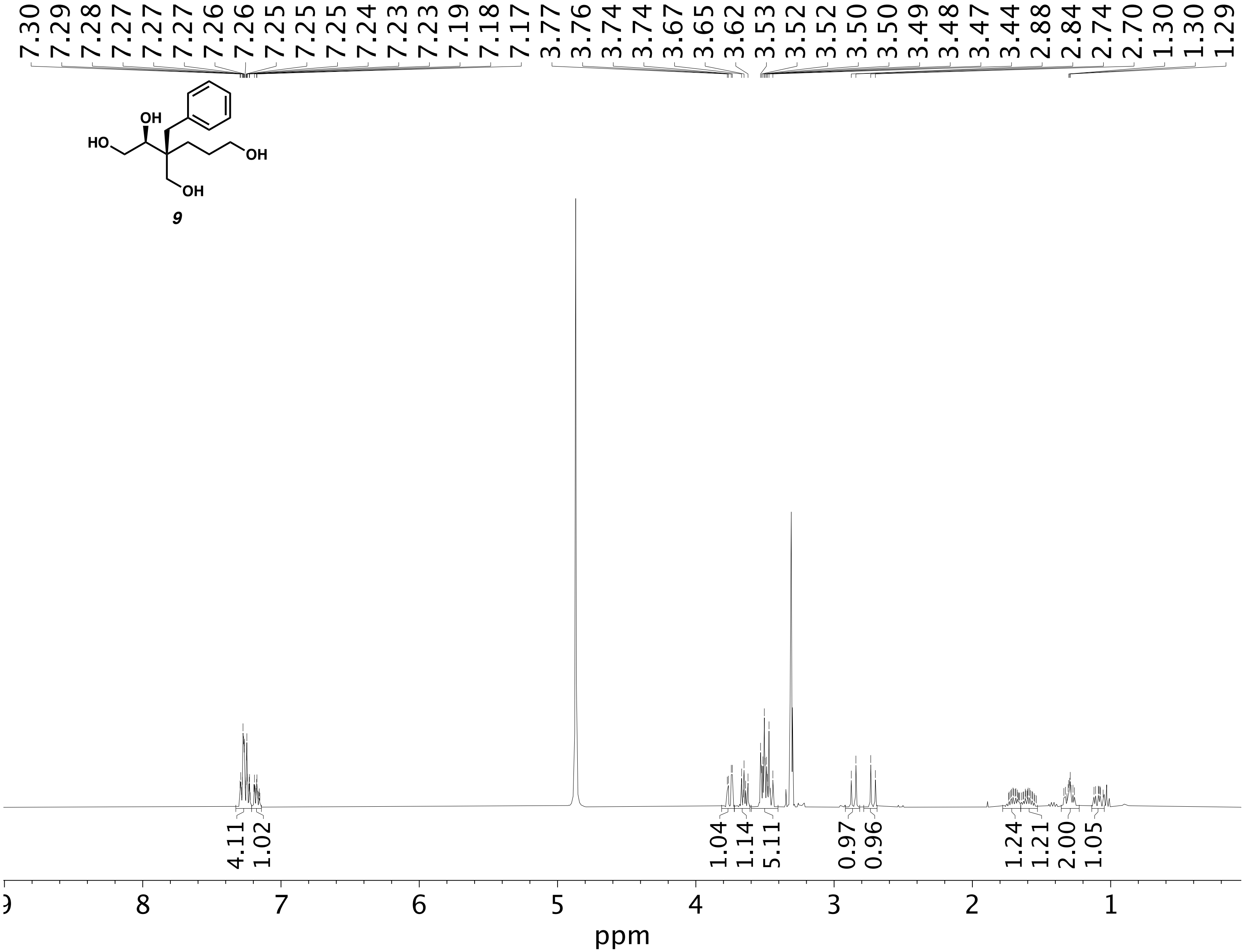

^1H NMR (400 MHz, CD_3OD) of compound **9**.

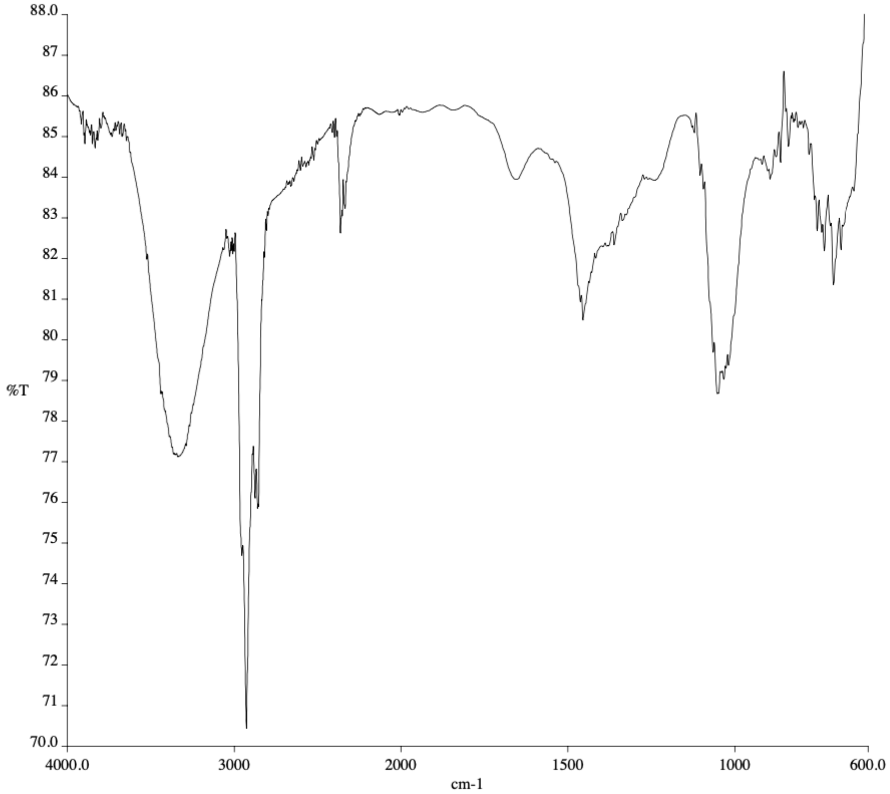

Infrared spectrum (Thin Film, NaCl) of compound **9**.

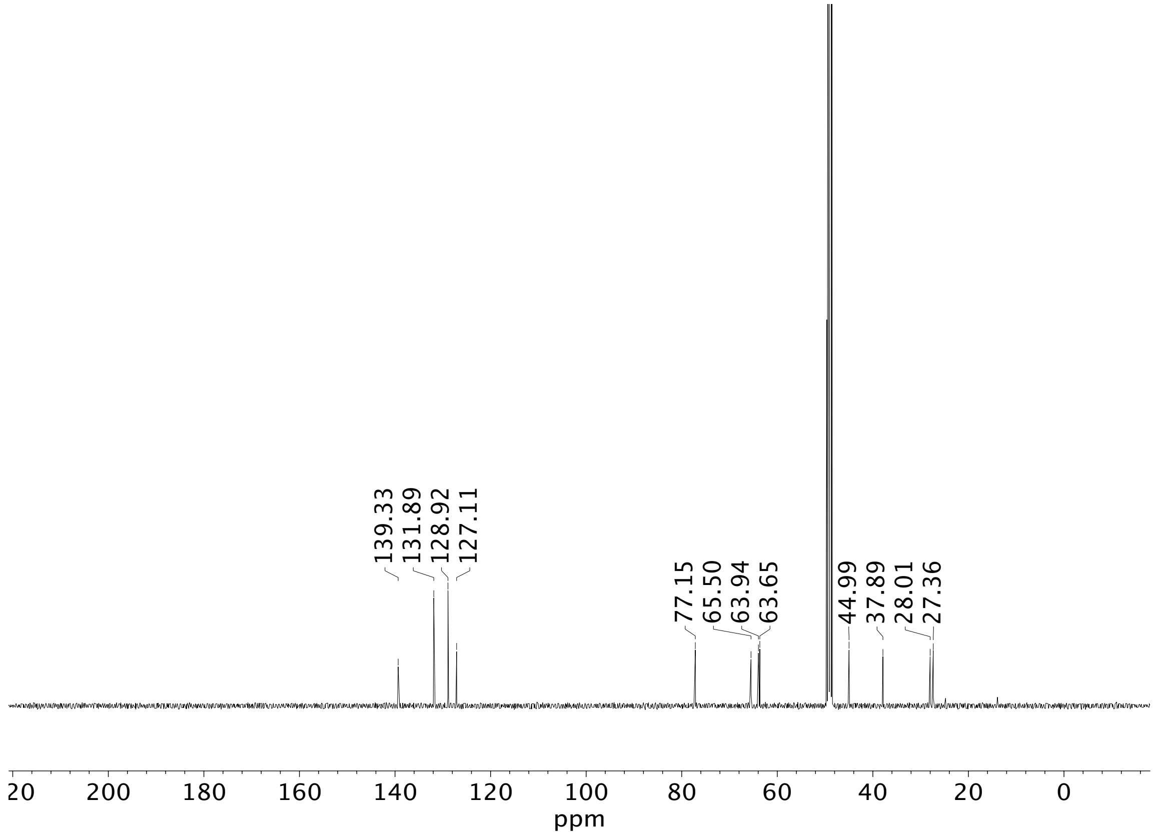

^{13}C NMR (100 MHz, CD_3OD) of compound **9**.

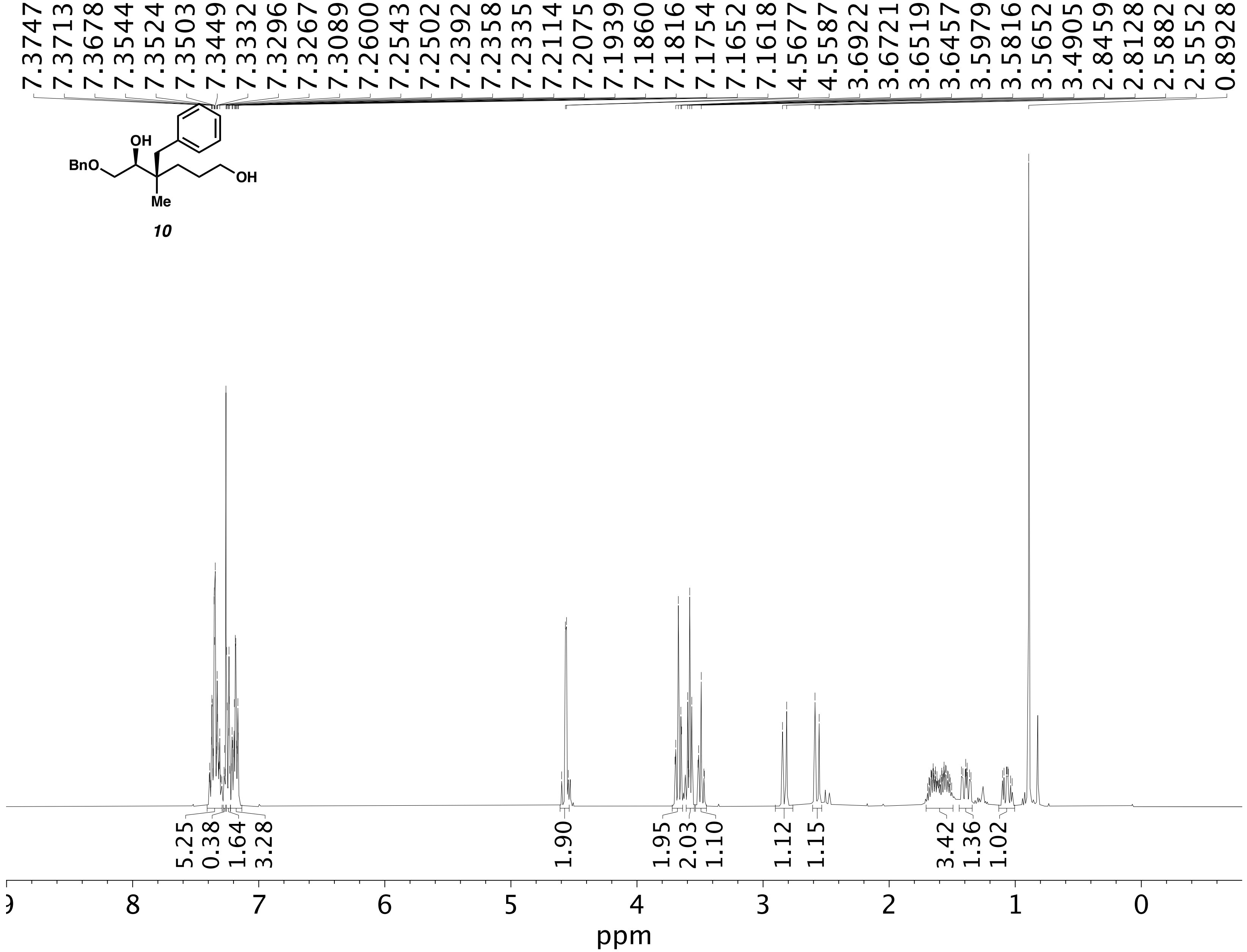

^{1}H NMR (400 MHz, $CDCl_3$) of compound **10**.

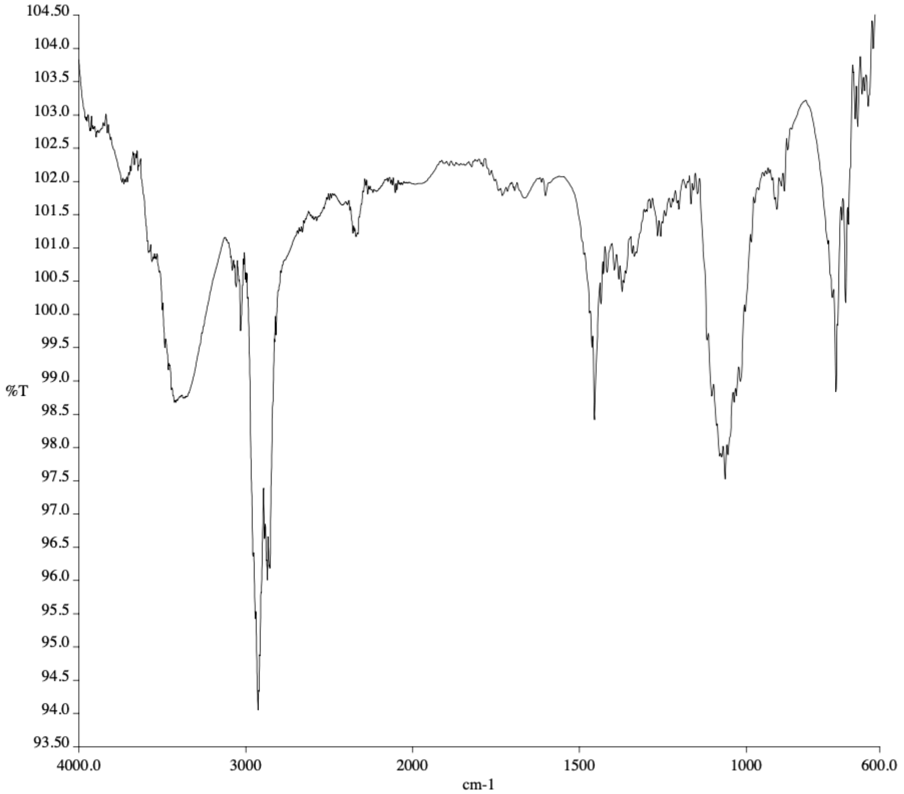

Infrared spectrum (Thin Film, NaCl) of compound **10**.

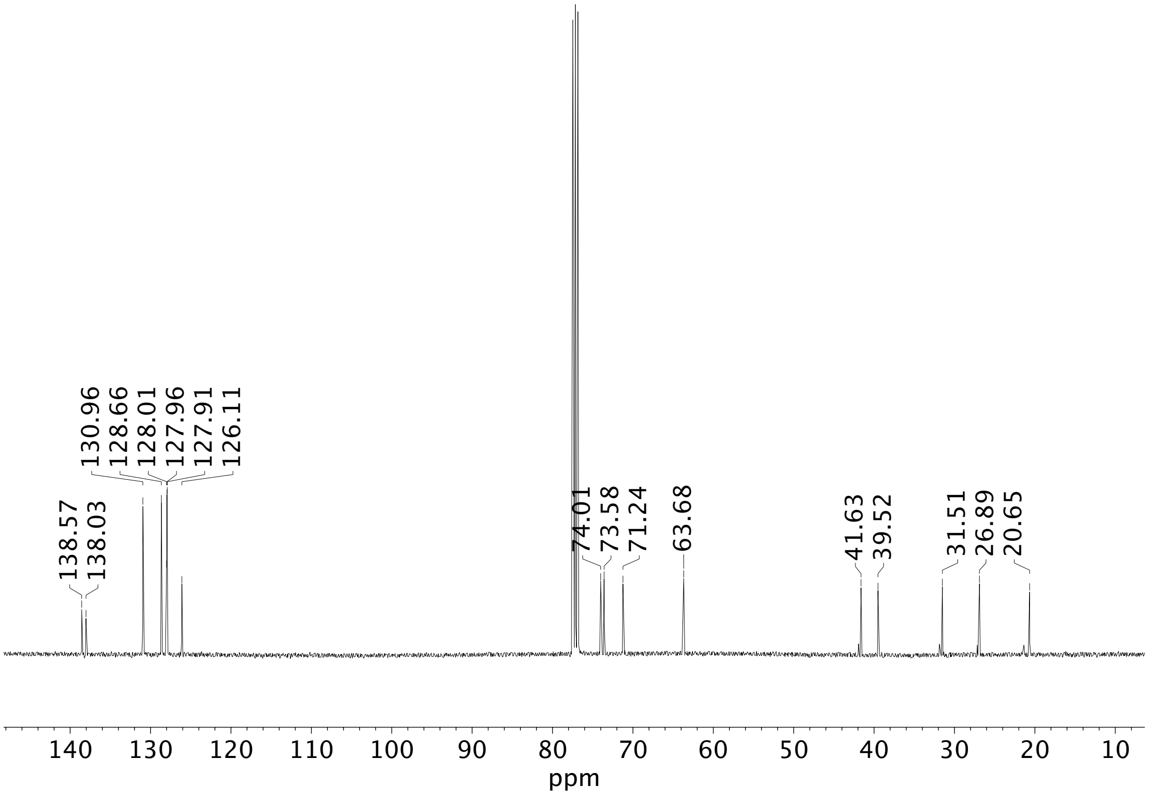

^{13}C NMR (100 MHz, $CDCl_3$) of compound **10**.

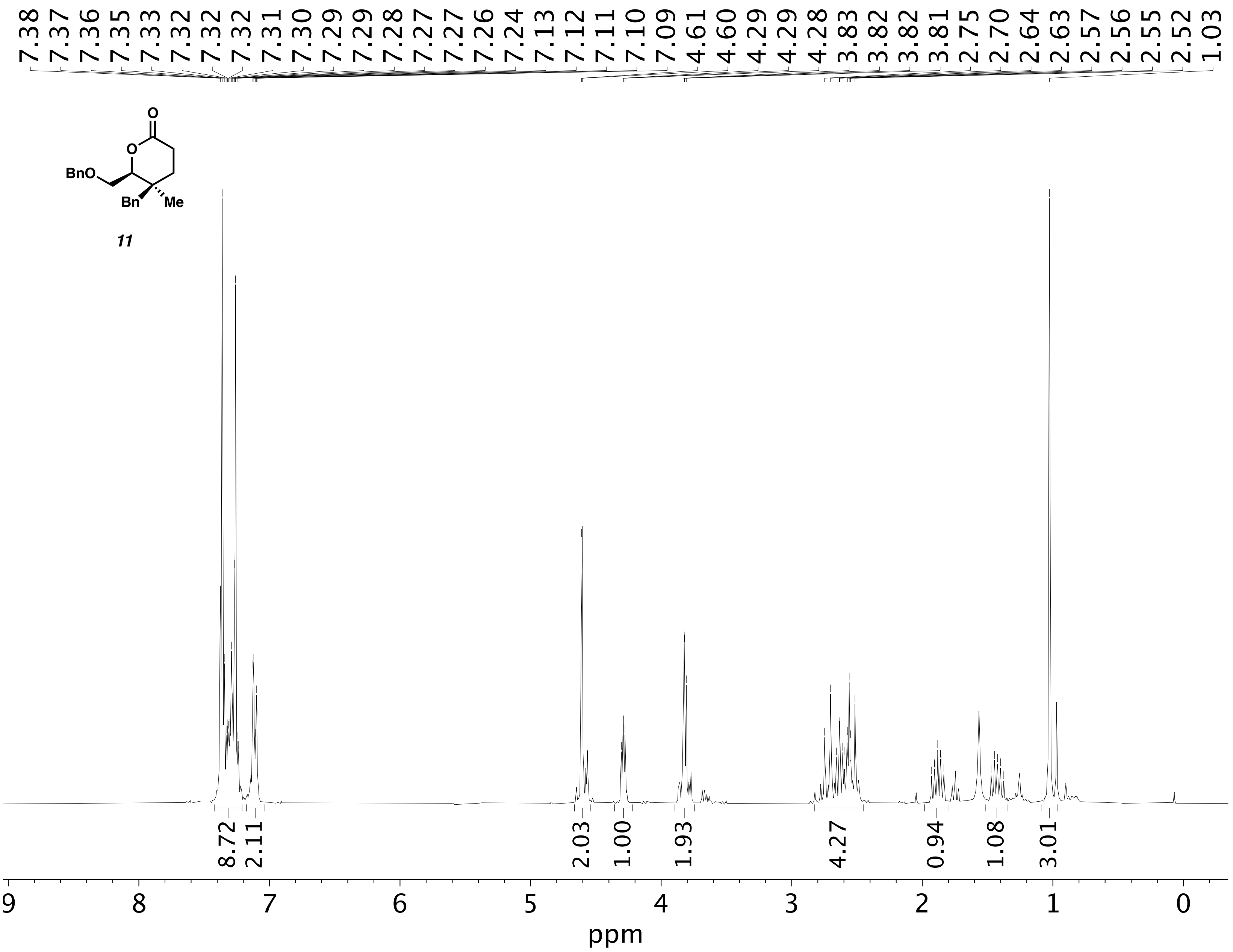

^{1}H NMR (400 MHz, $CDCl_3$) of compound **11**.

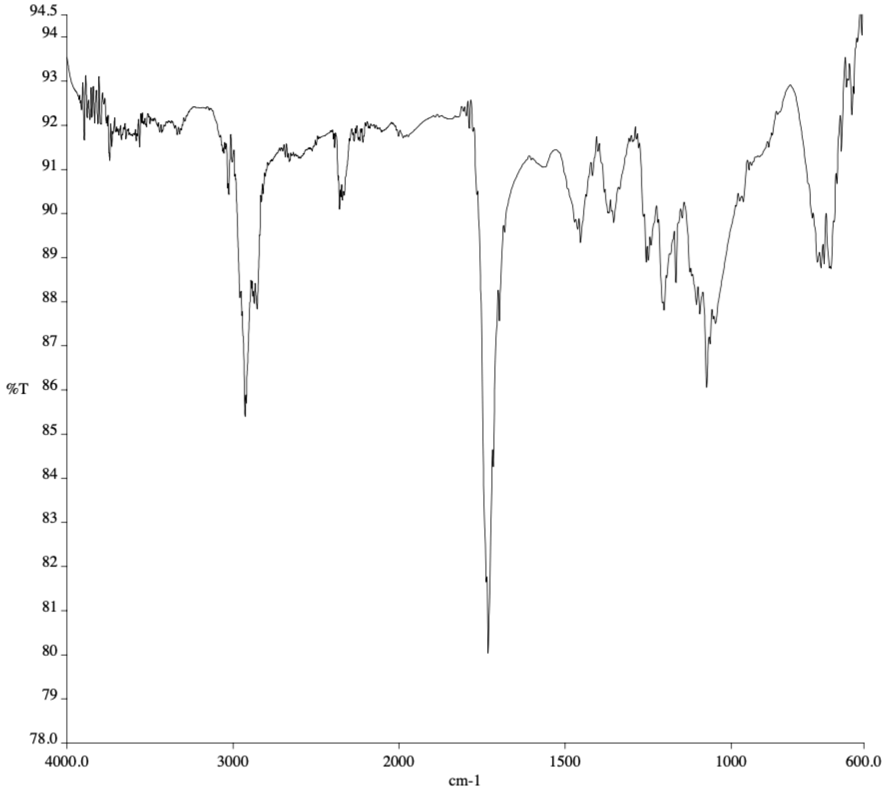

Infrared spectrum (Thin Film, NaCl) of compound **11**.

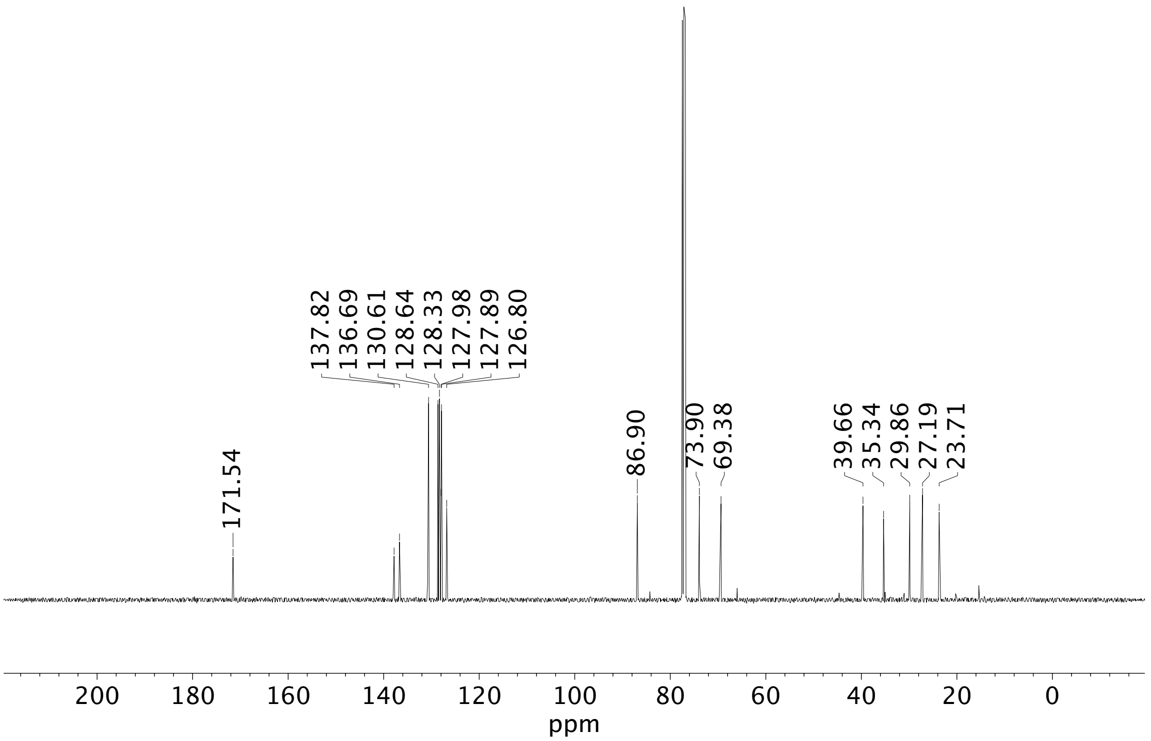

^{13}C NMR (100 MHz, $CDCl_3$) of compound **11**.

SFC Traces of Racemic and Enantioenriched Compounds

Racemic **6a**

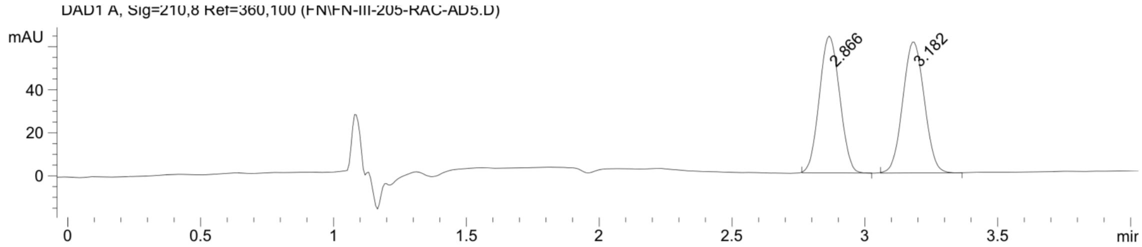

Signal 1: DAD1 A, Sig=210,8 Ref=360,100

Peak #	RetTime [min]	Type	Width [min]	Area [mAU*s]	Height [mAU]	Area %
1	2.866	BB	0.0840	338.24231	63.60172	49.6584
2	3.182	BB	0.0895	342.89578	61.03670	50.3416

Enantioenriched **6a**

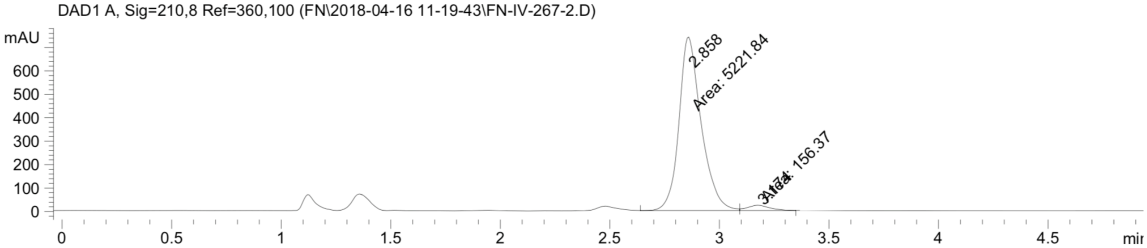

Signal 1: DAD1 A, Sig=210,8 Ref=360,100

Peak #	RetTime [min]	Type	Width [min]	Area [mAU*s]	Height [mAU]	Area %
1	2.858	MF	0.1173	5221.83545	742.07227	97.0925
2	3.174	FM	0.1149	156.37050	22.68277	2.9075

Racemic **6b**

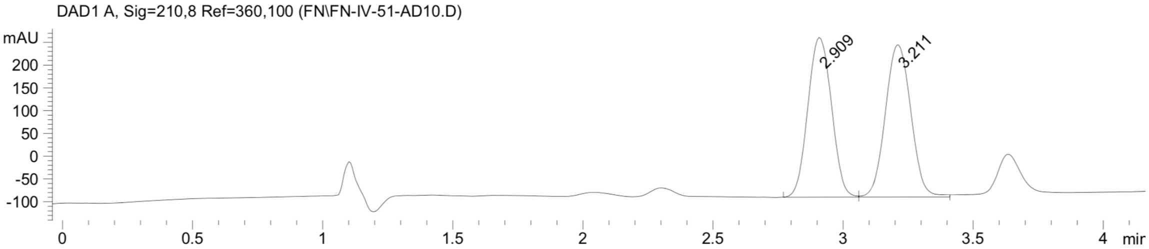

Signal 1: DAD1 A, Sig=210,8 Ref=360,100

Peak #	RetTime [min]	Type	Width [min]	Area [mAU*s]	Height [mAU]	Area %
1	2.909	BV	0.0980	2219.58398	349.91458	49.3293
2	3.211	VV	0.1077	2279.94360	333.81265	50.6707

Enantioenriched **6b**

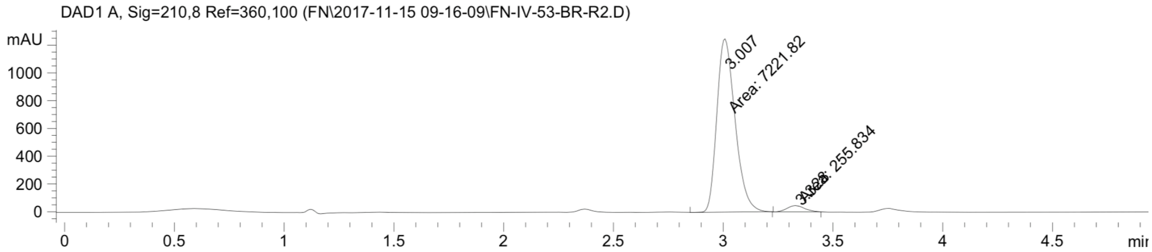

Signal 1: DAD1 A, Sig=210,8 Ref=360,100

Peak #	RetTime [min]	Type	Width [min]	Area [mAU*s]	Height [mAU]	Area %
1	3.007	MM	0.0963	7221.82129	1250.29602	96.5787
2	3.328	MM	0.0927	255.83408	46.00377	3.4213

Racemic **6c**

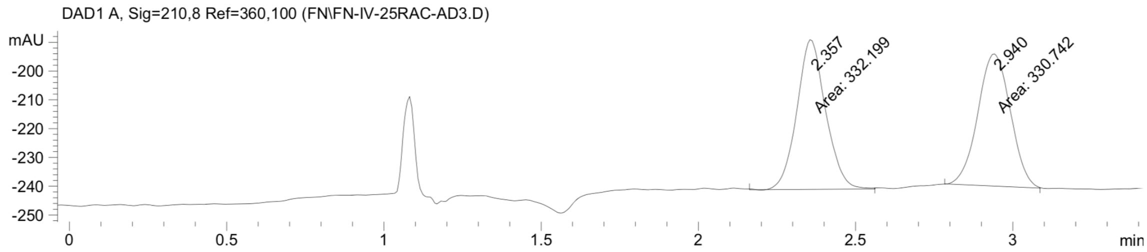

Signal 1: DAD1 A, Sig=210,8 Ref=360,100

Peak #	RetTime [min]	Type	Width [min]	Area [mAU*s]	Height [mAU]	Area %
1	2.357	MM	0.1062	332.19888	52.14556	50.1098
2	2.940	MM	0.1199	330.74246	45.98196	49.8902

Enantioenriched **6c**

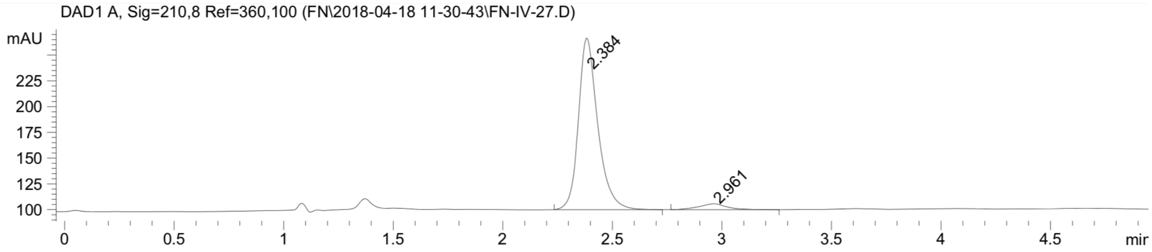

Signal 1: DAD1 A, Sig=210,8 Ref=360,100

Peak #	RetTime [min]	Type	Width [min]	Area [mAU*s]	Height [mAU]	Area %
1	2.384	BB	0.0918	1027.01538	166.85977	95.0357
2	2.961	BB	0.1319	53.64746	5.79819	4.9643

Racemic **6d**

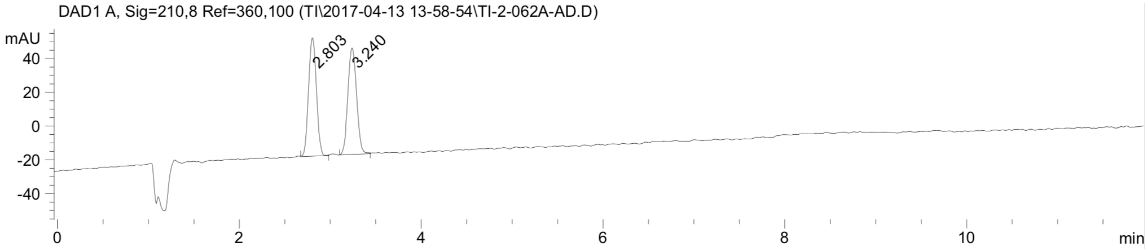

Signal 1: DAD1 A, Sig=210,8 Ref=360,100

Peak #	RetTime [min]	Type	Width [min]	Area [mAU*s]	Height [mAU]	Area %
1	2.803	BB	0.0961	422.21487	70.26080	49.9031
2	3.240	BB	0.1083	423.85532	63.18443	50.0969

Enantioenriched **6d**

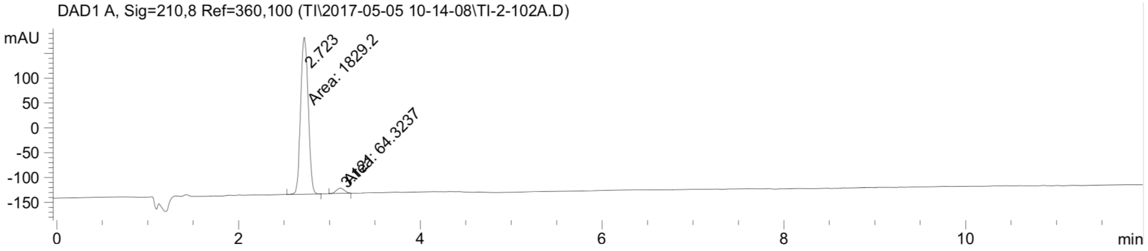

Signal 1: DAD1 A, Sig=210,8 Ref=360,100

Peak #	RetTime [min]	Type	Width [min]	Area [mAU*s]	Height [mAU]	Area %
1	2.723	MM	0.0963	1829.20435	316.43353	96.6030
2	3.121	MM	0.1003	64.32370	10.68824	3.3970

Racemic **6e**

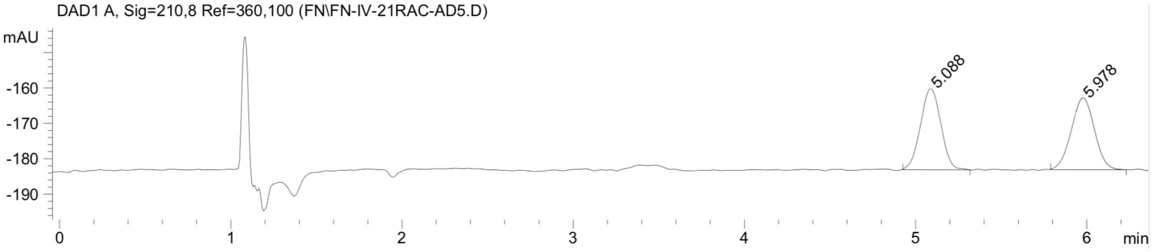

Signal 1: DAD1 A, Sig=210,8 Ref=360,100

Peak #	RetTime [min]	Type	Width [min]	Area [mAU*s]	Height [mAU]	Area %
1	5.088	BB	0.1307	190.40477	22.96074	49.8502
2	5.978	BB	0.1459	191.54884	20.34098	50.1498

Enantioenriched **6e**

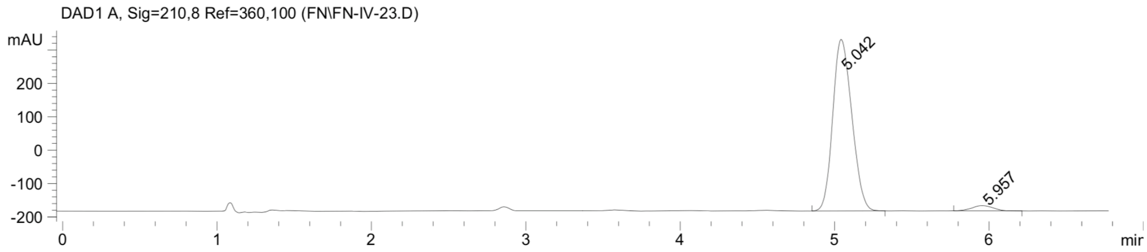

Signal 1: DAD1 A, Sig=210,8 Ref=360,100

Peak #	RetTime [min]	Type	Width [min]	Area [mAU*s]	Height [mAU]	Area %
1	5.042	BB	0.1342	4418.93457	514.27942	96.6490
2	5.957	BB	0.1481	153.21478	15.94727	3.3510

Racemic **6f**

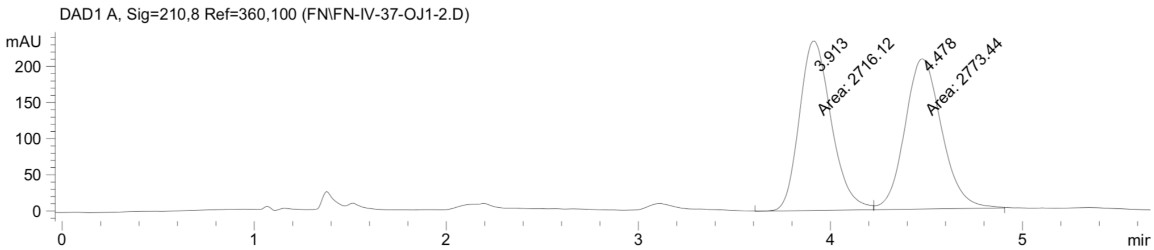

Signal 1: DAD1 A, Sig=210,8 Ref=360,100

Peak #	RetTime [min]	Type	Width [min]	Area [mAU*s]	Height [mAU]	Area %
1	3.913	MF	0.1925	2716.11743	235.16719	49.4779
2	4.478	FM	0.2221	2773.44092	208.13399	50.5221

Enantioenriched **6f**

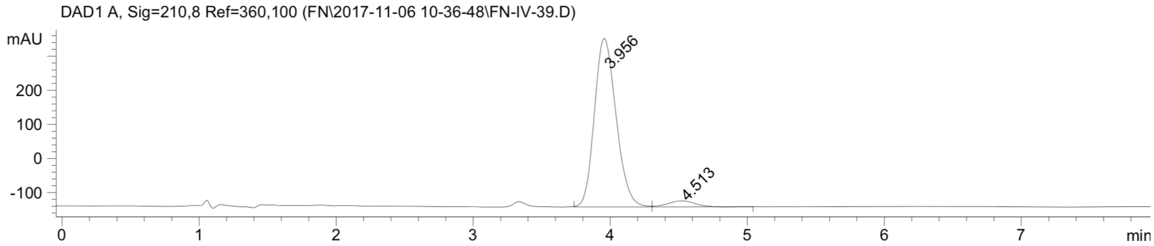

Signal 1: DAD1 A, Sig=210,8 Ref=360,100

Peak #	RetTime [min]	Type	Width [min]	Area [mAU*s]	Height [mAU]	Area %
1	3.956	BV	0.1701	5432.19678	494.60312	95.7945
2	4.513	VB	0.2086	238.48030	17.77107	4.2055

Racemic **6g**

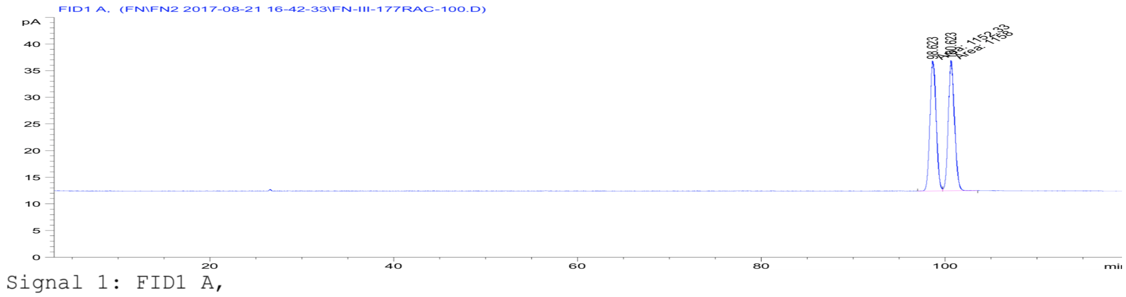

Signal 1: FID1 A,

Peak #	RetTime [min]	Type	Width [min]	Area [pA*s]	Height [pA]	Area %
1	98.623	MF	0.7882	1152.32947	24.36778	49.87734
2	100.623	FM	0.7880	1157.99719	24.49116	50.12266

Enantioenriched **6g**

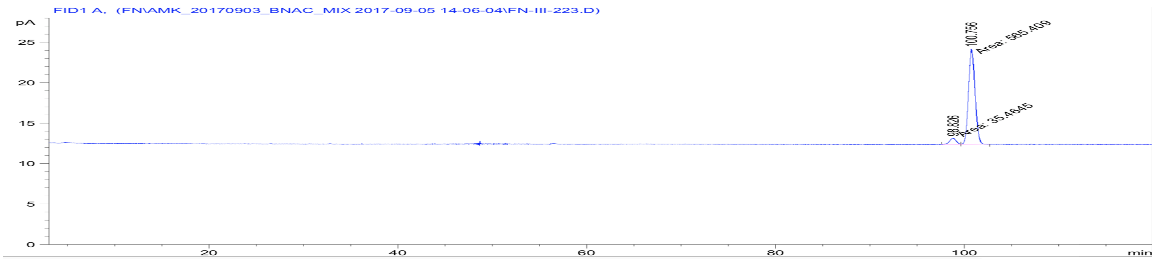

Signal 1: FID1 A,

Peak #	RetTime [min]	Type	Width [min]	Area [pA*s]	Height [pA]	Area %
1	98.826	MF	0.7611	35.46446	7.76600e-1	5.90215
2	100.756	FM	0.7976	565.40918	11.81410	94.09785

Racemic **6h**

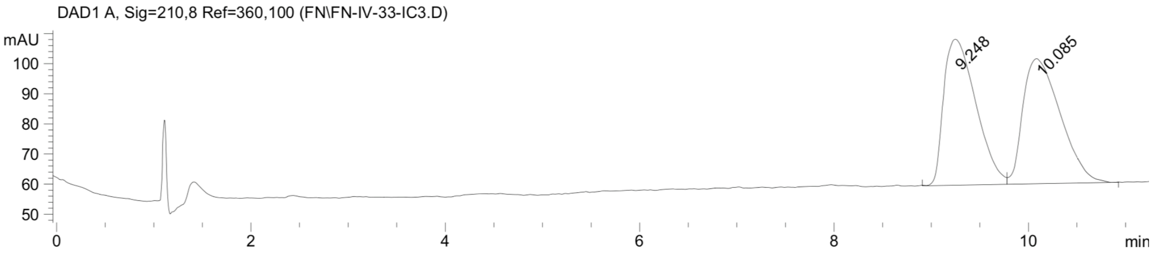

Signal 1: DAD1 A, Sig=210,8 Ref=360,100

Peak #	RetTime [min]	Type	Width [min]	Area [mAU*s]	Height [mAU]	Area %
1	9.248	BV	0.3509	1117.05652	48.46346	50.1248
2	10.085	VB	0.4103	1111.49524	41.52903	49.8752

Enantioenriched **6h**

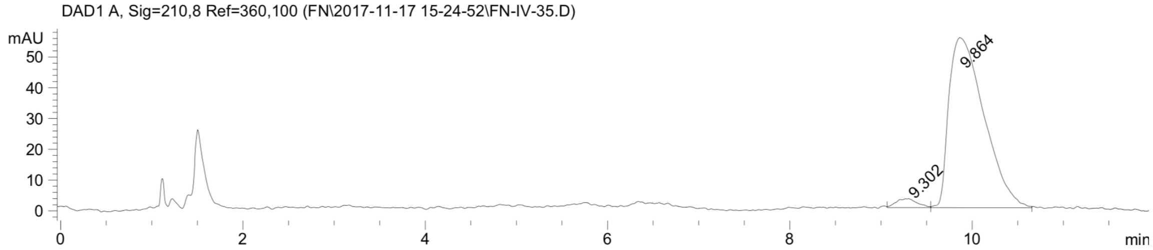

Signal 1: DAD1 A, Sig=210,8 Ref=360,100

Peak #	RetTime [min]	Type	Width [min]	Area [mAU*s]	Height [mAU]	Area %
1	9.302	VV	0.1923	46.26558	2.89692	3.0315
2	9.864	VB	0.4084	1479.89392	55.26742	96.9685

Racemic **6i**

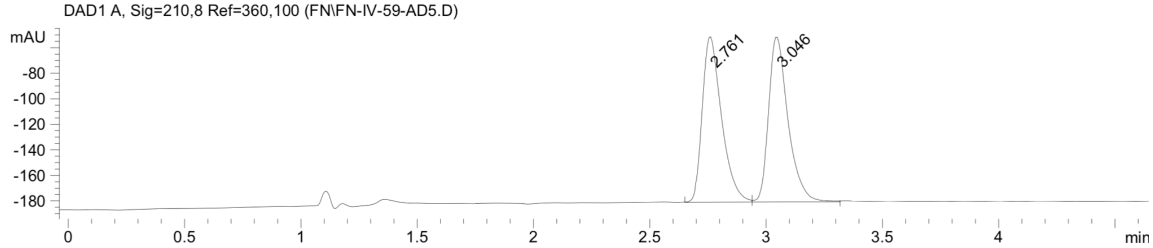

Signal 1: DAD1 A, Sig=210,8 Ref=360,100

Peak #	RetTime [min]	Type	Width [min]	Area [mAU*s]	Height [mAU]	Area %
1	2.761	BV	0.0873	747.56964	129.50545	49.8887
2	3.046	VB	0.0924	750.90533	128.01891	50.1113

Enantioenriched **6i**

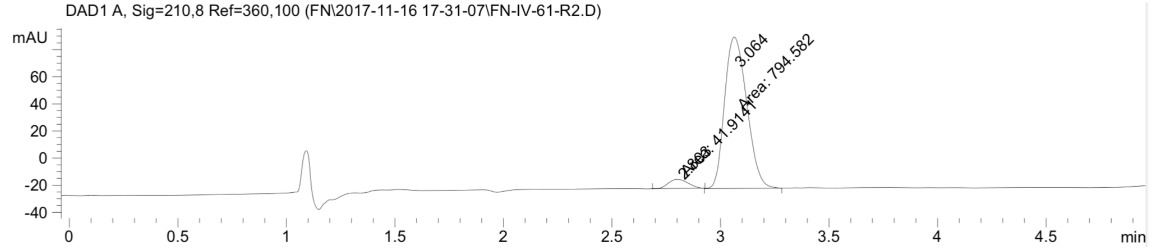

Signal 1: DAD1 A, Sig=210,8 Ref=360,100

Peak #	RetTime [min]	Type	Width [min]	Area [mAU*s]	Height [mAU]	Area %
1	2.803	MM	0.1041	41.91414	6.70925	5.0107
2	3.064	MM	0.1180	794.58179	112.21146	94.9893
